# Identification and Characterization of *DAM*s Mutations Associated With Early Blooming in Sweet Cherry, and Validation of DNA-Based Markers for Selection

**DOI:** 10.3389/fpls.2021.621491

**Published:** 2021-07-08

**Authors:** Alejandro Calle, Jérôme Grimplet, Loïck Le Dantec, Ana Wünsch

**Affiliations:** ^1^Unidad de Hortofruticultura, Centro de Investigación y Tecnología Agroalimentaria de Aragón, Zaragoza, Spain; ^2^Instituto Agroalimentario de Aragón-IA2, CITA-Universidad de Zaragoza, Zaragoza, Spain; ^3^Univ. Bordeaux, INRAE, Biologie du Fruit et Pathologie, UMR 1332, Villenave d'Ornon, France

**Keywords:** *Prunus avium* L, chill requirement, blooming, *DAM*s, gene expression regulation, non-coding gene, UTRs, breeding

## Abstract

Dormancy release and bloom time of sweet cherry cultivars depend on the environment and the genotype. The knowledge of these traits is essential for cultivar adaptation to different growing areas, and to ensure fruit set in the current climate change scenario. In this work, the major sweet cherry bloom time QTL *qP-BT1.1*^*m*^ (327 Kbs; Chromosome 1) was scanned for candidate genes in the Regina cv genome. Six MADS-box genes (*PavDAM*s), orthologs to peach and Japanese apricot *DAM*s, were identified as candidate genes for bloom time regulation. The complete curated genomic structure annotation of these genes is reported. To characterize *PavDAM*s intra-specific variation, genome sequences of cultivars with contrasting chilling requirements and bloom times (*N* = 13), were then mapped to the ‘Regina’ genome. A high protein sequence conservation (98.8–100%) was observed. A higher amino acid variability and several structural mutations were identified in the low-chilling and extra-early blooming cv Cristobalina. Specifically, a large deletion (694 bp) upstream of *PavDAM1*, and various INDELs and SNPs in contiguous *PavDAM4* and *-5* UTRs were identified. *PavDAM1* upstream deletion in ‘Cristobalina’ revealed the absence of several cis-acting motifs, potentially involved in *PavDAM*s expression. Also, due to this deletion, a non-coding gene expressed in late-blooming ‘Regina’ seems truncated in ‘Cristobalina’. Additionally, *PavDAM4* and -*5* UTRs mutations revealed different splicing variants between ‘Regina’ and ‘Cristobalina’ *PavDAM5*. The results indicate that the regulation of *PavDAM*s expression and post-transcriptional regulation in ‘Cristobalina’ may be altered due to structural mutations in regulatory regions. Previous transcriptomic studies show differential expression of *PavDAM* genes during dormancy in this cultivar. The results indicate that ‘Cristobalina’ show significant amino acid differences, and structural mutations in *PavDAM*s, that correlate with low-chilling and early blooming, but the direct implication of these mutations remains to be determined. To complete the work, PCR markers designed for the detection of ‘Cristobalina’ structural mutations in *PavDAM*s, were validated in an F_2_ population and a set of cultivars. These PCR markers are useful for marker-assisted selection of early blooming seedlings, and probably low-chilling, from ‘Cristobalina’, which is a unique breeding source for these traits.

## Introduction

Adequate blooming and pollination are essential for fruit set in sweet cherry (*Prunus avium* L.) and other fruit tree species. Temperate climate fruit trees such as sweet cherry go through a dormancy period in which meristem growth is inactive (Lang et al., 1987; Rohde and Bhalerao, 2007). This occurs before the blooming season to prevent winter damage due to frost and low temperatures. Dormancy is divided into three stages: paradormancy and endodormancy, in which bud growth is inhibited during autumn and winter seasons, and ecodormancy, in which bud growth is resumed under more favorable climatic conditions in late winter and early spring (Lang et al., 1987). The length of the dormant period depends on the environmental temperatures since determined amounts of chill and heat (Chilling and Heat requirements) are needed to complete endodormancy and ecodormancy before bud burst (Cooke et al., 2012). These requirements are specific to each genotype and vary according to the environmental conditions (Alburquerque et al., 2008). Both chilling and heat requirements influence blooming, however, several studies in *Prunus* species have reported that chilling requirement is the major determinant of bloom time (Alburquerque et al., 2008; Fan et al., 2010; Campoy et al., 2011; Castède et al., 2014).

Dormancy release, chilling requirement, and bloom time are relevant traits for cultivar adaptation to the growing area and to ensure an adequate fruit set. As many cherry cultivars are self-incompatible, blooming has to be synchronized between cultivars planted in the same vicinity. Late season blooming allows for the avoidance of spring frosts in cold regions. Cultivars with low chilling requirements are useful to adapt to temperature rise in the actual context of climate change. Additionally, cultivars with low chilling requirements can be used to extend cultivation to warmer areas, thus extending cultivation further away from traditional cultivation regions. Several works have investigated the physiology and the genetics of these traits in sweet cherry and other fruit tree species (reviewed in Abbott et al., 2015; and Fadón and Rodrigo, 2018). In sweet cherry, genetic analyses have revealed that bloom time is a quantitative trait with very high heritability (Dirlewanger et al., 2012; Castède et al., 2014; Calle et al., 2020). In this species, major quantitative trait loci (QTLs) associated with bloom time have been identified on linkage groups (LGs) 1, 2, and 4 (Dirlewanger et al., 2012; Castède et al., 2014; Calle et al., 2020). In other *Prunus* species, like almond (*Prunus amygdalus* L.), peach [*Prunus persica* (L). Batsch] and Japanese apricot (*Prunus mume* L.), main bloom time QTLs have also been mapped on the orthologous regions of LG1 (Fan et al., 2010; Zhebentyayeva et al., 2014; Bielenberg et al., 2015) and LG4 (Dirlewanger et al., 2012; Sánchez-Pérez et al., 2012; Kitamura et al., 2018). In the same region of LG1, stable and significant QTLs associated with chilling requirements in almond, peach, and sweet cherry have also been detected (Fan et al., 2010; Sánchez-Pérez et al., 2012; Castède et al., 2014; Bielenberg et al., 2015). This LG1 QTL region overlaps with a deletion in the *evergrowing* (*EVG*) peach mutant, which does not enter dormancy (Rodriguez et al., 1994). A tandem repeat of six MADS-box genes, named *dormancy-associated MADS-box* (*DAM*), was identified in this LG1 region, four of them being deleted in the *EVG* mutant (Bielenberg et al., 2008), reveling the potential involvement of these genes in dormancy control of *Prunus* species. In sweet cherry, *DAM5* and *-6* have also been mapped on LG1, overlapping with the main bloom time and chilling requirement QTL of this LG (Castède et al., 2015). In other *Rosaceous* species, like apple and pear, a variable number of *DAM* gene have also been reported (Saito et al., 2013; Mimida et al., 2015), some of them overlapping with regions in which bloom time QTLs for these species were found (Allard et al., 2016).

In different plant species, MADS-box transcription factors have been reported as strong candidate genes for the genetic control of blooming and temperature responses (Gramzow and Theissen, 2010). MADS-box genes play fundamental roles in pathways involved in the transition from vegetative to reproductive phases, growth, floral organ determination, and other processes related to root, leaf, fruit, and gametophyte development (Becker and Theißen, 2003; Messenguy and Dubois, 2003; Smaczniak et al., 2012). The *DAM* genes reported in sweet cherry, peach, Japanese apricot, and European plum (*Prunus domestica* L.) belong to MIKC^c^ Type II of MADS-box genes and are phylogenetically related to *Arabidopsis SHORT VEGETATIVE PHASE* (*SVP*) and *AGAMOUS*-*LIKE 24* (*AGL24*) genes, which have been reported as main floral regulators (Jiménez et al., 2009; Sasaki et al., 2011; Quesada-Traver et al., 2020; Wang et al., 2020). Analyses of *DAM* gene expression levels in *Prunus* species have shown a similar pattern in different years and correlations with photoperiod and temperature changes (Falavigna et al., 2019), suggesting that these genes are the main regulators of the dormancy cycle in *Prunus* species (Yamane, 2014). Maximum expression levels of *DAM1* to *-4* were observed during bud set, suggesting a role in the regulation of growth cessation and bud formation in peach and Japanese apricot (Li et al., 2009; Sasaki et al., 2011; Zhang et al., 2018). On the other side, *DAM5* and *-6* showed the highest expression level in the winter season during induction and maintenance of dormancy and minimal or absent expression during the budbreak and bloom time (Jiménez et al., 2010; Yamane et al., 2011; Leida et al., 2012; Prudencio et al., 2018). Therefore, down-regulation of *DAM5* and -*6* during the winter season, with minimum expression level when chilling requirements are fulfilled, is compatible with the role of dormancy release repressor of *DAM* genes in *Prunus* species (Sasaki et al., 2011). In sweet cherry, expression patterns of these genes have been reported. *DAM1, -3*, and *-6* were highly expressed during paradormancy and at the beginning of endodormancy, whereas *DAM4* and *-5* showed an expression peak at the end of endodormancy and chilling requirement fulfillment (Vimont et al., 2019, 2020; Villar et al., 2020; Wang et al., 2020). In the low chilling requirement, sweet cherry cultivar ‘Cristobalina’, a low expression level of *PavDAM1, PavDAM4*, and *PavDAM5* during the dormancy period was observed compared with the expression pattern of high chill-requirements cultivars for these genes (Vimont et al., 2019). Similarly, in other low chilling sweet cherry cultivar, ‘Royal Lee’, a low expression level of *PavDAM1*, especially during the chilling accumulation, compared with the expression pattern of high chill-requirement cultivar was reported (Wang et al., 2020). Epigenetic modification and the evolution of transcript levels during dormancy were evaluated for *DAM3* and -*5* in the sweet cherry cultivar ‘Bing’ (Rothkegel et al., 2017), revealing the involvement of siRNAs and DNA methylations in the silencing of *DAM3* during chilling accumulation and dormancy release.

In Calle et al. (2020), bloom time in sweet cherry was evaluated during 4 years using a multi-family QTL approach. In this work plant materials included populations that descend from cultivars with very low to high chilling requirements. These populations derive from self- and cross-pollination of ‘Cristobalina’, a cultivar with very low chilling requirement (<500 h) and extra-early flowering and maturity dates (Tabuenca, 1983; Alburquerque et al., 2008; Calle and Wünsch, 2020; Calle et al., 2020). This cultivar is of breeding interest due to these traits and other relevant characters like self-compatibility (Wünsch and Hormaza, 2004; Ono et al., 2018). Bloom time QTL analysis for these plant materials revealed that the highest percentage of phenotypic variation was explained by QTLs on LGs 1 (*qP-BT1.1*^*m*^) and 2 (*qP-BT2.1*^*m*^). The QTL detected on LG1 overlaps with a chilling requirement QTL previously reported on *Prunus* LG1 (Fan et al., 2010; Sánchez-Pérez et al., 2012; Castède et al., 2014; Bielenberg et al., 2015), and with *DAM* genes mapped in this region in sweet cherry, Japanese apricot and peach (Bielenberg et al., 2008; Sasaki et al., 2011; Castède et al., 2015). Moreover, haplotype analyses of this QTL showed that ‘Cristobalina’ was the only cultivar with alleles contributing to early blooming (Calle et al., 2020). Since early blooming in this plant material is believed to be due to low chilling requirements in ‘Cristobalina’, candidate genes from these QTLs may be involved in chilling requirement control.

The objective of this work is to confirm and characterize *DAM* genes as candidate genes in sweet cherry major bloom time QTL on LG1 using the sweet cherry genome sequence recently available, to investigate the genomic structure of these genes in the species, and to uncover variation of these candidate genes in cultivars with contrasting chilling requirements and bloom times (including ‘Cristobalina’). Intraspecific variation of *PavDAM* genes has not been previously investigated in sweet cherry and this study may allow us to identify polymorphisms associated with the phenotypic variation in the plant material studied. This knowledge may be further used to develop markers for assisted selection of these traits from this plant material. Furthermore, these results may help to broaden the understanding of dormancy regulation in sweet cherry and other *Prunus* species by improving our knowledge of these candidate genes.

## Materials and Methods

### Plant Materials and Sequence Resources

#### Sequence Resources

Sweet cherry cultivar ‘Regina’ genome (Le Dantec et al., 2020) was used for QTL candidate gene mining and annotation. This genome was also employed in the rest of the experiments as a sweet cherry reference genome. For phylogenetic analysis, nucleotide sequences of peach *DAM* genes [*PpeDAM1* (ABJ96361), *PpeDAM2* (ABJ96363), *PpeDAM3* (ABJ96364), *PpeDAM4* (ABJ96358), *PpeDAM5* (ABJ96359), and *PpeDAM6* (ABJ96360)] and Japanese apricot *DAM* genes [*PmuDAM1* (BAK78921), *PmuDAM2* (BAK78922), *PmuDAM3* (BAK78923), *PmuDAM4* (BAK78924), *PmuDAM5* (BAK78920), and *PmuDAM6* (BAH22477)] were compared. For the study of intraspecific variation of *PavDAM*s, the genome sequences of 13 cultivars (Illumina HiSeq 2500 and 4000 systems; DDBJ; SRA bioproject ID PRJDB6734), previously generated by Ono et al. (2018), were downloaded and aligned to the reference genome.

#### Plant Materials

For *PavDAM* mutation characterization and marker validation, two sets of plant materials were used. One is a collection of sweet cherry cultivars (*N* = 72; Table 1), from “CITA de Aragón” cultivar and germplasm collection (Zaragoza, Spain). This sample includes landraces and bred cultivars from different genetic backgrounds and variable chilling requirements and bloom dates. Some of these cultivars were only utilized for marker validation, while others were also analyzed for *PavDAM* mutation characterization (see Table 1). The other set of plant materials is an F_2_ population (B×C2; *N* = 61) from the self-pollination of selection ‘BC8’ (‘Brooks’ × ‘Cristobalina’; Calle et al., 2020). This population was only used for marker validation. Genomic DNA from all plant materials evaluated for genetic analyses was extracted from young leaves using DNeasy Plant Mini kit (Qiagen, MD, USA). DNA quantity and quality were assayed using NanoDrop ND-1000 spectrophotometer (Thermo Scientific, DE, USA).

**Table 1:** PavD1UM and PavD4/5M (*PavDAM1* and *PavDAM4,-5* structural mutations, respectively) PCR marker genotypes of 72 sweet cherry cultivars and accessions.

**Cultivar**	**Pedigree^**a**^ and origin**	**Bloom time^**b**^**	**Chilling req. (Chilling hours)^**c**^**	**PavD1UM (**~**size bp)**	**PavD4/5M (**~**size bp)**	**Experimental section (*as sequence resources*/as plant materials)**
Cristobalina	Unknown (Spain)	Extra-early	176	900	850	*IV*, CSM, VDM
Royal Lee	6HB488 o.p. (USA)	Extra-early	400	900	850	VDM
Temprana de Sot	Unknown (Spain)	Extra-early		900	850	VDM
Son Perot	Unknown (Spain)	Early		900/1600	750/850	VDM
BC-8	Brooks × Cristobalina (Spain)	Early		900/1600	750/850	VDM
De Mango Largo	Unknown (Spain)	Medium		900/1600	750/850	VDM
Brooks	Rainier × Early Burlat (USA)	Early	411	1600	750	*IV*, VDM
Burlat	Unknown (France)	Early	618	1600	750	VDM
Chinook	Bing × Gilpeck (USA)	Early		1600	750	VDM
Corum	(USA)	Early		1600	750	VDM
De Angelin	Unknown (Spain)	Early		1600	750	VDM
Earlise	Starking Hardy Giant × Burlat (France)	Early	981	1600	750	VDM
Early Bigi		Early		1600	750	VDM
Fercer	Stark Hardy Giant o.p. (France)	Early		1600	750	VDM
Lapins	Van × Stella (Canada)	Early	450	1600	750	VDM
Larian	Lambert × (Bing × Bush Tartarian) (USA)	Early	450	1600	750	VDM
Newstar	Van × Stella (Canada)	Early	709	1600	750	VDM
Precoce Bernard	Unknown (France)	Early		1600	750	VDM
Prime Giant		Early		1600	750	VDM
Primulat	Fercer o.p. (France)	Early		1600	750	VDM
Rainier	Bing × Van (USA)	Early		1600	750	*IV*, VDM
Ramón Oliva	Unknown (France)	Early	900	1600	750	VDM
Rubi		Early	618	1600	750	VDM
Royalton	NY1725 o.p. (USA)	Early		1600	750	VDM
Samba	2E-84-10 × Stella 16A7 (Canada)	Early		1600	750	VDM
Sommerset	Van × Vic (USA)	Early		1600	750	VDM
Talegal Ahim	Unknown (Spain)	Early		1600	750	VDM
Talegal Almedijar	Unknown (Spain)	Early		1600	750	VDM
Tieton	Stella x Early Burlat (USA)	Early		1600	750	VDM
Tigre	Unknown (France)	Early	900	1600	750	VDM
Ambrunés	Unknown (Spain)	Medium	1000	1600	750	*IV*, VDM
Bing	Black Republican o.p. (USA)	Medium	1000	1600	750	VDM
Compact Stella	Irradiated Stella (Canada)	Medium		1600	750	VDM
Cristalina	Star × Van (Canada)	Medium		1600	750	VDM
Early Van Compact	Irradiated Van (Canada)	Medium		1600	750	VDM
Garrafal de Monzón	Unknown (Spain)	Medium		1600	750	VDM
Garrafal del Jerte	Unknown (Spain)	Medium		1600	750	VDM
Gilpeck	Napoleon × Giant (USA)	Medium		1600	750	VDM
Hartland	Windsor o.p. (USA)	Medium		1600	750	VDM
Llucmayor	Unknown (Spain)	Medium		1600	750	VDM
Pico Colorado	Unknown (Spain)	Medium	1000	1600	750	VDM
Pico Negro	Unknown (Spain)	Medium		1600	750	VDM
Ripoll	Unknown (Spain)	Medium		1600	750	VDM
Santina	Stella × Summit (Canada)	Medium		1600	750	VDM
Satonishiki	(Japan)	Medium		1600	750	*IV*, VDM
Sonata	Lapins ×2N-39-5 (Canada)	Medium		1600	750	VDM
Star	“Deacon” o.p.	Medium		1600	750	VDM
Sue	Bing × Schmidt (Canada)	Medium		1600	750	*IV*, VDM
Taleguera Brillante	Unknown (Spain)	Medium	1000	1600	750	VDM
Van Spur		Medium		1600	750	VDM
Van	“Empress Eugenie” o.p. (Canada)	Medium	1000	1600	750	VDM
Vega	Bing × Victor (Canada)	Medium		1600	750	VDM
Belge	Unknown (France)	Late		1600	750	VDM
BlackGold		Late		1600	750	VDM
Blanca de Provenza	Unknown (Unknown)	Late		1600	750	VDM
De la Rosa	Unknown (Spain)	Late		1600	750	VDM
Ferrovia	(Italy)	Late		1600	750	*IV*, VDM
Hedelfinger	Unknown (Germany)	Late	>1100	1600	750	*IV*, VDM
Lambert	Napoleon × Blackheart (USA)	Late	>1100	1600	750	*IV*, VDM
Garrafal de Lerida	Unknown (Spain)	Late		1600	750	VDM
Napoleon	Unknown	Late	>1100	1600	750	*IV*, VDM
Sandon Rose	(Canada)	Late		1600	750	VDM
Sylvia	Van × Sam (Canada)	Late		1600	750	VDM
Vic	Bing × Schmidt (Canada)	Late		1600	750	*IV*, VDM
Villalengua	Unknown (Spain)	Late		1600	750	VDM
Blanca Italiana	Unknown (Spain)	Very late		1600	750	VDM
Colney	Unknown (UK)	Very late		1600	750	VDM
Manola	Unknown (Spain)	Very late		1600	750	VDM
Margit	“Germersdorfer” o.p. (Hungary)	Very late		1600	750	VDM
Regina	Schneiders Späte Knorpel × Rube (Germany)	Very late	>1100	1600	750	*MAS, IV*, VDM, CSM
Sam	(Windsor o.p.) o.p. (Canada)	Very late		1600	750	*IV*, VDM
Summit	Van × Sam (Canada)	Very late		1600	750	*IV*, VDM

*Available data of pedigree, origin, chilling requirements, and bloom time of each cultivar/accession is also included*.

a *Data from Wünsch and Hormaza (2002) and Schuster (2012) except for ‘Royal Lee’ (data from US Patent No. 12417)*.

b *Data from Gella et al. (2001), Quero-García et al. (2017), and authors data*.

c *Data from Fadón et al. (2020)*.

*MAS, Mining, annotation, and structural analyses of candidate genes; IV, Intra-specific variation of PavDAMs; CSM, Characterization of PavDAMs structural mutations; VDM, Validation of DNA-markers of PavDAMs structural mutations; o.p., Open pollination*.

### Mining, Annotation, and Structural Analyses of Candidate Genes in Major Bloom Time QTL (*qP-BT1.1^*m*^*)

Coding DNA sequences of predicted genes in region Chr01_49296241:49622837 (326,596 bp) were extracted from ‘Regina’ sweet cherry genome. This region spans a previous main bloom time QTL in sweet cherry, *qP-BT1.1*^*m*^ (Calle et al., 2020). The protein sequences of the predicted genes annotated in this region were blasted against the NCBI non-redundant protein sequences (nr) database using the BLASTP algorithm to obtain the corresponding gene ontologies. For each gene, we searched for bibliographic evidence (annotation and predicted function) that led to information associated with their potential involvement in bloom time and chilling requirement. Curation of the structural annotation was performed from the ‘Regina’ genome annotation using BLAST analysis, motif detection, and public ‘Regina’ RNAseq data from Vimont et al. (2019). The original gene nomenclature was conserved.

Sweet cherry *DAM* genes sequences and the GFF (General Feature Format) annotation file containing the exon-intron structure of these genes were retrieved from the ‘Regina’ genome database. These files were uploaded into the Integrative Genomics Viewers (IGV) software (Thorvaldsdóttir et al., 2013) to double-check structure with their ortholog genes in peach genome v2.0.a1 (Verde et al., 2017). Manual sequence editing was done to correct the automatic annotation if needed, conserving an adequate intron splicing prediction.

### Phylogenetic Analysis of *PavDAM*s

Phylogenetic analysis of dormancy-associated MADS-box genes (*DAM1* to *6*) from peach (Bielenberg et al., 2008), Japanese apricot (Sasaki et al., 2011), and sweet cherry (this work) was conducted using MEGA X (Kumar et al., 2018). Multiple sequence alignment was carried out before tree construction using the MUSCLE algorithm (Edgar, 2004). The evolutionary history was inferred by using the Maximum Likelihood method and Tamura-Nei model (Tamura and Nei, 1993). Phylogenetic analysis was estimated using a bootstrap value of 1000, and the tree with the highest log likelihood was selected. Heuristic search for the initial tree was automatically obtained by using Neighbor Joining (NJ) and BioNJ algorithms to a matrix of pairwise distances estimated by the Maximum Composite Likelihood (MCL) approach, then the topology with superior log likelihood value was selected.

### Intraspecific Variation of *PavDAM*s Sequences in Cultivars With Large Phenotypic Variation

Genome sequences of 13 sweet cherry cultivars (‘Ambrunés’, ‘Brooks’, ‘Cristobalina’, ‘Ferrovia’, ‘Hedelfingen’, ‘Lambert’, ‘Napoleon’, ‘Rainier’, ‘Sam’, ‘Satonishiki’, ‘Sue’, ‘Summit’, and ‘Vic’) were used for genome sequence alignment. Genomic DNA-seq libraries (Ono et al., 2018), were downloaded and aligned using the Galaxy software framework (Afgan et al., 2018). Raw sequence data were processed using the SLIDINGWINDOW operation from Trimmomatic v0.36.6 (Bolger et al., 2014) to remove adapter sequences and to obtain clean sequence data. A FASTQ file for each cultivar containing clean reads was then aligned to the ‘Regina’ genome. The whole-genome sequence was targeted for alignment using the Bowtie 2 tool (Langmead and Salzberg, 2012) with default parameters. The consensus sequence of each cultivar was extracted from Binary Alignment Map (BAM) file using Geneious 11.1.5 software (Biomatters Ltd, Auckland, NZ). A target region of 69,179 bp in the ‘Regina’ genome, spanning the *PavDAM* genes (1,500 bp upstream of *PavDAM1* start codon to 1,500 bp downstream of *PavDAM6* stop codon), was analyzed in all the cultivars. Visual inspection was carried out to search for putative structural mutations. The full-length amino acid sequence of the six *DAM* genes from the 13 aligned sweet cherry cultivars was deduced and compared. The comparison was carried out by multiple amino acid sequence alignment using the ClustalW algorithm implemented in Geneious 11.1.5 software (Biomatters Ltd, Auckland, NZ). The percentage of identity between *DAM* genes of each cultivar was calculated as the percentage of identical amino acids between each pair of cultivars.

### Characterization of *PavDAM*s Structural Mutations in Low-Chilling and Early Blooming Cultivars

To confirm the presence of the putative structural mutations detected in ‘Cristobalina’ *PavDAM*s by *in silico* sequence comparison, primers flanking these regions were designed (PavD1UM: *PavDAM1* Upstream Mutation; PavD4/5M: *PavDAM4* and *-5* mutation). These PCR primers were designed in conserved regions observed in multiple cultivar alignments of these genes. PCR analyses using primer combinations PavD1UMr-PavD1UMf and PavD4/5Mr-PavD4/5Mf (Supplementary Table 1) were initially carried out in 14 cultivars (Table 1). The cultivars analyzed were those for which genome sequences were available (‘Ambrunés’, ‘Brooks’, ‘Cristobalina’, ‘Ferrovia’, ‘Hedelfingen’, ‘Lambert’, ‘Napoleon’, ‘Rainier’, ‘Regina’, ‘Sam’, ‘Satonishiki’, ‘Sue’, ‘Summit’, and ‘Vic’). PCR analysis was carried out as described in Cachi and Wünsch (2014) using the following program: 4 min at 94°C; 35 cycles of 45 s at 94°C, 45 s 59°C, and 2 min at 72°C; and a final step of 7 min at 72°C. PCR products were analyzed by agarose gel electrophoresis in 1.7% TBE and stained with GelRed® Nucleic Acid Stain (Biotium, CA, USA).

To characterize the genomic mutations identified in ‘Cristobalina’ *PavDAM*s, Sanger sequencing of PCR products (D1Sf-D1Sr and PavD4/5Mr-PavD4/5Mf; Supplementary Table 1) was carried out using ‘Cristobalina’ and ‘Regina’ DNA. PCR reactions were performed as described above. PCR products were purified and sequenced by STAB VIDA (Lisbon, Portugal). Sequencing of PCR products of each cultivar was repeated at least twice with each forward and reverse primer. All sequences were trimmed to eliminate low-quality nucleotides, and sequences from each cultivar were aligned to construct the consensus sequence of each cultivar (‘Cristobalina’ and ‘Regina’). These consensus sequences were then aligned for comparison. All sequences visualizing, editing, and alignments, as well as primers design, were carried out using Geneious 11.1.5 (Biomatters Ltd, Auckland, NZ).

Plant cis-acting regulatory DNA elements were searched upstream of ‘Regina’ *PavDAM1*, in the region covering the large deletion in ‘Cristobalina’. This search was performed using the PLACE database (https://www.dna.affrc.go.jp/PLACE/?action=newplace; Higo et al., 1999). ‘Regina’ published genome sequence was used as the template. Additionally, to compare *PavDAM*s expression in ‘Cristobalina’ and ‘Regina’, RNAseq data from both cultivars (Vimont et al., 2019) were aligned to the ‘Regina’ genome sequence (upstream of *PavDAM1, PavDAM4*, and *-5*) and on the Sanger sequencing for both cultivars. This analysis was carried out using HISAT2 (Kim et al., 2012).

### Validation of DNA-Markers of *PavDAM*s Structural Mutations (PavD1UM and PavD4/5M)

PavD1UM and PavD4/5M genotyping was carried out by PCR using primers PavD1UMf-PavD1UMr and PavD4/5Mf-PavD4/5Mr as described above (Supplementary Table 1). PavD1UM and PavD4/5M markers were validated in F_2_ population B×C2, which is expected to segregate for these markers because the parental genotype (‘BC8’) is heterozygous for both markers (Table 1). Description and mean bloom dates over 4 years (2015–2018) of B×C2 have been previously published (Calle et al., 2020). QTL haplotypes for major bloom time QTL on LG1 (*qP-BT1.1*^*m*^) of this population were also published in the same work. QTL haplotypes obtained then were compared with marker genotypes observed in this work. Deviation of marker segregation from expected Mendelian segregation in this population was evaluated by *Chi-square goodness-of-fit* (χ^2^). Statistical analysis was done using SPSS statistics v21.0.0 software (IMB, IL, USA) and R v3.4.1 (R Core Team, 2017). The two markers, PavD1UM and PavD4/5M, were also assayed in a diverse set of sweet cherry cultivars and accessions (Table 1).

## Results

### Mining, Annotation, and Structural Analyses of Candidate Genes in Major Bloom Time QTL (*qP-BT1.1^*m*^*)

Functional analysis in sweet cherry Chromosome 1 region (Chr1:49,296,241-49,622,837) of ‘Regina’ sweet cherry genome was carried out to identify candidate genes for bloom time and chilling requirement in sweet cherry. This genomic region spans major bloom time QTL *qP-BT1.1*^*m*^. In total, 47 predicted genes (Supplementary Table 2) were retrieved. Predicted amino acid sequences of seven of these genes (14.9%) resulted in BLAST hits in the NCBI gene database with uncharacterized proteins, while another six (12.8%) had no significant similarity with any other sequence (Supplementary Table 2). The rest of the predicted genes (34 genes; 72.3%), revealed hits with proteins involved in different pathways. Most relevant finding was eight contiguous genes, localized close to the QTL cofactor marker, which are sequentially annotated as PAV01_g0075081, PAV01_g0075091, PAV01_g0075101, PAV01_g0075111, PAV01_g0075121, PAV01_g0075131, PAV01_g0075141, and PAV01_g0075151 (Supplementary Table 2). Blastx, revealed these genes match MADS-box proteins, with percentages of similarity ranging from 86 to 100% (Supplementary Table 2). Due to their genetic similarity with type II SVP subclass of MADS-box proteins sequences, these eight sequences may correspond to *DAM* genes in sweet cherry (*PavDAM*), and they are therefore strong candidate genes for chilling requirement and bloom time regulation in this QTL region.

**Figure 1:**
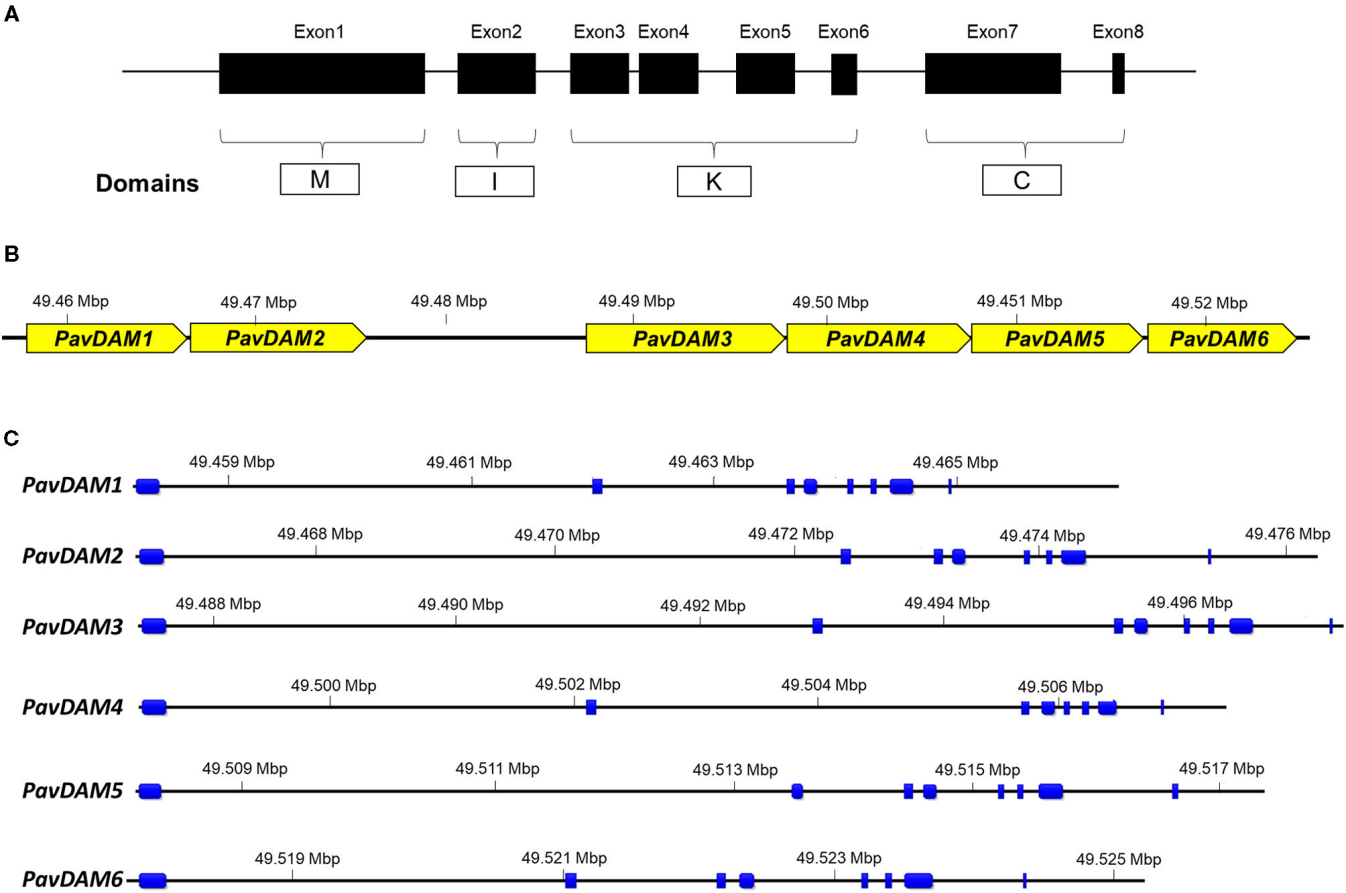
Characterization of sweet cherry *PavDAM* genes. **(A)** Schematic overview of the intron-exon structure of MADS-box genes and M, I, K, and C domains. **(B)** Diagram of size and position of *PavDAM* genes in chromosome 1 of the sweet cherry genome (Le Dantec et al., 2020). **(C)** Distribution of exons (blue boxes) and introns in the six *PavDAM* genes in ‘Regina’ sweet cherry genome.

Sequence inspection of these eight candidate genes revealed flaws in the automatic annotation of the initial gene models when compared to peach gene models. Besides, the expected structure of MADS-box domains was not complete. Only two predicted proteins (PAV1_g0075081 and PAV1_g0075151) contained domains MADS (M), Intervening (I), Keratin-like (K), and C-terminal (C), which are characteristics of type II MADS-box genes (Figure 1A). In another predicted gene (PAV01_g0075091), exon 3 was not annotated, and in PAV01_g0075121, two additional exons before the M domain were present. Similarly, PAV01_g0075101 and PAV01_g0075111 were automatically annotated as two different MADS-box, although domain structure revealed that both sequences were two separated fragments of the same MADS-box protein. The same was observed for PAV01_g0075131 and PAV01_g0075141 sequences, which correspond to the same MADS-box gene but had been automatically annotated as two different gene sequences.

The corrected annotation of the retrieved sequences revealed six MADS-box genes instead of the eight automatically predicted in the ‘Regina’ genome. The alignment of transcriptome data confirmed these coding structures and allowed an extensive curation of the UTR regions. Curated data can be accessed in Supplementary Table 3. Six *DAM* genes have also been previously reported in peach, Japanese apricot, and European plum in the syntenic genomic region. Thus, the six MADS-box sequences were identified as PAV1_g0075081, PAV1_g0075091, PAV1_g0075101, PAV1_g0075121, PAV1_g0075131, and PAV1_g0075151 in the ‘Regina’ genome, were named *PavDAM1* to *-6*, respectively (Figure 1). These genes are tandemly located in the ‘Regina’ genome (Chr01_49457863:49524699 bp) with a larger gap (11,433 bp) between *PavDAM2* and *-3* (Figure 1B). Gene structure analysis of the six genes revealed an identical structure of eight exons and seven introns in each gene, as well as, the conserved M, I, K, and C domains (Figure 1C). Genomic gene length ranged from 7,672 (*PavDAM6*) to 10,438 bp (*PavDAM3*), whereas the predicted genes coding regions ranged from 667 (*PavDAM4*) to 730 (*PavDAM5*) bp. Variable sizes were observed in the six introns of each gene, while exon sizes were highly conserved (Figure 1C).

### Phylogenetic Analysis of *PavDAM*s

**Figure 2:**
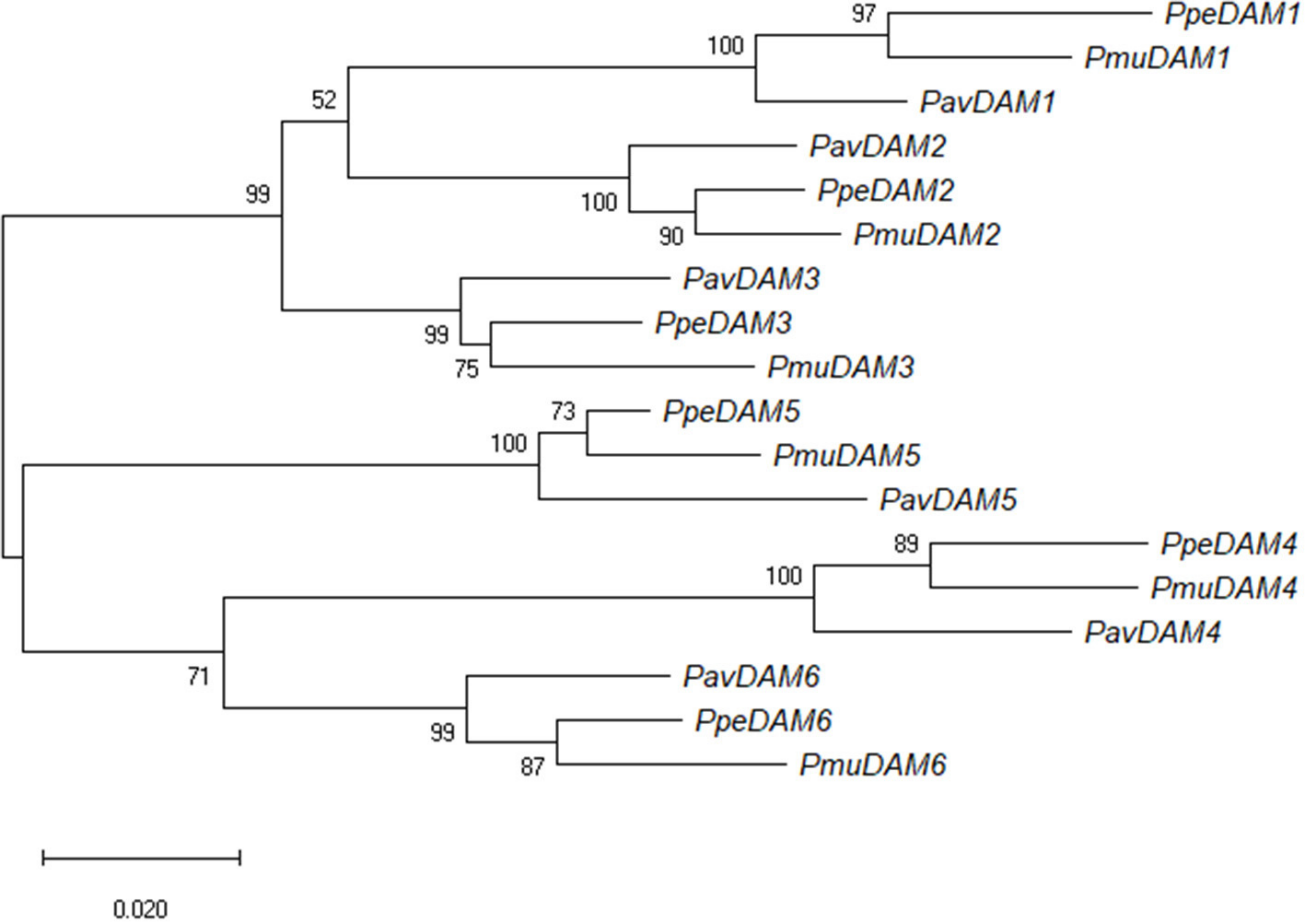
Maximum likelihood phylogenetic tree of nucleotide *DAM* sequences of sweet cherry (*PavDAM1, PavDAM2, PavDAM3, PavDAM4, PavDAM5*, and *PavDAM6*; Bielenberg et al., 2008) and its orthologs in Japanese apricot (*PmuDAM1, PmuDAM2, PmuDAM3, PmuDAM4, PmuDAM5*, and *PmuDAM6*; Sasaki et al., 2011) and peach (*PpeDAM1, PpeDAM2, PpeDAM3, PpeDAM4, PpeDAM5*, and *PpeDAM6*). The numbers at branch nodes indicate the percentage of bootstrap support at 1,000 replicates.

A phylogenetic analysis of peach, Japanese apricot (Bielenberg et al., 2008; Sasaki et al., 2011), and sweet cherry (this work) *DAM* genes was carried out using the maximum likelihood of the gene coding sequences (Figure 2). *DAM* genes orthologs (*DAM1* to *DAM6*) of the three species clustered together with a high bootstrap value (99; Figure 2). Within these sub-clades, in all cases, peach and Japanese apricot *DAM* genes were phylogenetically closer to each other than to sweet cherry *DAM* genes (Figure 2). Additionally, two major clades of *DAM* orthologs were observed, one includes *DAM1*, -*2*, and -*3*; and the other includes *DAM4*, -*5*, and -*6*, suggesting a common ancestor for each of them (Figure 2). Within these clades, *DAM1* and -*2* were closer to each other than to *DAM3*, and -*4* and *-6* were closer to each other than to *DAM5*.

### Intraspecific Variation of *PavDAM*s Sequences in Cultivars With Large Phenotypic Variation

The ‘Regina’ genome was used as a reference to map the genome sequence reads of 13 sweet cherry cultivars with variable chilling requirements and bloom times (Table 1). Of these cultivars, ‘Cristobalina’ shows extra early blooming while the rest show midseason to late flowering (Table 1). From this sequence mapping, the *PavDAM* genes consensus sequences of each cultivar were obtained. From these sequences, the complete amino acid sequence of each of the six *PavDAM* genes of each cultivar was predicted (Supplementary Figure 1). Comparison of *PavDAM* amino acid sequences amongst the different cultivars revealed a high degree of conservation (Supplementary Figure 1; Supplementary Tables 4, 5). The exon-intron structure was conserved in the six genes in all the cultivars. Also, the similarity between cultivars for the six *PavDAM* amino acid sequences was very high (98.8 to 100% identity; Supplementary Table 4). ‘Cristobalina’ was the cultivar with lower similarity to the rest (98.8–99.0%; Supplementary Table 4), while the remaining cultivars had higher similarities (99.7–100%). Complete amino acid identity (100% similarity) was observed for *PavDAM* sequences of ‘Ambrunés’ and ‘Summit’; ‘Vic’ and ‘Brooks’; and ‘Regina’, ‘Sam’, and ‘Sue’ (Supplementary Table 4).

Alignment of the *PavDAM* amino acid sequences of all the cultivars (Supplementary Figure 1) revealed 24 amino acid substitutions (Supplementary Figure 1; Supplementary Table 5). Of these, 20 were unique to specific cultivars, and the remaining four were common to various cultivars. ‘Cristobalina’ was the cultivar with the largest number of unique amino acid substitutions (14; Supplementary Figure 1; Supplementary Table 5). ‘Ferrovia’, ‘Lambert’, ‘Hedelfingen’, ‘Satonishiki’, and ‘Rainier’ showed 1–2 unique amino acid substitutions (Supplementary Table 5). *PavDAM1* and *PavDAM4* presented the largest number of polymorphisms (Supplementary Figure 1; Supplementary Table 5). Unique amino acid substitutions were found on all domains, with a large number found on domain C. Only ‘Cristobalina’ presented a substitution in the M domain (*PavDAM2* and -*5*).

Visual inspection of cultivars sequence reads mapping to the ‘Regina’ genome revealed two genomic regions where no sequence reads from ‘Cristobalina’ were mapped. These regions are located upstream of *PavDAM1*, and between *PavDAM4* and *-5* coding regions, spanning ~700 and 400 bp respectively (Supplementary Figures 2, 3). These regions seemed to contain putative structural mutations in the ‘Cristobalina’ genome.

### Characterization of *PavDAM*s Structural Mutations in Low-Chilling and Early Blooming Cultivars

**Figure 3:**
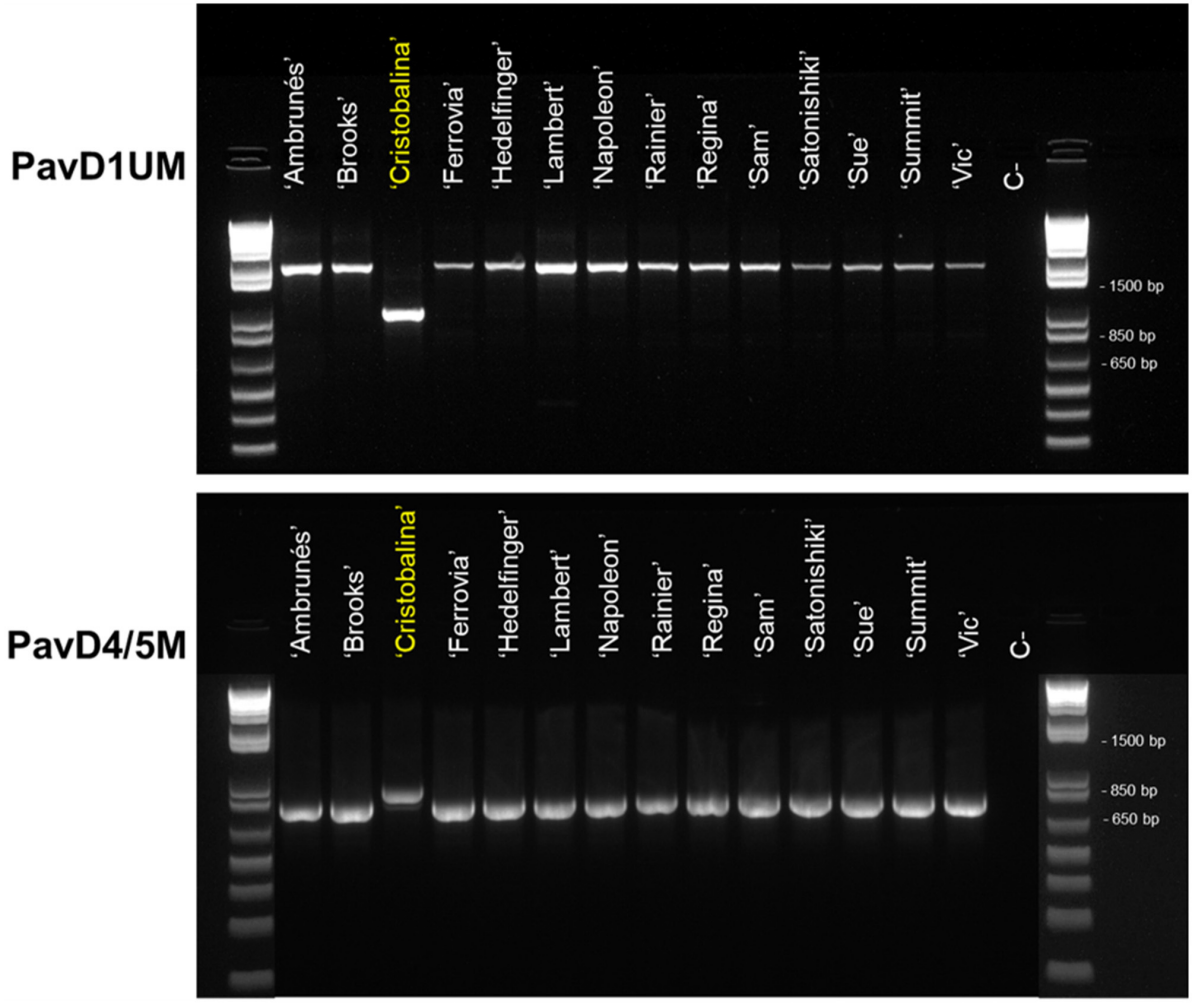
PCR analysis of *PavDAM1* and *PavDAM4* and *-5* structural mutations (PavD1UM and PavD4/5M, respectively) with primers PavD1UMf-PavD1UMr and PavD4/5Mf-PavD4/5Mr in 14 sweet cherry cultivars. C-: Negative control.

To investigate putative mutations in ‘Cristobalina’ *PavDAM*s (Supplementary Figures 2, 3), PCR primers flanking these regions (PavD1UM and PavD4/5M) were designed. These markers were used to analyze 13 cultivars with sequences available and ‘Regina’ (Table 1). For the PavD1UM marker, a fragment of the same size as in ‘Regina’ (~1,600 bp) was amplified in all the sweet cherry cultivars, except in ‘Cristobalina’, in which a shorter fragment (~900 bp) was obtained (Figure 3). The amplification of a smaller fragment in ‘Cristobalina’ supports the presence of a putative deletion of ~700 bp upstream of ‘Cristobalina’ *PavDAM1*. For the PavD4/5M marker, a fragment of 850 bp was amplified only in ‘Cristobalina’, whereas the remaining cultivars, including ‘Regina’, presented a 750 bp fragment (Figure 3). This result supports the presence of a putative insertion in the ‘Cristobalina’ genome, found between *PavDAM4* and *-5* coding regions.

**Figure 4:**
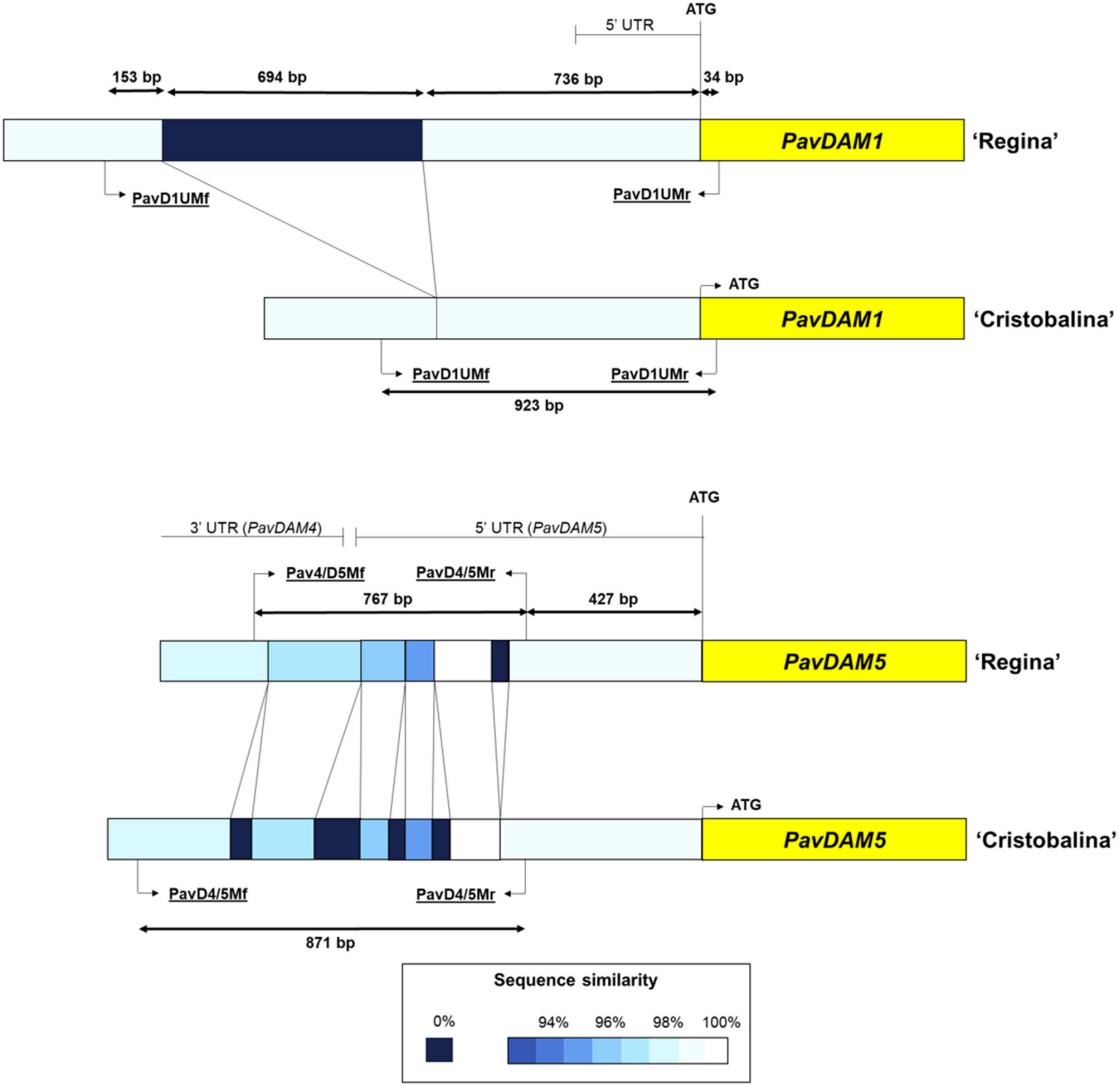
Schematic overview of *PavDAM1* and *PavDAM4* and *-5* structural mutations (PavD1UM and PavD4/5M, respectively) in ‘Cristobalina’ and ‘Regina’. PCR primers positions are shown (underlined text). The percentage of similarity between sequences is shown in different colors.

To confirm these mutations, these genomic regions were Sanger sequenced from PCR fragments using ‘Cristobalina’ and ‘Regina’ genomic DNA. The obtained sequences were compared (Supplementary Figures 4, 5), revealing a deletion of 694 bp in the ‘Cristobalina’ genome, 736 bp upstream of *PavDAM1* start codon of ‘Regina’ genome (Figure 4; Supplementary Figure 4). The rest of the sequence compared was highly similar except for a few SNPs (Supplementary Figure 4). For the *PavDAM4* and *-5* region, sequence comparison between ‘Cristobalina’ and ‘Regina’ revealed various polymorphisms (Figure 4; Supplementary Figure 5). These included four short insertions (21, 22, 30, and 46 bp) and one short deletion (18 bp) in ‘Cristobalina’; and 41 SNPs between both cultivars (Figure 4; Supplementary Figure 5).

Analysis of cis-acting regulatory sites in the ‘Regina’ sequence upstream of *PavDAM1*, which is absent in ‘Cristobalina’, revealed the presence of 60 unique sites (Supplementary Table 6). These include motifs like ARFAT, MYC, CArG, site II, TATA box, and WUSATAg that are associated with dormancy, bloom, flower development, and hormone regulation, among others (Supplementary Table 6; Supplementary Figure 4). Additionally, RNAseq data (Vimont et al., 2019) analysis in this genome region in both cultivars, ‘Regina’ and ‘Cristobalina’, revealed the alignment of short reads in ‘Regina’, but not in ‘Cristobalina’. The level of expression and the number of these reads were high enough to identify a putative non-coding gene that is expressed in ‘Regina’, but not in ‘Cristobalina’. On the other side, analysis of the highly variable region between *PavDAM4* and -*5* in ‘Cristobalina’ and ‘Regina’, revealed this region spans part of contiguous *PavDAM4* 3′UTR and *PavDAM5* 5′UTR (Figure 4; Supplementary Figure 5), with INDELs located in both UTRs. Splice junction coverage of RNAseq data in this region, in the two cultivars, revealed differences in splicing variants between ‘Regina’ and ‘Cristobalina’ for *PavDAM5*.

### Validation of DNA-Markers of *PavDAM*s Structural Mutations (PavD1UM and PavD4/5M)

**Figure 5:**
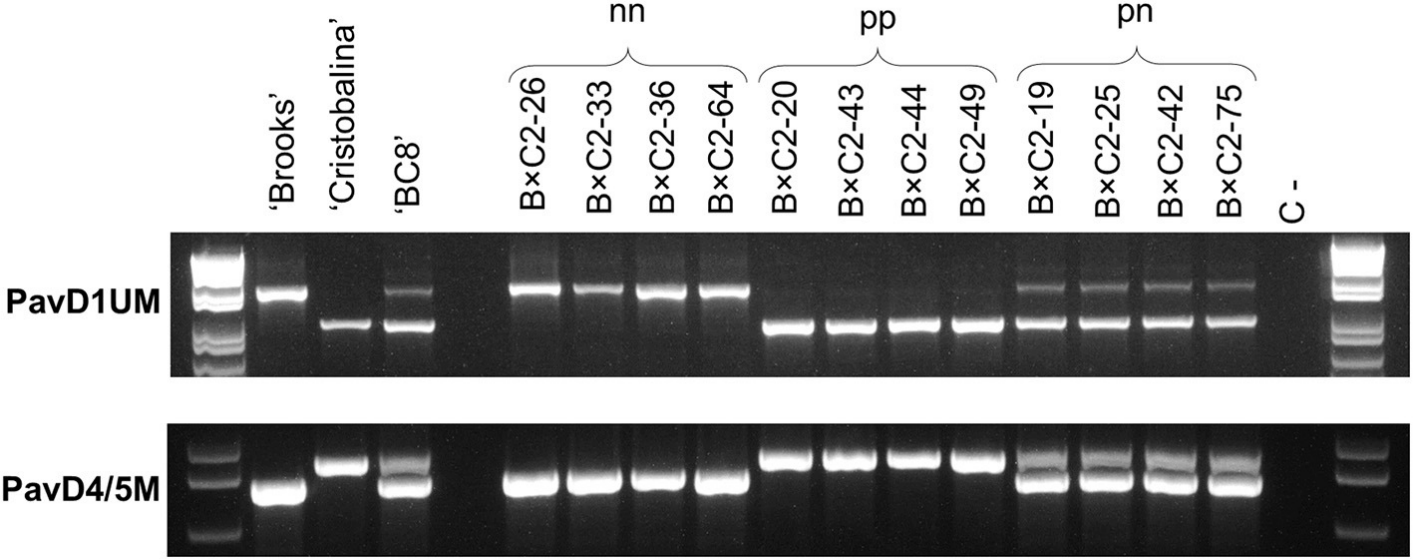
PCR analysis of *PavDAM1* and *PavDAM4* and *-5* structural mutations (PavD1UM and PavD4/5M, respectively) in B×C2 population with primers PavD1UMf-PavD1UMr and PavD4/5Mf-PavD4/5Mr, respectively. Four individuals from each segregating class are shown. Population B×C2 parental genotype (‘BC8’), and ‘BC8’ parental genotypes (‘Brooks’ and ‘Cristobalina’) are also shown. C-: Negative control.

PavD1UM and PavD4/5M analysis in F_2_ population B×C2 revealed three segregating classes for both markers. The same individuals were in the same segregating classes for both markers, confirming both markers are linked. For both markers the segregating classes were: homozygous like ‘Regina’ and ancestor ‘Brooks’ (genotype *nn*), heterozygous like the parental cultivar ‘BC8’ (*pn*), and homozygous like ‘Cristobalina’ (*pp*; Figure 5). For marker PavD1UM, these genotypes correspond to PCR fragments of ~1600, 950/1600, and 900 bp, respectively. In the case of PavD4/5M, the corresponding PCR genotypes are 750, 750/850, and 850 bps for *nn, pn*, and *pp*, respectively. The estimated exact expected sizes of these PCR fragments are 1638 and 944 bp for PavD1UM, and 766 and 867 bp for PavD4/5M. Segregation of the three classes occurred in the proportion 24:33:4 (*pp*:*pn*:*nn*), which significantly differs from the expected 1:2:1 ratio (χ^2^ = 1.87; Supplementary Table 7).

PavD1UM and PavD4/5M genotypes identified herein, and QTL *qP-BT1.1*^*m*^ genotypes previously reported for the same population B×C2 were compared (Supplementary Table 7). The comparison revealed that individuals with QTL haplotypes *cc, ac*, and *aa* (Calle et al., 2020) were the same as those belonging to PavD1UM and PavD4/5M segregating classes *pp, pn*, and *nn*, respectively (Supplementary Table 7). This result confirms that the mutations in *PavDAM*s in ‘Cristobalina’ show complete correlation with the QTL haplotypes associated with bloom date (Calle et al., 2020). Specifically, QTL haplotype *cc* is associated with earlier blooming than *ac*, and both correspond to extra-early and intermediate blooming phenotypes, respectively (Supplementary Table 7; Calle et al., 2020). Therefore, results herein confirm that PavD1UM and PavD4/5M markers are valid for identifying different bloom time QTL haplotypes, and therefore for identifying earlier and later blooming phenotypes from these plant materials. Additionally, PavD1UM and PavD4/5M markers genotyping allowed identifying the genotype of 14 recombinant individuals for this QTL in B×C2. From the 14 recombinants, three individuals corresponded to genotype *pp* and 11 to *pn* (Supplementary Table 7). Estimation of mean bloom date of each segregating class for both markers confirmed a significant difference of 7 days in mean bloom time between individuals of classes *pp* and *pn* (*p* < 0.001; *Student's T-*test; Supplementary Table 7). This is the same difference observed for QTL *qP-BT1.1*^*m*^ haplotypes in the same family. No phenotype data for *nn* individuals are available to estimate the phenotypic value of this segregating class (Supplementary Table 7).

Markers validation in a sweet cherry cultivar collection (Table 1) showed that only the low chilling and extra-early bloom time cultivars, ‘Temprana de Sot’ and ‘Royal Lee’ showed the same genotype as ‘Cristobalina’. These cultivars were homozygous for the early bloom allele (*pp*) for both markers (900 bp for PavD1UM, and 850 bp for PavD4/5M; Table 1). Additionally, the two local Spanish cultivars ‘De Mango Largo’ (midseason bloom) and ‘Son Perot’ (early bloom) were heterozygous (*pn*) for both markers PavD1UM (950/1600) and PavD4/5M (750/850) (Table 1). As described above, the selection ‘BC8’ (‘Brooks’ × ‘Cristobalina’), which shows early bloom time is also heterozygous for the markers. The rest of the cultivars, which show early to late bloom time, were homozygous (*nn*), and hence they had the same genotype as Regina (Table 1).

## Discussion

### Annotation, Structural, and Phylogenetic Analysis of Candidate Genes (*PavDAM*s) in Major Bloom Time QTL

In this study, six MADS-box genes, *PavDAM*, were identified in sweet cherry major bloom time QTL, *qP-BT1.1*^*m*^. This bloom time QTL was previously detected in populations derived from the low chilling and extra-early blooming cultivar ‘Cristobalina’ (Calle et al., 2020). This chromosome 1 genome region is determinant in the genetic control of chilling requirements and bloom time in sweet cherry, as other QTLs for these traits were also previously reported on the same location in sweet cherry populations from different genetic backgrounds (Dirlewanger et al., 2012; Castède et al., 2014). Six tandemly arranged MICK^c^-type MADS-box, denoted *DAM* genes, have been previously identified in the syntenic region of chromosome 1 in the almond, peach, Japanese apricot, and European plum genomes (Xu et al., 2014; Wells et al., 2015; Quesada-Traver et al., 2020). In sweet cherry, *PavDAM* genes have been recently cloned and sequenced from flower bud RNA, and their cDNA and predicted amino acid sequences have been reported (Wang et al., 2020). In the present work, the genomic sequence and structure of these genes were characterized and annotated from the sweet cherry genome sequence of Regina cv (Le Dantec et al., 2020).

The amino acid sequence of *PavDAMs* recently predicted in ‘Royal Lee’ and ‘Hongdeng’ cultivars (Wang et al., 2020) is highly similar to that reported in this work for the ‘Regina’ genome (99.0 and 99.9%, respectively). The sequence of each *PavDAM* reported in this work includes the four characteristic domains of MIKC type II MADS-box, as reported earlier in peach, Japanese apricot, or plum (Jiménez et al., 2009; Xu et al., 2014; Quesada-Traver et al., 2020). Furthermore, we observed in this study, that each *PavDAM* comprises eight exons making the genomic structure of the six genes very similar to that of the *DAM* genes previously reported in other *Prunus* species, namely peach, Japanese apricot, European plum (Jiménez et al., 2009; Sasaki et al., 2011; Quesada-Traver et al., 2020). Thus, the six MADS-box (*PavDAM*) genes identified within the major bloom time QTL in this work, as expected, are solid candidate genes for chilling requirement and bloom time regulation in sweet cherry.

Like in earlier works (Rothkegel et al., 2017; Wang et al., 2020), phylogenetic analysis in this work revealed that *PavDAM*s are orthologs to the peach and Japanese apricot corresponding *DAM* genes. Within each *DAM* gene clade, peach and Japanese apricot genes appeared phylogenetically closer to each other than to sweet cherry genes, reflecting the species phylogeny. Peach and Japanese apricot, belong to *Amygdalus* and *Prunus* subgenus, respectively, phylogenetically closer to each other than to the sweet cherry subgenus (*Cerasus*) (Potter et al., 2007). The detection of six clades of *DAM* ortholog groups indicates that *DAM* diversification occurred before *Prunus* speciation. Additionally, the six *DAM* genes may be paralogs (outparalogs), as earlier duplication events may have led to the six tandemly arranged genes (Koonin, 2005). As suggested before (Jiménez et al., 2009; Li et al., 2009), posterior subfunctionalization and/or neofunctionalization may have resulted in their actual function. The clustering of the *DAM* orthologs in two major clades, namely *DAM1*, -*2*, and -*3*; and *DAM4*, -*5*, and -*6*, as previously observed (Prudencio et al., 2018; Balogh et al., 2019; Quesada-Traver et al., 2020; Wang et al., 2020), agrees with previous transcriptomic studies of *DAM* genes in peach and Japanese apricot, in which two different expression patterns have been observed for the two groups of genes. *DAM1*, -*2*, and -*3* have a maximum expression during bud set, while *DAM4*, -*5*, and -*6* show maximum expression when chilling requirement are satisfied (Falavigna et al., 2019).

### Intraspecific Variation of *PavDAM*s Sequences in Cultivars With Large Phenotypic Variation

*PavDAM*s predicted amino acid sequences of 13 sweet cherry cultivars revealed a high degree of similarity despite their different genetic backgrounds and contrasting phenotypes (Table 1). Also, these sequences are highly similar to those previously reported (‘Royal Lee’ and ‘Hongdeng’; Wang et al., 2020). This high degree of conservation may indicate that *PavDAM*s proteins’ phenotypic effect may be more dependent on expression regulation than on protein structure. Most amino acid differences among the cultivars studied were found in the same positions, confirming also the presence of highly variable amino acids. However, no correlation of these amino acid polymorphisms could be associated with the chilling requirement and/or bloom time of these cultivars. Nevertheless, it cannot be discarded that these amino acid substitutions may be associated with phenotypic differences. Single amino acid substitutions in MADS-box genes in *Arabidopsis* have been associated with the loss of function leading to early flowering phenotypes (Hartmann et al., 2000; Méndez-Vigo et al., 2013).

‘Cristobalina’ *PavDAM* genes showed the lowest similarity with the rest of the cultivars and accumulated the largest number of unique amino acid substitutions. ‘Cristobalina’ was the only cultivar that has a unique amino substitution in the M domain of *PavDAM2* and *-5*, whereas more substitutions were observed in the C domain in all *DAM*s. It has been reported that the M domain is the most conserved of all MADS-box domains; and that the C domain, which is related to protein complex formation and transcriptomic activation, is the most variable (Honma and Goto, 2001; Kaufmann et al., 2005). The differences observed between the *PavDAM* genes coding sequences of ‘Cristobalina’ and the other cultivars analyzed may be associated with the phenotypic differences in chilling requirements and/or bloom time. ‘Cristobalina’ has lower chilling requirements and earlier blooming than the other cultivars analyzed (Table 1; Tabuenca, 1983; Alburquerque et al., 2008; Calle et al., 2020). It has also been observed that ‘Cristobalina’ enters endodormancy later and fulfills its chilling requirements before medium to late bloom time cultivars (Fadón et al., 2018). The genetic differences may be due to a different genetic origin and adaptation to different eco-geographic regions. ‘Cristobalina’ is a local Spanish cultivar from the Mediterranean region and is genetically well-differentiated from the rest of the cultivars analyzed (Wünsch and Hormaza, 2002; Martínez-Royo and Wünsch, 2014).

It was also observed that, despite the large variability exhibited by ‘Cristobalina’ *PavDAM*s predicted amino acid sequences, these are identical to those reported for ‘Royal Lee’ (Wang et al., 2020). These two cultivars seemed unrelated, as there is no proof of a relationship between them. ‘Royal Lee’ is also a low-chill cultivar, which derives from a breeding program in California (Zaiger's Inc Genetics; US patent N° 12417), while ‘Cristobalina’ is a local Spanish landrace. The chilling requirements of ‘Royal Lee’ (Wang et al., 2020) are also similar to those of ‘Cristobalina’ (approx. 400 chilling hours; Tabuenca, 1983). A possible explanation for this unexpected genetic and phenotypic similarity is that ‘Cristobalina’ is an ancestor of ‘Royal Lee’. In fact, the contribution of a low-chilling cultivar of unknown origin is described in the ‘Royal Lee’ pedigree (US patent N° 12417). In any case, the similarities observed reinforce the hypothesis that the genetic differences identified in ‘Cristobalina’ *PavDAMs* may be the cause of low chilling and extra early blooming.

### *PavDAM*s Structural Mutations in Low-Chilling and Early Blooming Cultivars

Greater variation upstream of ‘Cristobalina’ *PavDAM1* and between *PavDAM4* and *-5* was also identified in this work. Specifically, a 694 bp deletion, 736 bp upstream of the *PavDAM1* coding sequence, and a highly polymorphic region, which includes various INDELs, in the UTRs of *PavDAM4* and *-5*, were detected. These mutations were detected in ‘Cristobalina’ by sequence reads mapping to the ‘Regina’ genome sequence (Le Dantec et al., 2020), and confirmed by Sanger sequencing of PCR fragments spanning the mutations. PCR markers (PavD1UM, PavD4/5M) were designed to detect these mutations and to validate their association with low chilling and early blooming in an F_2_ segregating population and in a cultivar collection.

Analysis of PCR fragments from PavD1UM and PavD4/5M markers in the only available segregating population for these mutations (F_2_ population B×C2) revealed a complete correlation with the linkage group 1 bloom time QTL *qP-BT1.1*^*m*^ segregating classes (Calle et al., 2020). These results indicate a correlation between the presence of the mutation and earlier blooming (7 days) in homozygous genotypes (*pp*). Furthermore, analyses of the markers in a sweet cherry cultivar collection with genotypes with large phenotypic differences for chilling requirements and bloom time also revealed an association of the mutations in homozygosis (*pp*), with low chilling and extra-early blooming. The other cultivars for which PavD1UM and PavD4/5M mutations were identified were other local Spanish cultivars (‘Temprana de Sot’, ‘Son Perot’, and ‘De Mango Largo’) and the bred cultivar ‘Royal Lee’. The presence in other local Spanish cultivars confirms that the putative origin of this *PavDAM* haplotype is the southern European Mediterranean region. The presence of these mutations also in ‘Royal Lee’ confirms that the *PavDAM* genotype is the same in ‘Cristobalina’ and ‘Royal Lee’, as discussed above for the *PavDAMs* protein sequences. It also confirms the correlation of this genotype with low chilling and extra-early blooming. The result also reinforces the hypothesis that ‘Cristobalina’ may be part of the pedigree of ‘Royal Lee’.

In two other local Spanish cultivars (‘Son Perot’ and ‘De Mango Largo’), the ‘Cristobalina’ *PavDAM*s mutations were found in heterozygosity (*pn* genotypes). These cultivars have early and medium bloom date phenotypes and their chilling requirements are not known. Similarly, in other individuals from ‘Cristobalina’-derived populations, which are heterozygous for these mutations (data not shown), different bloom time phenotypes have been observed (Calle et al., 2020), but none of them show such early-blooming as those in which the mutations are in homozygosity (*pp*, like in ‘Cristobalina’). In fact, in the B×C2 population, the heterozygous individuals (*np*) are not as early blooming as the homozygous ones (*pp*) (see Calle et al., 2020). The phenotypic effect associated with these mutations is more evident in those individuals homozygous for the mutations probably due to the additive effect of each *PavDAM* haplotype. The rest of the cultivars analyzed with the *PavDAM* markers, PavD1UM and PavD4/5M, are homozygous for the absence of the mutation (*nn*). Among these, there are cultivars of medium to high chilling requirements from early to late blooming. This result also indicates that not all early blooming cultivars in sweet cherry have the same mutation as ‘Cristobalina’, and therefore, that there are additional sources of early blooming in sweet cherry. But only, the extra-early cultivars analyzed do have the described *PavDAM* mutations and protein sequences. Therefore, the markers developed in this work correlate with earlier bloom time (and probably low chilling) and are useful for the selection of this trait from ‘Cristobalina’ and likely from ‘Royal Lee’ too.

‘Cristobalina’ *PavDAM* genotype has revealed several unique polymorphisms in its predicted protein sequences and large structural mutations upstream of *PavDAM1* and in contiguous *PavDAM4* and -*5* UTR sequences. These structural mutations were shown to correlate with extra-early blooming. Although further research is needed, it cannot be discarded that these mutations may be the cause of low-chilling and extra-early blooming in this cultivar. Protein variability in relevant conserved regions of *PavDAM*s may be altering protein function in this genotype due to variation in oligomerization in conserved regions (Lai et al., 2019). Alternatively, the structural mutations observed may result in differential gene expression of *PavDAM*s in this cultivar. Differential expression of *PavDAM*s in ‘Cristobalina’ (and ‘Royal Lee’) has been observed in transcriptomic analyses during dormancy when compared with high-chilling cultivars (Vimont et al., 2019; Supplementary Figure 6; Wang et al., 2020). Most evident differences have been observed for *PavDAM1*,−*4*, and *-5* (Vimont et al., 2019; Supplementary Figure 6; Wang et al., 2020). Specifically, the expression of *PavDAM1* of ‘Royal Lee’ has been shown to decrease much earlier than in the high chilling cultivar ‘Hongdeng’ (Wang et al., 2020). A similar result has been observed for ‘Cristobalina’ compared with ‘Regina’ (Vimont et al., 2019). For *PavDAM4* and -*5*, large differences have been observed between ‘Cristobalina’ and ‘Regina’, especially for *PavDAM4* in which much lower expression was observed in ‘Cristobalina’ (Vimont et al., 2019).

The deletion upstream of *PavDAM1* in ‘Cristobalina’ results in the absence of potentially relevant cis-acting binding sites. *DAM* genes have been observed to be regulated by proteins related to the response of environmental signals and the cold response pathway that can bind to *DAM* promoters (Zhao et al., 2015). This is the case of *C-Repeat Binding Factors* (*CBF*), which have been reported in some *Rosaceae* species binding *DAM* promoter and to regulate these genes expression in apple, Japanese apricot, and pear (Mimida et al., 2015; Saito et al., 2015; Wisniewski et al., 2015; Zhao et al., 2018). Besides, CArG box motif is the target region of MADS-box transcription factor, but also their own regulation (Zhu and Perry, 2005; Gregis et al., 2013). More recently, it was shown that the site II motif was recognized by the *PpeTCP20* transcription factor, down-regulating the expression of *DAM5* and *-6* in peach (Wang et al., 2020). Motifs CArG and site II, among others, are missing in the deleted region upstream of *PavDAM1* in ‘Cristobalina’. The putative involvement of any missing cis-acting elements in *PavDAMs* expression would be compatible with the differential expression of ‘Cristobalina’ (and ‘Royal Lee’) *PavDAM*s (Vimont et al., 2019; Supplementary Figure 6; Wang et al., 2020). Additionally, in the same genomic region, the expression of a non-coding gene in ‘Regina’ seems truncated in ‘Cristobalina’. Blast analysis indicates the existence of this ncRNA in peach but it has not been detected in other organisms, and therefore, could be *Prunus*-specific. As no other relevant information could be obtained from this non-coding gene, further analyses are required to confirm the potential involvement of this gene in the ‘Cristobalina’ phenotype.

The variation observed in *PavDAM4* and *-5* UTRs between ‘Regina’ and ‘Cristobalina’ may also have implications in *PavDAM*s expression, and/or in *PavDAM*s transcripts variability. UTRs can influence gene expression in plants (Srivastava et al., 2018). Noticeably, we observed a predominance of specific splicing variants in each cultivar, ‘Cristobalina’ seems to have a shorter 5′UTR than ‘Regina’. UTR length could influence expression levels as well as play a role in various post-transcriptional processes (Mignone et al., 2002), which can result in *PavDAM5* differential transcription, translation, and/or function. Additionally, these mutations in *PavDAM4* and *-5* could also affect the expression of the other *PavDAM*s, as previously observed in the *EVG* peach mutant (with four deleted *DAM* genes), where the two intact genes (*DAM1* and *-2*) were not expressed (Bielenberg et al., 2008). It is, therefore, necessary to further investigate these mutations, to identify their potential effect in *PavDAM*s differential transcription, and their correlation with the contrasting phenotypes.

### PavD1UM and PavD4/5M, Markers for Breeding for Early Blooming and Low Chilling Requirements

‘Cristobalina’ is a relevant cultivar for breeding, due to self-compatibility, low chilling requirements, and extra-early bloom time. The PavD1UM and PavD4/5M markers, developed here, are a useful tool for sweet cherry breeding of low chilling requirement and early bloom time from ‘Cristobalina’ using marker-assisted selection. These markers revealed a complete correlation with the haplotypes of bloom time QTL (*qP-BT1.1*^*m*^), which accounts for up to 50.1% of the phenotypic variation in ‘Cristobalina’ derived populations (Calle et al., 2020). The large correlation between QTL and marker genotypes, as well as the large amount of phenotypic variation explained by this QTL, makes these markers useful tools for discriminating individuals with lower chilling requirement and earlier blooming, which will be associated with the presence of the mutations in homozygosity or heterozygosity. Earlier blooming is expected to be associated with the presence of the deletion in homozygosity and later blooming and higher chilling requirement will be associated with the absence of the mutations. Besides, the identification of these mutations also in the low chill cultivar ‘Royal Lee’, indicate these markers may also useful for selection from cultivars from different genetic backgrounds other than ‘Cristobalina’.

In the present study, the analysis of candidate genes in a previously reported main bloom time QTL in sweet cherry has allowed for the characterization and annotation of *PavDAM* genes in the species. This work thus confirms *PavDAM*s as candidate genes for bloom time regulation in sweet cherry. Protein sequence polymorphisms and structural mutations identified in *PavDAM*s of low-chilling and extra-early blooming cv. Cristobalina were shown to correlate with earlier blooming in a segregating population and with extra-early blooming in a diverse set of cultivars. These results indicate that the ‘Cristobalina’ *PavDAM* genotype may be the genetic causal variation of the phenotypic differences exhibited by ‘Cristobalina’, low chilling requirement, and extra-early bloom time, although further research is needed to confirm this hypothesis. PCR DNA-markers based on these structural mutations (useful for the selection of early blooming from this plan material) were designed and validated.

## Data Availability Statement

The data presented in this study are deposited in the CITA online repository (https://citarea.cita-aragon.es/citarea/) accesion number https://citarea.cita-aragon.es/citarea/handle/10532/5397. This is an updated model based on the raw sequences deposited at genbank DDBJ (PRJDB6734).

## Author Contributions

AC carried out experimental work, data analysis and interpretation, and manuscript writing and revision. JG participated in the bioinformatics analyses, experimental design, data interpretation, and manuscript writing and editing. LL provided reference genome and participated in manuscript revision. AW participated in experimental design, data analysis supervision, manuscript writing and editing, and work coordination. All authors contributed to the article and approved the submitted version.

## Conflict of Interest

The authors declare that the research was conducted in the absence of any commercial or financial relationships that could be construed as a potential conflict of interest.

## Abbreviations

*AGL24*: *AGAMOUS*-*LIKE 24*
BAM: Binary Alignment Map
*CBF*: *C-Repeat Binding Factors*
cv: cultivar
DAM: Dormancy associated MADS-box
DDBJ: DNA Data Bank of Japan
EVG: ever-growing peach mutant
GFF: General File Format
IGV: Integrative Genomics Viewers
LG: linkage group
MCL: Maximum Composite Likelihood
NCBI: National Center for Biotechnology Information
PavD1UM: *PavDAM1* Upstream Mutation
PavD4/5: *PavDAM4* and -5 mutation
PCR: polymerase chain reaction
QTL: quantitative trait locus
*SVP*: *SHORT VEGETATIVE PHASE*
TBE: Tris-Borate-EDTA buffer.
