# Supplementary material for: Identification and Characterization of DAMs Mutations Associated With Early Blooming in Sweet Cherry, and Validation of DNA-Based Markers for Selection
Source: Front Plant Sci. 2021 Jul 8;12:621491. doi: 10.3389/fpls.2021.621491 (PMC8295754; doi:10.3389/fpls.2021.621491)

Supplementary Material

**Supplementary Table 1** PCR primers used.

| **Name** |  | **Sequence (5’ to 3’)** |
| --- | --- | --- |
| PavD1UMf | Forward | CCTCGATCGCTCTGACCATC |
| PavD1UMr | Reverse | GCTGGCTAGCTGTTCGAGAA |
| PavD4/5Mf | Forward | GCGTCCCTAATTTCCTTGGT |
| PAvD4/5Mr | Reverse | GGACAGATGCTCTTGACGTT |
| D1Sf | Forward | CCATCTCTCTCCCATCTCGT |
| D1Sr | Reverse | TGCAGGCAAGTTGTCAATCT |

**Supplementary Table 2** Predicted genes of the ‘Regina’ sweet cherry genome (Le Dantec et al., 2020) in Chromosome 1: 49,296,241- 49,622,837. Sequence description of protein sequences in NCBI database and statistical significance of highest matches from BLAST. Candidate *DAM* genes are shown in bold.

| **Predicted gene** | **Description** | **Max score** | **Total score** | **Query cover** | **E value** | **Identity** |
| --- | --- | --- | --- | --- | --- | --- |
| PAV01_g0074801 | rho GTPase-activating protein REN1 isoform X1 [*Prunus yedoensis* var. *nudiflora*] | 1524 | 1524 | 99% | 0 | 91% |
| PAV01_g0074811 | mitochondrial uncoupling protein 5-like [*Prunus avium*] | 647 | 647 | 99% | 0 | 100% |
| PAV01_g0074831 | probable pectinesterase/pectinesterase inhibitor 7 [*Prunus avium*] | 1162 | 1162 | 99% | 0 | 100% |
| PAV01_g0074851 | probable pectinesterase/pectinesterase inhibitor 20 [*Prunus avium*] | 1162 | 1162 | 99% | 0 | 99% |
| PAV01_g0074861 | cytochrome b561 and DOMON domain-containing protein At5g47530-like [*Prunus* *avium*] | 715 | 715 | 99% | 0 | 99% |
| PAV01_g0074871 | uncharacterized protein LOC110751910 [*Prunus avium*] | 327 | 327 | 99% | 8.00E-110 | 100% |
| PAV01_g0074881 | aspartyl protease AED3 [*Prunus avium*] | 763 | 763 | 99% | 0 | 90% |
| PAV01_g0074891 | universal stress protein PHOS34 [*Prunus avium*] | 276 | 276 | 99% | 1.00E-92 | 79% |
| PAV01_g0074901 | eukaryotic translation initiation factor 3 subunit C-like isoform X1 [*Prunus avium*] | 1941 | 1941 | 99% | 0 | 99% |
| PAV01_g0074911 | ubiquitin carboxyl-terminal hydrolase 16-like [*Prunus avium*] | 2228 | 2228 | 99% | 0 | 98% |
| PAV01_g0074921 | AT-hook motif nuclear-localized protein 17 [*Prunus avium*] | 387 | 387 | 99% | 3.00E-134 | 100% |
| PAV01_g0074931 | hypothetical protein KK1_049021 [*Cajanus cajan*] | 52.4 | 52.4 | 57% | 3.00E-07 | 60% |
| PAV01_g0074941 | AP-4 complex subunit mu [*Prunus avium*] | 921 | 921 | 99% | 0 | 100% |
| PAV01_g0074951 | putative methyltransferase At1g22800 isoform X2 [*Prunus avium*] | 705 | 705 | 99% | 0 | 100% |
| PAV01_g0074961 | cytochrome b561 and DOMON domain-containing protein At5g47530-like [*Prunus avium*] | 715 | 715 | 99% | 0 | 99% |
| PAV01_g0074971 | uncharacterized protein LOC110765872 [k] | 197 | 197 | 71% | 4.00E-61 | 79% |
| PAV01_g0074981 | No significant similarity found | - | - | - | - | - |
| PAV01_g0074991 | hypothetical protein PRUPE_4G104600 [*Prunus persica*] | 97.4 | 97.4 | 97% | 1.00E-24 | 79% |
| PAV01_g0075001 | small heat shock protein chloroplastic [*Prunus yedoensis* var. *nudiflora*] | 314 | 314 | 99% | 1.00E-106 | 84% |
| PAV01_g0075011 | putative DNA helicase [*Rosa chinensis*] | 102 | 153 | 58% | 6.00E-23 | 77% |
| PAV01_g0075021 | beta carbonic anhydrase 5, chloroplastic-like isoform X2 [*Prunus avium*] | 648 | 648 | 99% | 0 | 99% |
| PAV01_g0075031 | transcription factor bHLH30-like [*Prunus avium*] | 491 | 491 | 99% | 1.00E-175 | 100% |
| PAV01_g0075041 | uncharacterized protein LOC110751898 [*Prunus avium*] | 488 | 808 | 99% | 1.00E-170 | 83% |
| PAV01_g0075051 | uncharacterized protein Pyn_34535 [*Prunus yedoensis* var. *nudiflora*] | 662 | 662 | 99% | 0 | 100% |
|  |  |  |  |  |  |  |
| **Supplementary Table 2** Continued. | | | | | | |
| PAV01_g0075061 | No significant similarity found | - | - | - | - | - |
| PAV01_g0075071 | No significant similarity found | - | - | - | - | - |
| **PAV01_g0075081** | **MADS-box protein JOINTLESS-like isoform X5 [*Prunus avium*]** | **472** | **472** | **99%** | **2.00E-168** | **100%** |
| **PAV01_g0075091** | **MADS-box protein JOINTLESS-like [*Prunus avium*]** | **424** | **424** | **99%** | **2.00E-149** | **91%** |
| **PAV01_g0075101** | **MADS1 [*Prunus avium*]** | **230** | **230** | **99%** | **1.00E-74** | **100%** |
| **PAV01_g0075111** | **MADS1 [*Prunus avium*]** | **246** | **246** | **99%** | **1.00E-80** | **99%** |
| **PAV01_g0075121** | **MADS-box protein JOINTLESS-like isoform X4 [*Prunus avium*]** | **446** | **717** | **76%** | **1.00E-153** | **99%** |
| **PAV01_g0075131** | **MADS-box protein JOINTLESS-like isoform X7 [*Prunus avium*]** | **129** | **129** | **51%** | **2.00E-34** | **86%** |
| **PAV01_g0075141** | **MADS-box protein JOINTLESS-like isoform X6 [*Prunus avium*]** | **317** | **317** | **93%** | **4.00E-108** | **100%** |
| **PAV01_g0075151** | **MADS-box protein JOINTLESS-like isoform X5 [*Prunus avium*]** | **479** | **479** | **99%** | **9.00E-171** | **100%** |
| PAV01_g0075161 | No significant similarity found | - | - | - | - | - |
| PAV01_g0075171 | O-fucosyltransferase 31 [*Rosa chinensis*] | 930 | 930 | 99% | 0 | 87% |
| PAV01_g0075181 | mechanosensitive ion channel protein 3, chloroplastic-like isoform X1 [*Prunus avium*] | 1457 | 1457 | 99% | 0 | 99% |
| PAV01_g0075191 | No significant similarity found | - | - | - | - | - |
| PAV01_g0075201 | kinesin-like protein KIN-4C isoform X1 [*Prunus avium*] | 2633 | 2633 | 99% | 0 | 100% |
| PAV01_g0075211 | No significant similarity found | - | - | - | - | - |
| PAV01_g0075231 | kinesin-like protein KIN-4C [*Prunus avium*] | 1126 | 1126 | 99% | 0 | 99% |
| PAV01_g0075241 | argininosuccinate lyase, chloroplastic [*Prunus avium*] | 1077 | 1077 | 99% | 0 | 100% |
| PAV01_g0075251 | uncharacterized protein LOC110772580 isoform X3 [*Prunus avium*] | 344 | 344 | 99% | 8.00E-120 | 100% |
| PAV01_g0075261 | protein NETWORKED 2A-like [*Prunus avium*] | 2024 | 2024 | 99% | 0 | 100% |
| PAV01_g0075271 | pentatricopeptide repeat-containing protein At2g37230 [*Prunus avium*] | 1542 | 1542 | 96% | 0 | 99% |
| PAV01_g0075281 | uncharacterized protein LOC110772590 isoform X2 [*Prunus avium*] | 557 | 557 | 99% | 0 | 100% |
| PAV01_g0075291 | uncharacterized protein LOC110772590 isoform X1 [*Prunus avium*] | 698 | 698 | 95% | 0 | 100% |

**Supplementary Table 3** GFF file of correct annotation of *PavDAM* genes in the ‘Regina’ sweet cherry genome (Le Dantec et al., 2020). Automatic and Manual (EuGene_Manual) intro-exon prediction is indicated.

| ### |  |  |  |  |  |  |  |  |
| --- | --- | --- | --- | --- | --- | --- | --- | --- |
| PAV01_REGINA | EuGene_Manual | Gene | 49457863 | 49465728 | . | + | . | ID=gene:PavDAM1;Name=PavDAM1 |
| PAV01_REGINA | EuGene_Manual | mRNA | 49457863 | 49465728 | . | + | . | ID=mRNA:PavDAM1;Name=PavDAM1;Parent=gene:PavDAM1 |
| PAV01_REGINA | EuGene_Manual | Exon | 49457863 | 49457952 | . | + | . | ID=exon:PavDAM1.utr0;Parent=mRNA:PavDAM1;Ontology_term=SO:0000204 |
| PAV01_REGINA | EuGene_Manual | Exón | 49458233 | 49458426 | . | + | . | ID=exon:PavDAM1.1;Parent=mRNA:PavDAM1;Ontology_term=SO:0000200 |
| PAV01_REGINA | EuGene_Manual | Exón | 49462000 | 49462078 | . | + | 1 | ID=exon:PavDAM1.2;Parent=mRNA:PavDAM1;Ontology_term=SO:0000004 |
| PAV01_REGINA | EuGene_Manual | Exón | 49463597 | 49463658 | . | + | 0 | ID=exon:PavDAM1.3;Parent=mRNA:PavDAM1;Ontology_term=SO:0000004 |
| PAV01_REGINA | EuGene_Manual | Exón | 49463744 | 49463843 | . | + | 1 | ID=exon:PavDAM1.4;Parent=mRNA:PavDAM1;Ontology_term=SO:0000004 |
| PAV01_REGINA | EuGene_Manual | Exón | 49464102 | 49464143 | . | + | 0 | ID=exon:PavDAM1.5;Parent=mRNA:PavDAM1;Ontology_term=SO:0000004 |
| PAV01_REGINA | EuGene_Manual | Exón | 49464295 | 49464336 | . | + | 0 | ID=exon:PavDAM1.6;Parent=mRNA:PavDAM1;Ontology_term=SO:0000004 |
| PAV01_REGINA | EuGene_Manual | Exón | 49464452 | 49464636 | . | + | 0 | ID=exon:PavDAM1.7;Parent=mRNA:PavDAM1;Ontology_term=SO:0000004 |
| PAV01_REGINA | EuGene_Manual | Exón | 49464942 | 49465728 | . | + | 1 | ID=exon:PavDAM1.8;Parent=mRNA:PavDAM1;Ontology_term=SO:0000202 |
| PAV01_REGINA | EuGene_Manual | five_prime_UTR | 49457863 | 49457952 | . | + | . | ID=five_prime_UTR:PavDAM1.0;Parent=mRNA:PavDAM1;Ontology_term=SO:0000204;est_cons=100.0;est_incons=0.0 |
| PAV01_REGINA | EuGene_Manual | five_prime_UTR | 49458233 | 49458238 | . | + | . | ID=five_prime_UTR:PavDAM1.2;Parent=mRNA:PavDAM1;Ontology_term=SO:0000204;est_cons=100.0;est_incons=0.0 |
| PAV01_REGINA | EuGene_Manual | CDS | 49458239 | 49458426 | . | + | 0 | ID=CDS:PavDAM1.1;Parent=mRNA:PavDAM1;Ontology_term=SO:0000196;est_cons=100.0;est_incons=0.0 |
| PAV01_REGINA | EuGene_Manual | CDS | 49462000 | 49462078 | . | + | 1 | ID=CDS:PavDAM1.2;Parent=mRNA:PavDAM1;Ontology_term=SO:0000004;est_cons=100.0;est_incons=0.0 |
| PAV01_REGINA | EuGene_Manual | CDS | 49463597 | 49463658 | . | + | 0 | ID=CDS:PavDAM1.3;Parent=mRNA:PavDAM1;Ontology_term=SO:0000004;est_cons=100.0;est_incons=0.0 |
| PAV01_REGINA | EuGene_Manual | CDS | 49463744 | 49463843 | . | + | 1 | ID=CDS:PavDAM1.4;Parent=mRNA:PavDAM1;Ontology_term=SO:0000004;est_cons=100.0;est_incons=0.0 |
| PAV01_REGINA | EuGene_Manual | CDS | 49464102 | 49464143 | . | + | 0 | ID=CDS:PavDAM1.5;Parent=mRNA:PavDAM1;Ontology_term=SO:0000004;est_cons=100.0;est_incons=0.0 |
| PAV01_REGINA | EuGene_Manual | CDS | 49464295 | 49464336 | . | + | 0 | ID=CDS:PavDAM1.6;Parent=mRNA:PavDAM1;Ontology_term=SO:0000004;est_cons=100.0;est_incons=0.0 |
| PAV01_REGINA | EuGene_Manual | CDS | 49464452 | 49464636 | . | + | 0 | ID=CDS:PavDAM1.7;Parent=mRNA:PavDAM1;Ontology_term=SO:0000004;est_cons=100.0;est_incons=0.0 |
| PAV01_REGINA | EuGene_Manual | CDS | 49464942 | 49464951 | . | + | 1 | ID=CDS:PavDAM1.8;Parent=mRNA:PavDAM1;Ontology_term=SO:0000197;est_cons=100.0;est_incons=0.0 |
| PAV01_REGINA | EuGene_Manual | three_prime_UTR | 49464952 | 49465728 | . | + | . | ID=three_prime_UTR:PavDAM1.18;Parent=mRNA:PavDAM1;Ontology_term=SO:0000205;est_cons=100.0;est_incons=0.0 |
| ### |  |  |  |  |  |  |  |  |
| PAV01_REGINA | EuGene_Manual | gene | 49466174 | 49475605 | . | + | . | ID=gene:PavDAM2;Name=PavDAM2 |
| PAV01_REGINA | EuGene_Manual | mRNA | 49466174 | 49475605 | . | + | . | ID=mRNA:PavDAM2;Name=PavDAM2;Parent=gene:PavDAM2 |
| PAV01_REGINA | EuGene_Manual | exon | 49466174 | 49466254 | . | + | . | ID=exon:PavDAM2.utr0;Parent=mRNA:PavDAM2;Ontology_term=SO:0000204 |
| PAV01_REGINA | EuGene_Manual | exon | 49466560 | 49466755 | . | + | . | ID=exon:PavDAM2.1;Parent=mRNA:PavDAM2;Ontology_term=SO:0000200 |
| **Supplementary Table 3** Continued. | | | | | | | | |
| PAV01_REGINA | EuGene_Manual | Exón | 49472342 | 49472420 | . | + | 1 | ID=exon:PavDAM2.2;Parent=mRNA:PavDAM2;Ontology_term=SO:0000004 |
| PAV01_REGINA | EuGene_Manual | Exón | 49473111 | 49473173 | . | + | 0 | ID=exon:PavDAM2.3;Parent=mRNA:PavDAM2;Ontology_term=SO:0000004 |
| PAV01_REGINA | EuGene_Manual | Exón | 49473262 | 49473357 | . | + | 0 | ID=exon:PavDAM2.4;Parent=mRNA:PavDAM2;Ontology_term=SO:0000004 |
| PAV01_REGINA | EuGene_Manual | Exón | 49473853 | 49473894 | . | + | 0 | ID=exon:PavDAM2.5;Parent=mRNA:PavDAM2;Ontology_term=SO:0000004 |
| PAV01_REGINA | EuGene_Manual | Exón | 49474036 | 49474077 | . | + | 0 | ID=exon:PavDAM2.6;Parent=mRNA:PavDAM2;Ontology_term=SO:0000004 |
| PAV01_REGINA | EuGene_Manual | Exón | 49474161 | 49474354 | . | + | 0 | ID=exon:PavDAM2.7;Parent=mRNA:PavDAM2;Ontology_term=SO:0000004 |
| PAV01_REGINA | EuGene_Manual | Exón | 49475376 | 49475605 | . | + | 1 | ID=exon:PavDAM2.8;Parent=mRNA:PavDAM2;Ontology_term=SO:0000202 |
| PAV01_REGINA | EuGene_Manual | five_prime_UTR | 49466174 | 49466254 | . | + | . | ID=five_prime_UTR:PavDAM2.0;Parent=mRNA:PavDAM2;Ontology_term=SO:0000204;est_cons=100.0;est_incons=0.0 |
| PAV01_REGINA | EuGene_Manual | five_prime_UTR | 49466560 | 49466564 | . | + | . | ID=five_prime_UTR:PavDAM2.2;Parent=mRNA:PavDAM2;Ontology_term=SO:0000204;est_cons=100.0;est_incons=0.0 |
| PAV01_REGINA | EuGene_Manual | CDS | 49466565 | 49466755 | . | + | 0 | ID=CDS:PavDAM2.1;Parent=mRNA:PavDAM2;Ontology_term=SO:0000196;est_cons=100.0;est_incons=0.0 |
| PAV01_REGINA | EuGene_Manual | CDS | 49472342 | 49472420 | . | + | 1 | ID=CDS:PavDAM2.2;Parent=mRNA:PavDAM2;Ontology_term=SO:0000004;est_cons=69.6;est_incons=0.0 |
| PAV01_REGINA | EuGene_Manual | CDS | 49473111 | 49473171 | . | + | 0 | ID=CDS:PavDAM2.3;Parent=mRNA:PavDAM2;Ontology_term=SO:0000004;est_cons=0.0;est_incons=0.0 |
| PAV01_REGINA | EuGene_Manual | CDS | 49473262 | 49473357 | . | + | 0 | ID=CDS:PavDAM2.4;Parent=mRNA:PavDAM2;Ontology_term=SO:0000004;est_cons=0.0;est_incons=0.0 |
| PAV01_REGINA | EuGene_Manual | CDS | 49473853 | 49473894 | . | + | 0 | ID=CDS:PavDAM2.5;Parent=mRNA:PavDAM2;Ontology_term=SO:0000004;est_cons=0.0;est_incons=0.0 |
| PAV01_REGINA | EuGene_Manual | CDS | 49474036 | 49474077 | . | + | 0 | ID=CDS:PavDAM2.6;Parent=mRNA:PavDAM2;Ontology_term=SO:0000004;est_cons=0.0;est_incons=0.0 |
| PAV01_REGINA | EuGene_Manual | CDS | 49474161 | 49474354 | . | + | 0 | ID=CDS:PavDAM2.7;Parent=mRNA:PavDAM2;Ontology_term=SO:0000004;est_cons=80.9;est_incons=0.0 |
| PAV01_REGINA | EuGene_Manual | CDS | 49475376 | 49475385 | . | + | 1 | ID=CDS:PavDAM2.8;Parent=mRNA:PavDAM2;Ontology_term=SO:0000197;est_cons=100.0;est_incons=0.0 |
| PAV01_REGINA | EuGene_Manual | three_prime_UTR | 49475386 | 49475605 | . | + | . | ID=three_prime_UTR:PavDAM2.16;Parent=mRNA:PavDAM2;Ontology_term=SO:0000205;est_cons=100.0;est_incons=0.0 |
| ### |  |  |  |  |  |  |  |  |
| PAV01_REGINA | EuGene_Manual | Gene | 49487038 | 49497476 | . | + | . | ID=gene:PavDAM3;Name=PavDAM3 |
| PAV01_REGINA | EuGene_Manual | mRNA | 49487038 | 49497476 | . | + | . | ID=mRNA:PavDAM3;Name=PavDAM3;Parent=gene:PavDAM3 |
| PAV01_REGINA | EuGene_Manual | Exon | 49487038 | 49487138 | . | + | . | ID=exon:PavDAM3.0;Parent=mRNA:PavDAM3;Ontology_term=SO:0000200 |
| PAV01_REGINA | EuGene_Manual | Exón | 49487416 | 49487610 | . | + | . | ID=exon:PavDAM3.1;Parent=mRNA:PavDAM3;Ontology_term=SO:0000200 |
| PAV01_REGINA | EuGene_Manual | Exón | 49492939 | 49493017 | . | + | 1 | ID=exon:PavDAM3.2;Parent=mRNA:PavDAM3;Ontology_term=SO:0000004 |
| PAV01_REGINA | EuGene_Manual | Exón | 49495428 | 49495489 | . | + | 0 | ID=exon:PavDAM3.3;Parent=mRNA:PavDAM3;Ontology_term=SO:0000202 |
| PAV01_REGINA | EuGene_Manual | five_prime_UTR | 49487038 | 49487138 | . | + | . | ID=five_prime_UTR:PavDAM3.0;Parent=mRNA:PavDAM3;Ontology_term=SO:0000204;est_cons=100.0;est_incons=0.0 |
| PAV01_REGINA | EuGene_Manual | five_prime_UTR | 49487416 | 49487419 | . | + | . | ID=five_prime_UTR:PavDAM3.0;Parent=mRNA:PavDAM3;Ontology_term=SO:0000204;est_cons=100.0;est_incons=0.0 |
| PAV01_REGINA | EuGene_Manual | CDS | 49487420 | 49487610 | . | + | 0 | ID=CDS:PavDAM3.1;Parent=mRNA:PavDAM3;Ontology_term=SO:0000196;est_cons=100.0;est_incons=0.0 |
| PAV01_REGINA | EuGene_Manual | CDS | 49492939 | 49493017 | . | + | 1 | ID=CDS:PavDAM3.2;Parent=mRNA:PavDAM3;Ontology_term=SO:0000004;est_cons=100.0;est_incons=0.0 |
| PAV01_REGINA | EuGene_Manual | CDS | 49495428 | 49495489 | . | + | 0 | ID=CDS:PavDAM3.3;Parent=mRNA:PavDAM3;Ontology_term=SO:0000197;est_cons=100.0;est_incons=0.0 |
| **Supplementary Table 3** Continued. | | | | | | | | |
| PAV01_REGINA | EuGene_Manual | Exon | 49495591 | 49495690 | . | + | 1 | ID=exon:PavDAM3.4;Parent=mRNA:PavDAM3;Ontology_term=SO:0000200 |
| PAV01_REGINA | EuGene_Manual | Exón | 49496003 | 49496044 | . | + | 0 | ID=exon:PavDAM3.5;Parent=mRNA:PavDAM3;Ontology_term=SO:0000004 |
| PAV01_REGINA | EuGene_Manual | Exón | 49496199 | 49496240 | . | + | 0 | ID=exon:PavDAM3.6;Parent=mRNA:PavDAM3;Ontology_term=SO:0000004 |
| PAV01_REGINA | EuGene_Manual | Exón | 49496375 | 49496559 | . | + | 0 | ID=exon:PavDAM3.7;Parent=mRNA:PavDAM3;Ontology_term=SO:0000004 |
| PAV01_REGINA | EuGene_Manual | exon | 49497205 | 49497476 | . | + | 1 | ID=exon:PavDAM3.8;Parent=mRNA:PavDAM3;Ontology_term=SO:0000202 |
| PAV01_REGINA | EuGene_Manual | CDS | 49495591 | 49495690 | . | + | 1 | ID=CDS:PavDAM3.4;Parent=mRNA:PavDAM3;Ontology_term=SO:0000196;est_cons=100.0;est_incons=0.0 |
| PAV01_REGINA | EuGene_Manual | CDS | 49496003 | 49496044 | . | + | 0 | ID=CDS:PavDAM3.5;Parent=mRNA:PavDAM3;Ontology_term=SO:0000004;est_cons=100.0;est_incons=0.0 |
| PAV01_REGINA | EuGene_Manual | CDS | 49496199 | 49496240 | . | + | 0 | ID=CDS:PavDAM3.6;Parent=mRNA:PavDAM3;Ontology_term=SO:0000004;est_cons=100.0;est_incons=0.0 |
| PAV01_REGINA | EuGene_Manual | CDS | 49496375 | 49496559 | . | + | 0 | ID=CDS:PavDAM3.7;Parent=mRNA:PavDAM3;Ontology_term=SO:0000004;est_cons=100.0;est_incons=0.0 |
| PAV01_REGINA | EuGene_Manual | CDS | 49497205 | 49497214 | . | + | 1 | ID=CDS:PavDAM3.8;Parent=mRNA:PavDAM3;Ontology_term=SO:0000197;est_cons=100.0;est_incons=0.0 |
| PAV01_REGINA | EuGene_Manual | three_prime_UTR | 49497215 | 49497476 | . | + | . | ID=three_prime_UTR:PavDAM3.10;Parent=mRNA:PavDAM3;Ontology_term=SO:0000205;est_cons=100.0;est_incons=0.0 |
| ### |  |  |  |  |  |  |  |  |
| PAV01_REGINA | EuGene_Manual | gene | 49497528 | 49507115 | . | + | . | ID=gene:PavDAM4;Name=PavDAM4 |
| PAV01_REGINA | EuGene_Manual | mRNA | 49497528 | 49507115 | . | + | . | ID=mRNA:PavDAM4;Name=PavDAM4;Parent=gene:PavDAM4 |
| PAV01_REGINA | EuGene_Manual | exon | 49497528 | 49498153 | . | + | 0 | ID=exon:PavDAM4.0;Parent=mRNA:PavDAM4;Ontology_term=SO:0000004 |
| PAV01_REGINA | EuGene_Manual | exon | 49498451 | 49498645 | . | + | 0 | ID=exon:PavDAM4.1;Parent=mRNA:PavDAM4;Ontology_term=SO:0000004 |
| PAV01_REGINA | EuGene_Manual | exon | 49502110 | 49502185 | . | + | 1 | ID=exon:PavDAM4.2;Parent=mRNA:PavDAM4;Ontology_term=SO:0000004 |
| PAV01_REGINA | EuGene_Manual | exon | 49505693 | 49505754 | . | + | 0 | ID=exon:PavDAM4.3;Parent=mRNA:PavDAM4;Ontology_term=SO:0000004 |
| PAV01_REGINA | EuGene_Manual | exon | 49505857 | 49505956 | . | + | 1 | ID=exon:PavDAM4.4;Parent=mRNA:PavDAM4;Ontology_term=SO:0000004 |
| PAV01_REGINA | EuGene_Manual | exon | 49506042 | 49506083 | . | + | 0 | ID=exon:PavDAM4.5;Parent=mRNA:PavDAM4;Ontology_term=SO:0000004 |
| PAV01_REGINA | EuGene_Manual | exon | 49506193 | 49506243 | . | + | 0 | ID=exon:PavDAM4.6;Parent=mRNA:PavDAM4;Ontology_term=SO:0000004 |
| PAV01_REGINA | EuGene_Manual | exon | 49506326 | 49506468 | . | + | 0 | ID=exon:PavDAM4.7;Parent=mRNA:PavDAM4;Ontology_term=SO:0000004 |
| PAV01_REGINA | EuGene_Manual | exon | 49506852 | 49507115 | . | + | 1 | ID=exon:PavDAM4.8;Parent=mRNA:PavDAM4;Ontology_term=SO:0000202 |
| PAV01_REGINA | EuGene_Manual | five_prime_UTR | 49497528 | 49498153 | . | + | . | ID=five_prime_UTR:PavDAM4.0;Parent=mRNA:PavDAM4;Ontology_term=SO:0000204;est_cons=100.0;est_incons=0.0 |
| PAV01_REGINA | EuGene_Manual | CDS | 49498455 | 49498645 | . | + | 0 | ID=CDS:PavDAM4.1;Parent=mRNA:PavDAM4;Ontology_term=SO:0000004;est_cons=100.0;est_incons=0.0 |
| PAV01_REGINA | EuGene_Manual | CDS | 49502110 | 49502185 | . | + | 1 | ID=CDS:PavDAM4.2;Parent=mRNA:PavDAM4;Ontology_term=SO:0000004;est_cons=100.0;est_incons=0.0 |
| PAV01_REGINA | EuGene_Manual | CDS | 49505693 | 49505754 | . | + | 0 | ID=CDS:PavDAM4.3;Parent=mRNA:PavDAM4;Ontology_term=SO:0000004;est_cons=100.0;est_incons=0.0 |
| PAV01_REGINA | EuGene_Manual | CDS | 49505857 | 49505956 | . | + | 1 | ID=CDS:PavDAM4.4;Parent=mRNA:PavDAM4;Ontology_term=SO:0000004;est_cons=100.0;est_incons=0.0 |
| PAV01_REGINA | EuGene_Manual | CDS | 49506042 | 49506083 | . | + | 0 | ID=CDS:PavDAM4.5;Parent=mRNA:PavDAM4;Ontology_term=SO:0000004;est_cons=100.0;est_incons=0.0 |
| PAV01_REGINA | EuGene_Manual | CDS | 49506193 | 49506243 | . | + | 0 | ID=CDS:PavDAM4.6;Parent=mRNA:PavDAM4;Ontology_term=SO:0000004;est_cons=100.0;est_incons=0.0 |
| **Supplementary Table 3** Continued. | | | | | | | | |
| PAV01_REGINA | EuGene_Manual | CDS | 49506326 | 49506468 | . | + | 0 | ID=CDS:PavDAM4.7;Parent=mRNA:PavDAM4;Ontology_term=SO:0000004;est_cons=100.0;est_incons=0.0 |
| PAV01_REGINA | EuGene_Manual | CDS | 49506852 | 49506861 | . | + | 1 | ID=CDS:PavDAM4.8;Parent=mRNA:PavDAM4;Ontology_term=SO:0000197;est_cons=100.0;est_incons=0.0 |
| PAV01_REGINA | EuGene_Manual | three_prime_UTR | 49506862 | 49507115 | . | + | . | ID=three_prime_UTR:PavDAM4.20;Parent=mRNA:PavDAM4;Ontology_term=SO:0000205;est_cons=100.0;est_incons=0.0 |
| ### |  |  |  |  |  |  |  |  |
| PAV01_REGINA | EuGene_Manual | gene | 49507129 | 49516973 | . | + | . | ID=gene:PavDAM5;Name=PavDAM5 |
| PAV01_REGINA | EuGene_Manual | mRNA | 49507129 | 49516973 | . | + | . | ID=mRNA:PavDAM5;Name=PavDAM5;Parent=gene:PavDAM5 |
| PAV01_REGINA | EuGene_Manual | mRNA | 49507494 | 49516973 | . | + | . | ID=mRNA:PavDAM5;Name=PavDAM5;Parent=gene:PavDAM5 |
| PAV01_REGINA | EuGene_Manual | Exon | 49507129 | 49507171 | . | + | . | ID=exon:PavDAM5.utr22;Parent=mRNA:PavDAM5;Ontology_term=SO:0000205 |
| PAV01_REGINA | EuGene_Manual | five_prime_UTR | 49507129 | 49507171 | . | + | . | ID=five_prime_UTR:PavDAM5.22;Parent=mRNA:PavDAM5;Ontology_term=SO:0000205;est_cons=100.0;est_incons=0.0 |
| PAV01_REGINA | EuGene_Manual | Exón | 49507315 | 49507673 | . | + | . | ID=exon:PavDAM5.utr24;Parent=mRNA:PavDAM5;Ontology_term=SO:0000205 |
| PAV01_REGINA | EuGene_Manual | five_prime_UTR | 49507315 | 49507673 | . | + | . | ID=five_prime_UTR:PavDAM5.24;Parent=mRNA:PavDAM5;Ontology_term=SO:0000205;est_cons=100.0;est_incons=0.0 |
| PAV01_REGINA | EuGene_Manual | exon | 49507494 | 49507673 | . | + | . | ID=exon:PavDAM5.utr24;Parent=PavDAM5;Ontology_term=SO:0000205 |
| PAV01_REGINA | EuGene_Manual | five_prime_UTR | 49507494 | 49507673 | . | + | . | ID=five_prime_UTR:PavDAM5.24;Parent=PavDAM5;Ontology_term=SO:0000205;est_cons=100.0;est_incons=0.0 |
| PAV01_REGINA | EuGene_Manual | exon | 49507774 | 49507839 | . | + | . | ID=exon:PavDAM5.utr0;Parent=mRNA:PavDAM5,PavDAM5;Ontology_term=SO:0000204 |
| PAV01_REGINA | EuGene_Manual | five_prime_UTR | 49507774 | 49507839 | . | + | . | ID=five_prime_UTR:PavDAM5.0;Parent=mRNA:PavDAM5,PavDAM5;Ontology_term=SO:0000204;est_cons=100.0;est_incons=0.0 |
| PAV01_REGINA | EuGene_Manual | exon | 49508062 | 49508243 | . | + | . | ID=exon:PavDAM5.1;Parent=mRNA:PavDAM5,PavDAM5;Ontology_term=SO:0000200 |
| PAV01_REGINA | EuGene_Manual | five_prime_UTR | 49508062 | 49508064 | . | + | . | ID=five_prime_UTR:PavDAM5.2;Parent=mRNA:PavDAM5,PavDAM5;Ontology_term=SO:0000204;est_cons=100.0;est_incons=0.0 |
| PAV01_REGINA | EuGene_Manual | exon | 49513443 | 49513521 | . | + | 1 | ID=exon:PavDAM5.1;Parent=mRNA:PavDAM5,PavDAM5;Ontology_term=SO:0000200 |
| PAV01_REGINA | EuGene_Manual | exon | 49514370 | 49514431 | . | + | 0 | ID=exon:PavDAM5.2;Parent=mRNA:PavDAM5,PavDAM5;Ontology_term=SO:0000004 |
| PAV01_REGINA | EuGene_Manual | exon | 49514524 | 49514623 | . | + | 1 | ID=exon:PavDAM5.3;Parent=mRNA:PavDAM5,PavDAM5;Ontology_term=SO:0000004 |
| PAV01_REGINA | EuGene_Manual | exon | 49515144 | 49515185 | . | + | 0 | ID=exon:PavDAM5.4;Parent=mRNA:PavDAM5,PavDAM5;Ontology_term=SO:0000004 |
| PAV01_REGINA | EuGene_Manual | exon | 49515299 | 49515340 | . | + | 0 | ID=exon:PavDAM5.5;Parent=mRNA:PavDAM5,PavDAM5;Ontology_term=SO:0000004 |
| PAV01_REGINA | EuGene_Manual | exon | 49515475 | 49515668 | . | + | 0 | ID=exon:PavDAM5.6;Parent=mRNA:PavDAM5,PavDAM5;Ontology_term=SO:0000004 |
| PAV01_REGINA | EuGene_Manual | exon | 49516580 | 49516707 | . | + | 1 | ID=exon:PavDAM5.7;Parent=mRNA:PavDAM5,PavDAM5;Ontology_term=SO:0000202 |
| PAV01_REGINA | EuGene_Manual | exon | 49516797 | 49516973 | . | + | . | ID=exon:PavDAM5.utr16;Parent=mRNA:PavDAM5,PavDAM5;Ontology_term=SO:0000205 |
| PAV01_REGINA | EuGene_Manual | CDS | 49508065 | 49508243 | . | + | 0 | ID=CDS:PavDAM5.1;Parent=mRNA:PavDAM5,PavDAM5;Ontology_term=SO:0000196;est_cons=100.0;est_incons=0.0 |
| PAV01_REGINA | EuGene_Manual | CDS | 49513443 | 49513521 | . | + | 1 | ID=CDS:PavDAM5.2;Parent=mRNA:PavDAM5,PavDAM5;Ontology_term=SO:0000196;est_cons=100.0;est_incons=0.0 |
| PAV01_REGINA | EuGene_Manual | CDS | 49514370 | 49514431 | . | + | 0 | ID=CDS:PavDAM5.3;Parent=mRNA:PavDAM5,PavDAM5;Ontology_term=SO:0000004;est_cons=100.0;est_incons=0.0 |
| PAV01_REGINA | EuGene_Manual | CDS | 49514524 | 49514623 | . | + | 1 | ID=CDS:PavDAM5.4;Parent=mRNA:PavDAM5,PavDAM5;Ontology_term=SO:0000004;est_cons=100.0;est_incons=0.0 |
| PAV01_REGINA | EuGene_Manual | CDS | 49515144 | 49515185 | . | + | 0 | ID=CDS:PavDAM5.5;Parent=mRNA:PavDAM5,PavDAM5;Ontology_term=SO:0000004;est_cons=100.0;est_incons=0.0 |
| PAV01_REGINA | EuGene_Manual | CDS | 49515299 | 49515340 | . | + | 0 | ID=CDS:PavDAM5.6;Parent=mRNA:PavDAM5,PavDAM5;Ontology_term=SO:0000004;est_cons=100.0;est_incons=0.0 |
| **Supplementary Table 3** Continued. | | | | | | | | |
| PAV01_REGINA | EuGene_Manual | CDS | 49515475 | 49515668 | . | + | 0 | ID=CDS:PavDAM5.7;Parent=mRNA:PavDAM5,PavDAM5;Ontology_term=SO:0000004;est_cons=100.0;est_incons=0.0 |
| PAV01_REGINA | EuGene_Manual | CDS | 49516580 | 49516619 | . | + | 1 | ID=CDS:PavDAM5.8;Parent=mRNA:PavDAM5,PavDAM5;Ontology_term=SO:0000197;est_cons=100.0;est_incons=0.0 |
| PAV01_REGINA | EuGene_Manual | three_prime_UTR | 49516620 | 49516707 | . | + | . | ID=three_prime_UTR:PavDAM5.14;Parent=mRNA:PavDAM5,PavDAM5;Ontology_term=SO:0000205;est_cons=100.0;est_incons=0.0 |
| PAV01_REGINA | EuGene_Manual | three_prime_UTR | 49516797 | 49516973 | . | + | . | ID=three_prime_UTR:PavDAM5.16;Parent=mRNA:PavDAM5,PavDAM5;Ontology_term=SO:0000205;est_cons=100.0;est_incons=0.0 |
| ### |  |  |  |  |  |  |  |  |
| PAV01_REGINA | EuGene_Manual | gene | 49517027 | 49524699 | . | + | . | ID=gene:PavDAM6;Name=PavDAM6 |
| PAV01_REGINA | EuGene_Manual | mRNA | 49517027 | 49524699 | . | + | . | ID=mRNA:PavDAM6;Name=PavDAM6;Parent=gene:PavDAM6 |
| PAV01_REGINA | EuGene_Manual | exon | 49517027 | 49517614 | . | + | . | ID=exon:PavDAM6.utr0;Parent=mRNA:PavDAM6;Ontology_term=SO:0000204 |
| PAV01_REGINA | EuGene_Manual | exon | 49517878 | 49518072 | . | + | . | ID=exon:PavDAM6.1;Parent=mRNA:PavDAM6;Ontology_term=SO:0000200 |
| PAV01_REGINA | EuGene_Manual | exon | 49521025 | 49521103 | . | + | 1 | ID=exon:PavDAM6.2;Parent=mRNA:PavDAM6;Ontology_term=SO:0000004 |
| PAV01_REGINA | EuGene_Manual | exon | 49522143 | 49522204 | . | + | 0 | ID=exon:PavDAM6.3;Parent=mRNA:PavDAM6;Ontology_term=SO:0000004 |
| PAV01_REGINA | EuGene_Manual | exon | 49522307 | 49522406 | . | + | 1 | ID=exon:PavDAM6.4;Parent=mRNA:PavDAM6;Ontology_term=SO:0000004 |
| PAV01_REGINA | EuGene_Manual | exon | 49523208 | 49523249 | . | + | 0 | ID=exon:PavDAM6.5;Parent=mRNA:PavDAM6;Ontology_term=SO:0000004 |
| PAV01_REGINA | EuGene_Manual | exon | 49523384 | 49523425 | . | + | 0 | ID=exon:PavDAM6.6;Parent=mRNA:PavDAM6;Ontology_term=SO:0000004 |
| PAV01_REGINA | EuGene_Manual | exon | 49523526 | 49523722 | . | + | 0 | ID=exon:PavDAM6.7;Parent=mRNA:PavDAM6;Ontology_term=SO:0000004 |
| PAV01_REGINA | EuGene_Manual | exon | 49524409 | 49524699 | . | + | 1 | ID=exon:PavDAM6.8;Parent=mRNA:PavDAM6;Ontology_term=SO:0000202 |
| PAV01_REGINA | EuGene_Manual | five_prime_UTR | 49517027 | 49517614 | . | + | . | ID=five_prime_UTR:PavDAM6.0;Parent=mRNA:PavDAM6;Ontology_term=SO:0000204;est_cons=100.0;est_incons=0.0 |
| PAV01_REGINA | EuGene_Manual | five_prime_UTR | 49517878 | 49517881 | . | + | . | ID=five_prime_UTR:PavDAM6.2;Parent=mRNA:PavDAM6;Ontology_term=SO:0000204;est_cons=100.0;est_incons=0.0 |
| PAV01_REGINA | EuGene_Manual | CDS | 49517882 | 49518072 | . | + | 0 | ID=CDS:PavDAM6.1;Parent=mRNA:PavDAM6;Ontology_term=SO:0000196;est_cons=88.0;est_incons=0.0 |
| PAV01_REGINA | EuGene_Manual | CDS | 49521025 | 49521103 | . | + | 1 | ID=CDS:PavDAM6.2;Parent=mRNA:PavDAM6;Ontology_term=SO:0000004;est_cons=100.0;est_incons=0.0 |
| PAV01_REGINA | EuGene_Manual | CDS | 49522143 | 49522204 | . | + | 0 | ID=CDS:PavDAM6.3;Parent=mRNA:PavDAM6;Ontology_term=SO:0000004;est_cons=100.0;est_incons=0.0 |
| PAV01_REGINA | EuGene_Manual | CDS | 49522307 | 49522406 | . | + | 1 | ID=CDS:PavDAM6.4;Parent=mRNA:PavDAM6;Ontology_term=SO:0000004;est_cons=100.0;est_incons=0.0 |
| PAV01_REGINA | EuGene_Manual | CDS | 49523208 | 49523249 | . | + | 0 | ID=CDS:PavDAM6.5;Parent=mRNA:PavDAM6;Ontology_term=SO:0000004;est_cons=100.0;est_incons=0.0 |
| PAV01_REGINA | EuGene_Manual | CDS | 49523384 | 49523425 | . | + | 0 | ID=CDS:PavDAM6.6;Parent=mRNA:PavDAM6;Ontology_term=SO:0000004;est_cons=100.0;est_incons=0.0 |
| PAV01_REGINA | EuGene_Manual | CDS | 49523526 | 49523722 | . | + | 0 | ID=CDS:PavDAM6.7;Parent=mRNA:PavDAM6;Ontology_term=SO:0000004;est_cons=100.0;est_incons=0.0 |
| PAV01_REGINA | EuGene_Manual | CDS | 49524409 | 49524418 | . | + | 1 | ID=CDS:PavDAM6.8;Parent=mRNA:PavDAM6;Ontology_term=SO:0000197;est_cons=100.0;est_incons=0.0 |
| PAV01_REGINA | EuGene_Manual | three_prime_UTR | 49524419 | 49524699 | . | + | . | ID=three_prime_UTR:PavDAM6.18;Parent=mRNA:PavDAM6;Ontology_term=SO:0000205;est_cons=100.0;est_incons=0.0 |

**Supplementary Table 4** Percentage of identity between amino acid sequences of six *PavDAM* genes of 14 sweet cherry cultivars.

|  | **Brooks** | **Cristobalina** | **Ferrovia** | **Hedelfingen** | **Lambert** | **Napoleon** | **Rainier** | **Regina** | **Sam** | **Satonishiki** | **Sue** | **Summit** | **Vic** |
| --- | --- | --- | --- | --- | --- | --- | --- | --- | --- | --- | --- | --- | --- |
| **Ambrunés** | 99.7 | 98.9 | 99.7 | 99.7 | 99.8 | 99.9 | 99.7 | 99.9 | 99.9 | 99.7 | 99.9 | 100 | 99.7 |
| **Brooks** |  | 98.9 | 99.7 | 99.9 | 99.8 | 99.8 | 99.9 | 99.8 | 99.8 | 99.9 | 99.8 | 99.7 | 100 |
| **Cristobalina** |  |  | 98.9 | 98.9 | 98.8 | 99.0 | 98.8 | 99.0 | 99.0 | 98.9 | 99.0 | 98.9 | 98.9 |
| **Ferrovia** |  |  |  | 99.7 | 99.7 | 99.8 | 99.7 | 99.8 | 99.8 | 99.7 | 99.8 | 99.7 | 99.7 |
| **Hedelfingen** |  |  |  |  | 99.8 | 99.8 | 99.8 | 99.8 | 99.8 | 99.9 | 99.8 | 99.7 | 99.9 |
| **Lambert** |  |  |  |  |  | 99.7 | 99.7 | 99.9 | 99.9 | 99.8 | 99.9 | 99.8 | 99.8 |
| **Napoleon** |  |  |  |  |  |  | 99.7 | 99.9 | 99.9 | 99.8 | 99.9 | 99.9 | 99.8 |
| **Rainier** |  |  |  |  |  |  |  | 99.7 | 99.7 | 99.8 | 99.7 | 99.7 | 99.9 |
| **Regina** |  |  |  |  |  |  |  |  | 100 | 99.8 | 100 | 99.9 | 99.8 |
| **Sam** |  |  |  |  |  |  |  |  |  | 99.8 | 100 | 99.9 | 99.8 |
| **Satonishiki** |  |  |  |  |  |  |  |  |  |  | 99.8 | 99.7 | 99.9 |
| **Sue** |  |  |  |  |  |  |  |  |  |  |  | 99.9 | 99.8 |
| **Summit** |  |  |  |  |  |  |  |  |  |  |  |  | 99.7 |

**Supplementary Table 5** Number of amino acid substitution in *PavDAMs* in 14 sweet cherry cultivars. Specific details of the substitutions in each gene and cultivar are shown in Sup Fig 1. Unique amino acid substitutions are highlighted in bold.

| **Gene** | ***PavDAM1*** | | | | ***PavDAM2*** | | | | ***PavDAM3*** | | | | ***PavDAM4*** | | | | ***PavDAM5*** | | | | ***PavDAM6*** | | | | **Total** |
| --- | --- | --- | --- | --- | --- | --- | --- | --- | --- | --- | --- | --- | --- | --- | --- | --- | --- | --- | --- | --- | --- | --- | --- | --- | --- |
| **Domain** | M | I | K | C | M | I | K | C | M | I | K | C | M | I | K | C | M | I | K | C | M | I | K | C | **(Unique)** |
| **‘Ambrunés’** |  |  |  |  |  |  |  |  |  |  |  |  |  |  |  |  |  |  |  | 1 |  |  |  |  | 1 |
| **‘Brooks’** |  |  |  | 1 |  |  |  |  |  |  |  |  | 1 |  |  |  |  |  |  |  |  | 1 |  |  | 3 |
| **‘Cristobalina’** |  | **3** |  | 1 | **1** |  | **1** |  |  | **1** | **1** |  |  | **2** |  | **2** | **1** | **1** |  | **1** |  |  |  |  | 15 (14) |
| **‘Ferrovia’** |  |  | **1** | 1 |  |  |  |  |  |  |  |  |  |  |  |  |  |  |  |  |  |  |  | **1** | 3 (2) |
| **‘Hedelfinger’** |  |  |  | 1 |  |  |  |  |  |  |  |  | 1 | **1** |  |  |  |  |  |  |  |  |  |  | 3 |
| **‘Lambert’** |  | **1** |  |  |  |  |  |  |  |  |  |  | 1 |  |  |  |  |  |  |  |  |  |  |  | 2 (1) |
| **‘Napoleon’** |  |  |  | 1 |  |  |  |  |  |  |  |  |  |  |  |  |  |  |  |  |  |  |  |  | 1 |
| **‘Rainier’** |  |  |  | 1 |  |  |  |  |  |  |  |  | 1 |  |  |  |  |  |  |  |  | 1 |  | **1** | 4 (1) |
| **‘Regina’** |  |  |  |  |  |  |  |  |  |  |  |  |  |  |  |  |  |  |  |  |  |  |  |  | 0 |
| **‘Sam’** |  |  |  |  |  |  |  |  |  |  |  |  |  |  |  |  |  |  |  |  |  |  |  |  | 0 |
| **‘Satonishiki’** |  |  |  | 1 |  |  |  |  |  |  |  |  | 1 |  |  |  |  |  |  | **1** |  |  |  |  | 3 (1) |
| **‘Sue’** |  |  |  |  |  |  |  |  |  |  |  |  |  |  |  |  |  |  |  |  |  |  |  |  | 0 |
| **‘Summit’** |  |  |  |  |  |  |  |  |  |  |  |  |  |  |  |  |  |  |  | 1 |  |  |  |  | 1 |
| **‘Vic’** |  |  |  | 1 |  |  |  |  |  |  |  |  | 1 |  |  |  |  |  |  |  |  | 1 |  |  | 3 |

**Supplementary Table 6** NewPLACE database motifs found in ‘Regina’ and absent in ‘Cristobalina’ upstream of *PavDAM1* (PavD1UM). In grey, motifs putatively involved in *PavDAMs* expression (position shown in Supplementary Figure 4.

| **ID** | **Motif** | **Accession** | **Definition** |
| --- | --- | --- | --- |
| 10PEHVPSBD | TATTCT | S000392 | Promoter element found in the barley chloroplast psbD gene promoter. Involved in the expression of the plastid gene psbD. |
| 300ELEMENT | TGHAAARK | S000122 | Present upstream of the promoter from the B-hordein gene of barley and the alpha-gliadin, gamma-gliadin, and low molecular weight glutenin genes of wheat. |
| ACGTATERD1 | ACGT | S000415 | ACGT sequence required for etiolation-induced expression of erd1 (early responsive to dehydration) in Arabidopsis. |
| AGCBOXNPGLB | AGCCGCC | S000232 | AGC box repeated twice in a 61 bp enhancer element in tobacco class I beta-1,3-glucanase (GLB) gene. |
| AMYBOX2 | TATCCAT | S000021 | Amylase box. |
| ANAERO2CONSENSUS | AGCAGC | S000478 | Motifs found in silico in promoters of 13 anaerobic genes involved in the fermentative pathway (anaerobic set 1). |
| ARFAT | TGTCTC | S000270 | ARF (auxin response factor) binding site found in the promoters of primary/early auxin response genes of Arabidopsis thaliana. |
| ARR1AT | NGATT | S000454 | ARR1-binding element found in Arabidopsis. |
| BIHD1OS | TGTCA | S000498 | Binding site of OsBIHD1, a rice BELL homeodomain transcription factor. |
| BOXIINTPATPB | ATAGAA | S000296 | Box II found in the tobacco plastid atpB gene promoter. |
| CAATBOX1 | CAAT | S000028 | CAAT promoter consensus sequence found in legA gene of pea. |
| CACTFTPPCA1 | YACT | S000449 | Tetranucleotide (CACT) is a key component of Mem1 found in the cis-regulatory element in the distal region of the phosphoenolpyruvate carboxylase (ppcA1) of the C4 dicot F. trinervia. |
| CARGCW8GAT | CWWWWWWWWG | S000431 | A variant of CArG motif, with a longer A/T-rich core. Binding site for AGL15 (AGAMOUS-like 15). |
| CATATGGMSAUR | CATATG | S000370 | Involved in auxin responsiveness. |
| CCA1ATLHCB1 | AAMAATCT | S000149 | CCA1 binding site. |
| CCAATBOX1 | CCAAT | S000030 | Common sequence found in the 5'-non-coding regions of eukaryotic genes. |
| CIACADIANLELHC | CAANNNNATC | S000252 | Region necessary for circadian expression of tomato Lhc gene. |
| CURECORECR | GTAC | S000493 | GTAC is the core of a CuRE (copper-response element) found in Cyc6 and Cpx1 genes in Chlamydomonas. Also involved in oxygen-response of these genes. |
| DOFCOREZM | AAAG | S000265 | Core site required for binding of Dof proteins in maize. |
| EBOXBNNAPA | CANNTG | S000144 | E-box of napA storage-protein gene of Brassica napus. |
| EECCRCAH1 | GANTTNC | S000494 | Consensus motif of the two enhancer elements, EE-1 and EE-2, both found in the promoter region of the Chlamydomonas Cah1. |
| GARE2OSREP1 | TAACGTA | S000420 | Gibberellin-responsive element (GARE) found in the promoter region of a cystein proteinase (REP-1) gene in rice. |
| GATABOX | GATA | S000039 | GATA box. |
| GCCCORE | GCCGCC | S000430 | Core of GCC-box found in many pathogen-responsive genes such as PDF1.2, Thi2.1, and PR4 that has been shown to function as ethylene-responsive element. |
| GT1CONSENSUS | GRWAAW | S000198 | Consensus GT-1 binding site in many light-regulated genes. |
| GT1GMSCAM4 | GAAAAA | S000453 | GT-1 motif found in the promoter of soybean CaM isoform, SCaM-4. Plays a role in pathogen- and salt-induced SCaM-4 gene expression. |
| GTGANTG10 | GTGA | S000378 | GTGA motif found in the promoter of the tobacco late pollen gene g10 which shows homology to pectate lyase. |
| INRNTPSADB | YTCANTYY | S000395 | Inr (initiator) elements found in the tobacco psaDb gene promoter without TATA boxes. |
| MARTBOX | TTWTWTTWTT | S000067 | T-Box; Motif found in SAR (scaffold attachment region; or matrix attachment region, MAR). |
| MYBPZM | CCWACC | S000179 | Core of consensus maize P (myb homolog) binding site. |
| MYBST1 | GGATA | S000180 | Core motif of MybSt1 (a potato MYB homolog) binding site. |
| MYCATERD1 | CATGTG | S000413 | MYC recognition sequence necessary for expression of erd1 (early responsive to dehydration) in dehydrated Arabidopsis. |
| MYCATRD22 | CACATG | S000174 | Binding site for MYC in Arabidopsis dehydration-resposive gene, rd22. |
| MYCCONSENSUSAT | CANNTG | S000407 | MYC recognition sequence in CBF3 promoter. Binding site of ICE1 that regulates the transcription of CBF/DREB1 genes in the cold in Arabidopsis. |
| NODCON1GM | AAAGAT | S000461 | One of two putative nodulin consensus sequences. |
| NODCON2GM | CTCTT | S000462 | One of two putative nodulin consensus sequences. |
| OSE1ROOTNODULE | AAAGAT | S000467 | One of the consensus sequence motifs of organ-specific elements (OSE) characteristic of the promoters activated in infected cells of root nodulesve nodulin consensus sequences. |
| OSE2ROOTNODULE | CTCTT | S000468 | One of the consensus sequence motifs of organ-specific elements (OSE) characteristic of the promoters activated in infected cells of root nodules. |
| POLASIG1 | AATAAA | S000080 | PolyA signal. |
| POLASIG2 | AATTAAA | S000081 | PolyA signal. Poly A signal found in rice alpha-amylase. |
| POLASIG3 | AATAAT | S000088 | Plant polyA signal. Consensus sequence for plant polyadenylation signal. |
| POLLEN1LELAT52 | AGAAA | S000245 | One of two co-dependent regulatory elements responsible for pollen specific activation of tomato lat52 gene. |
| RAV1AAT | CAACA | S000314 | Binding consensus sequence of Arabidopsis transcription factor, RAV1. |
| REALPHALGLHCB21 | AACCAA | S000362 | REalpha found in Lemna gibba Lhcb21 gene promoter. Binding site of proteins of whole-cell extracts. |
| ROOTMOTIFTAPOX1 | ATATT | S000098 | Motif found both in promoters of rolD. |
| RYREPEATBNNAPA | CATGCA | S000264 | RY repeat found in RY/G box of napA gene in Brassica napus. |
| S1FBOXSORPS1L21 | ATGGTA | S000223 | S1F box conserved both in spinach RPS1 and RPL21 genes encoding the plastid ribosomal protein S1 and L21, respectively. |
| SEF3MOTIFGM | AACCCA | S000115 | SEF3 binding site. |
| SEF4MOTIFGM7S | RTTTTTR | S000103 | SEF4 binding site. |
| SITEIIATCYTC | TGGGCY | S000474 | Site II element found in the promoter regions of cytochrome genes (Cytc-1, Cytc-2) in Arabidopsis. |
| SURECOREATSULTR11 | GAGAC | S000499 | Core of sulfur-responsive element (SURE) found in the promoter of SULTR1. |
| TATABOX5 | TTATTT | S000203 | TATA box. |
| TATCCAOSAMY | TATCCA | S000403 | TATCCA element found in alpha-amylase promoters OsMYBS2 and OsMYBS3 which mediate sugar and hormone regulation of alpha-amylase gene expression. |
| TATCCAYMOTIFOSRAMY3D | TATCCAY | S000256 | TATCCAY motif found in rice responsible for sugar repression. |
| WBOXATNPR1 | TTGAC | S000390 | W-box found in promoter of Arabidopsis thaliana NPR1 gene. They were recognized specifically by salicylic acid (SA)-induced WRKY DNA binding proteins. |
| WBOXHVISO1 | TGACT | S000442 | SUSIBA2 bind to W-box element in barley iso1 (encoding isoamylase1) promoter. |
| WBOXNTCHN48 | CTGACY | S000508 | W box identified in the tobacco class I basic chitinase gene CHN48. |
| WBOXNTERF3 | TGACY | S000457 | May be involved in activation of ERF3 gene by wounding. |
| WRKY71OS | TGAC | S000447 | Binding site of rice WRKY71, a transcriptional repressor of the gibberellin signaling pathway. |
| WUSATAg | TTAATGG | S000433 | Target sequence of WUS in the intron of AGAMOUS gene in Arabidopsis. |

**Supplementary Table 7** PavD1UM and PavD4/5M PCR marker genotypes in 61 individuals of B×C2 F_2_ population. Mean bloom time phenotype (2015-2018) in calendar days (CD), and bloom time QTL (*qP-BT1.1^m^*) haplotypes (Calle et al., 2020) are also shown. PavD1UM: *pp*=900 bp, *pn=*900/1600 bp, *nn*=1600 bp. PavD4/5M: *pp*=850 bp, *pn=850/950* bp, *nn*=950 bp.

| **Individual** | **CD** | ***qP-BT1.1^m^*** | **PavD1UM** | **PavD4/5M** |
| --- | --- | --- | --- | --- |
| B×C2-39 | 80 | *cc* | *pp* | *pp* |
| B×C2-20 | 80 | *cc* | *pp* | *pp* |
| B×C2-06 | 80 | *cc* | *pp* | *pp* |
| B×C2-49 | 81 | *cc* | *pp* | *pp* |
| B×C2-63 | 81 | *cc* | *pp* | *pp* |
| B×C2-44 | 81 | *cc* | *pp* | *pp* |
| B×C2-80 | 81 | *cc* | *pp* | *pp* |
| B×C2-17 | 82 | *cc* | *pp* | *pp* |
| B×C2-50 | 82 | *cc* | *pp* | *pp* |
| B×C2-61 | 82 | *cc* | *pp* | *pp* |
| B×C2-18 | 84 | *cc* | *pp* | *pp* |
| B×C2-30 | 84 | *cc* | *pp* | *pp* |
| B×C2-62 | 84 | *cc* | *pp* | *pp* |
| B×C2-01 | 84 | *cc* | *pp* | *pp* |
| B×C2-67 | 84 | *cc* | *pp* | *pp* |
| B×C2-04 | 85 | *cc* | *pp* | *pp* |
| B×C2-35 | 85 | *cc* | *pp* | *pp* |
| B×C2-15 | 85 | *cc* | *pp* | *pp* |
| B×C2-43 | 85 | *cc* | *pp* | *pp* |
| B×C2-53 | 86 | *cc* | *pp* | *pp* |
| B×C2-12 | - | *cc* | *pp* | *pp* |
| B×C2-66 | - | *Recombinant* | *pp* | *pp* |
| B×C2-73 | - | *Recombinant* | *pp* | *pp* |
| B×C2-76 | - | *Recombinant* | *pp* | *pp* |
| **Mean** | **83** |  |  |  |
| B×C2-19 | 87 | *ac* | *pn* | *pn* |
| B×C2-09 | 88 | *ac* | *pn* | *pn* |
| B×C2-31 | 88 | *ac* | *pn* | *pn* |
| B×C2-21 | 89 | *ac* | *pn* | *pn* |
| B×C2-22 | 89 | *ac* | *pn* | *pn* |
| B×C2-37 | 89 | *ac* | *pn* | *pn* |
| B×C2-23 | 89 | *ac* | *pn* | *pn* |
| B×C2-42 | 89 | *ac* | *pn* | *pn* |
| B×C2-48 | 89 | *Recombinant* | *pn* | *pn* |
| B×C2-16 | 90 | *ac* | *pn* | *pn* |
| B×C2-69 | 90 | *ac* | *pn* | *pn* |
| B×C2-11 | 90 | *ac* | *pn* | *pn* |
| B×C2-75 | 90 | *ac* | *pn* | *pn* |
| B×C2-28 | 90 | *ac* | *pn* | *pn* |
| B×C2-51 | 90 | *ac* | *pn* | *pn* |
| B×C2-32 | 90 | *ac* | *pn* | *pn* |
| B×C2-25 | 90 | *ac* | *pn* | *pn* |
| B×C2-08 | 91 | *ac* | *pn* | *pn* |
| B×C2-70 | 91 | *ac* | *pn* | *pn* |
| B×C2-05 | 92 | *ac* | *pn* | *pn* |
| B×C2-54 | 93 | *ac* | *pn* | *pn* |
| B×C2-72 | 93 | *ac* | *pn* | *pn* |
| B×C2-55 | 93 | *ac* | *pn* | *pn* |
| B×C2-29 | - | *Recombinant* | *pn* | *pn* |
| B×C2-34 | - | *Recombinant* | *pn* | *pn* |
| B×C2-41 | - | *Recombinant* | *pn* | *pn* |
| B×C2-47 | - | *Recombinant* | *pn* | *pn* |
| B×C2-57 | - | *Recombinant* | *pn* | *pn* |
| B×C2-59 | - | *Recombinant* | *pn* | *pn* |
| B×C2-60 | - | *Recombinant* | *pn* | *pn* |
| B×C2-65 | - | *Recombinant* | *pn* | *pn* |
| B×C2-74 | - | *Recombinant* | *pn* | *pn* |
| B×C2-77 | - | *Recombinant* | *pn* | *pn* |
| **Mean** | **90** |  |  |  |
| B×C2-26 | - | *aa* | *nn* | *nn* |
| B×C2-33 | - | *aa* | *nn* | *nn* |
| B×C2-36 | - | *aa* | *nn* | *nn* |
| B×C2-64 | - | *aa* | *nn* | *nn* |
| **Mean** | - |  |  |  |

**Supplementary Figure 1** Alignment of deduced amino acid sequences of *PavDAM* genes of 13 sweet cherry cultivars. Amino acid substitutions are highlight in yellow and marked with an asterisk. MADs-box domains are highlighted in colors (M: green; I: blue; K: pink and C; grey).

1 10 20 30 40 50 60

| | | | | | |

Ambrunes_DAM1 MKMMREKIKIKKIDNLPARQVTFSKRRRGIFKKAAELSVLCESEVAVIIFSATGKLFDYS

Brooks_DAM1 MKMMREKIKIKKIDNLPARQVTFSKRRRGIFKKAAELSVLCESEVAVIIFSATGKLFDYS

Cristobalina_DAM1 MKMMREKIKIKKIDNLPARQVTFSKRRRGIFKKAAELSVLCESEVAVIIFSATGKLFDYS

Ferrovia_DAM1 MKMMREKIKIKKIDNLPARQVTFSKRRRGIFKKAAELSVLCESEVAVIIFSATGKLFDYS

Helderfinger_DAM1 MKMMREKIKIKKIDNLPARQVTFSKRRRGIFKKAAELSVLCESEVAVIIFSATGKLFDYS

Lambert_DAM1 MKMMREKIKIKKIDNLPARQVTFSKRRRGIFKKAAELSVLCESEVAVIIFSATGKLFDYS

Napoleon_DAM1 MKMMREKIKIKKIDNLPARQVTFSKRRRGIFKKAAELSVLCESEVAVIIFSATGKLFDYS

Rainier_DAM1 MKMMREKIKIKKIDNLPARQVTFSKRRRGIFKKAAELSVLCESEVAVIIFSATGKLFDYS

Regina_DAM1 MKMMREKIKIKKIDNLPARQVTFSKRRRGIFKKAAELSVLCESEVAVIIFSATGKLFDYS

Sam_DAM1 MKMMREKIKIKKIDNLPARQVTFSKRRRGIFKKAAELSVLCESEVAVIIFSATGKLFDYS

Satonishiki_DAM1 MKMMREKIKIKKIDNLPARQVTFSKRRRGIFKKAAELSVLCESEVAVIIFSATGKLFDYS

Sue_DAM1 MKMMREKIKIKKIDNLPARQVTFSKRRRGIFKKAAELSVLCESEVAVIIFSATGKLFDYS

Summit_DAM1 MKMMREKIKIKKIDNLPARQVTFSKRRRGIFKKAAELSVLCESEVAVIIFSATGKLFDYS

Vic_DAM1 MKMMREKIKIKKIDNLPARQVTFSKRRRGIFKKAAELSVLCESEVAVIIFSATGKLFDYS

------------------------------------------------------------

Ambrunes_DAM1 SSSTKDVIERYKAHINGAEKSDEPSVELQPENENHIRLSKELGEKSRQLRQMKGEDLEEL

Brooks_DAM1 SSSTKDVIERYKAHINGAEKSDEPSVELQPENENHIRLSKELGEKSRQLRQMKGEDLEEL

Cristobalina_DAM1 SSSTKDVIERYKAHINGVEQSDEPYVELQPENENHIRLSKELGEKSRQLRQMKGEDLEEL

Ferrovia_DAM1 SSSTKDVIERYKAHINGAEKSDEPSVELQPENENHIRLSEELGEKSRQLRQMKGEDLEEL

Helderfinger_DAM1 SSSTKDVIERYKAHINGAEKSDEPSVELQPENENHIRLSKELGEKSRQLRQMKGEDLEEL

Lambert_DAM1 SSSTKDVIERYKAHINGAEKTDEPSVELQPENENHIRLSKELGEKSRQLRQMKGEDLEEL

Napoleon_DAM1 SSSTKDVIERYKAHINGAEKSDEPSVELQPENENHIRLSKELGEKSRQLRQMKGEDLEEL

Rainier_DAM1 SSSTKDVIERYKAHINGAEKSDEPSVELQPENENHIRLSKELGEKSRQLRQMKGEDLEEL

Regina_DAM1 SSSTKDVIERYKAHINGAEKSDEPSVELQPENENHIRLSKELGEKSRQLRQMKGEDLEEL

Sam_DAM1 SSSTKDVIERYKAHINGAEKSDEPSVELQPENENHIRLSKELGEKSRQLRQMKGEDLEEL

Satonishiki_DAM1 SSSTKDVIERYKAHINGAEKSDEPSVELQPENENHIRLSKELGEKSRQLRQMKGEDLEEL

Sue_DAM1 SSSTKDVIERYKAHINGAEKSDEPSVELQPENENHIRLSKELGEKSRQLRQMKGEDLEEL

Summit_DAM1 SSSTKDVIERYKAHINGAEKSDEPSVELQPENENHIRLSKELGEKSRQLRQMKGEDLEEL

Vic_DAM1 SSSTKDVIERYKAHINGAEKSDEPSVELQPENENHIRLSKELGEKSRQLRQMKGEDLEEL

-----------------*-**---*--------------*--------------------

Ambrunes_DAM1 NFDELQKLEQLVDASLGRVIETKDELIMSEIMALERKRSELVEANKQLRQRMLSRRNIGP

Brooks_DAM1 NFDELQKLEQLVDASLGRVIETKDELIMSEIMALERKRSELVEANKQLRQRMLSRRNIGP

Cristobalina_DAM1 NFDELQKLEQLVDASLGRVIETKDELIMSEIMALERKRSELVEANKQLRQRMLSRRNIGP

Ferrovia_DAM1 NFDELQKLEQLVDASLGRVIETKDELIMSEIMALERKRSELVEANKQLRQRMLSRRNIGP

Helderfinger_DAM1 NFDELQKLEQLVDASLGRVIETKDELIMSEIMALERKRSELVEANKQLRQRMLSRRNIGP

Lambert_DAM1 NFDELQKLEQLVDASLGRVIETKDELIMSEIMALERKRSELVEANKQLRQRMLSRRNIGP

Napoleon_DAM1 NFDELQKLEQLVDASLGRVIETKDELIMSEIMALERKRSELVEANKQLRQRMLSRRNIGP

Rainier_DAM1 NFDELQKLEQLVDASLGRVIETKDELIMSEIMALERKRSELVEANKQLRQRMLSRRNIGP

Regina_DAM1 NFDELQKLEQLVDASLGRVIETKDELIMSEIMALERKRSELVEANKQLRQRMLSRRNIGP

Sam_DAM1 NFDELQKLEQLVDASLGRVIETKDELIMSEIMALERKRSELVEANKQLRQRMLSRRNIGP

Satonishiki_DAM1 NFDELQKLEQLVDASLGRVIETKDELIMSEIMALERKRSELVEANKQLRQRMLSRRNIGP

Sue_DAM1 NFDELQKLEQLVDASLGRVIETKDELIMSEIMALERKRSELVEANKQLRQRMLSRRNIGP

Summit_DAM1 NFDELQKLEQLVDASLGRVIETKDELIMSEIMALERKRSELVEANKQLRQRMLSRRNIGP

Vic_DAM1 NFDELQKLEQLVDASLGRVIETKDELIMSEIMALERKRSELVEANKQLRQRMLSRRNIGP

------------------------------------------------------------

Ambrunes_DAM1 ALMEPERLNNNIGGGGEEEGMSSESATSTTCNSAPCPSLEDDSDDVTLSLKLGLL

Brooks_DAM1 ALMEPERLNNNIGGGGEEEGMSSESATSTTCNSAPCLSLEDDSDDVTLSLKLGLL

Cristobalina_DAM1 ALMEPERLNNNIGGGGEEEGMSSESATSTTCNSAPCLSLEDDSDDVTLSLKLGLL

Ferrovia_DAM1 ALMEPERLNNNIGGGGEEEGMSSESATSTTCNSAPCLSLEDDSDDVTLSLKLGLL

Helderfinger_DAM1 ALMEPERLNNNIGGGGEEEGMSSESATSTTCNSAPCLSLEDDSDDVTLSLKLGLL

Lambert_DAM1 ALMEPERLNNNIGGGGEEEGMSSESATSTTCNSAPCPSLEDDSDDVTLSLKLGLL

Napoleon_DAM1 ALMEPERLNNNIGGGGEEEGMSSESATSTTCNSAPCLSLEDDSDDVTLSLKLGLL

Rainier_DAM1 ALMEPERLNNNIGGGGEEEGMSSESATSTTCNSAPCLSLEDDSDDVTLSLKLGLL

Regina_DAM1 ALMEPERLNNNIGGGGEEEGMSSESATSTTCNSAPCPSLEDDSDDVTLSLKLGLL

Sam_DAM1 ALMEPERLNNNIGGGGEEEGMSSESATSTTCNSAPCPSLEDDSDDVTLSLKLGLL

Satonishiki_DAM1 ALMEPERLNNNIGGGGEEEGMSSESATSTTCNSAPCLSLEDDSDDVTLSLKLGLL

Sue_DAM1 ALMEPERLNNNIGGGGEEEGMSSESATSTTCNSAPCPSLEDDSDDVTLSLKLGLL

Summit_DAM1 ALMEPERLNNNIGGGGEEEGMSSESATSTTCNSAPCPSLEDDSDDVTLSLKLGLL

Vic_DAM1 ALMEPERLNNNIGGGGEEEGMSSESATSTTCNSAPCLSLEDDSDDVTLSLKLGLL

------------------------------------*------------------

**Supplementary Figure 1** Continued.

1 10 20 30 40 50 60

| | | | | | |

Ambrunes_DAM2 MVKMMRKKIKIKKIDYLPARQVTFSKRRRGIFKKAKELSVLCESEVAVIIFSATGKLFDY

Brooks_DAM2 MVKMMRKKIKIKKIDYLPARQVTFSKRRRGIFKKAKELSVLCESEVAVIIFSATGKLFDY

Cristobalina_DAM2 MVKMMRKKIKIKKIDHLPARQVTFSKRRRGIFKKAKELSVLCESEVAVIIFSATGKLFDY

Ferrovia_DAM2 MVKMMRKKIKIKKIDYLPARQVTFSKRRRGIFKKAKELSVLCESEVAVIIFSATGKLFDY

Helderfinger_DAM2 MVKMMRKKIKIKKIDYLPARQVTFSKRRRGIFKKAKELSVLCESEVAVIIFSATGKLFDY

Lambert_DAM2 MVKMMRKKIKIKKIDYLPARQVTFSKRRRGIFKKAKELSVLCESEVAVIIFSATGKLFDY

Napoleon_DAM2 MVKMMRKKIKIKKIDYLPARQVTFSKRRRGIFKKAKELSVLCESEVAVIIFSATGKLFDY

Rainier_DAM2 MVKMMRKKIKIKKIDYLPARQVTFSKRRRGIFKKAKELSVLCESEVAVIIFSATGKLFDY

Regina_DAM2 MVKMMRKKIKIKKIDYLPARQVTFSKRRRGIFKKAKELSVLCESEVAVIIFSATGKLFDY

Sam_DAM2 MVKMMRKKIKIKKIDYLPARQVTFSKRRRGIFKKAKELSVLCESEVAVIIFSATGKLFDY

Satonishiki_DAM2 MVKMMRKKIKIKKIDYLPARQVTFSKRRRGIFKKAKELSVLCESEVAVIIFSATGKLFDY

Sue_DAM2 MVKMMRKKIKIKKIDYLPARQVTFSKRRRGIFKKAKELSVLCESEVAVIIFSATGKLFDY

Summit_DAM2 MVKMMRKKIKIKKIDYLPARQVTFSKRRRGIFKKAKELSVLCESEVAVIIFSATGKLFDY

Vic_DAM2 MVKMMRKKIKIKKIDYLPARQVTFSKRRRGIFKKAKELSVLCESEVAVIIFSATGKLFDY

---------------*--------------------------------------------

Ambrunes_DAM2 SSSSTKDVVERYKAHTNSVEKSDELSVELQLEIENHIRLTKELEAKSRQLRMKGEDLEEL

Brooks_DAM2 SSSSTKDVVERYKAHTNSVEKSDELSVELQLEIENHIRLTKELEAKSRQLRMKGEDLEEL

Cristobalina_DAM2 SSSSTKDVVERYKAHTNSVEKSDELSVELQLEIENHIRLTKELEAKSRQLRMKGEDLEEL

Ferrovia_DAM2 SSSSTKDVVERYKAHTNSVEKSDELSVELQLEIENHIRLTKELEAKSRQLRMKGEDLEEL

Helderfinger_DAM2 SSSSTKDVVERYKAHTNSVEKSDELSVELQLEIENHIRLTKELEAKSRQLRMKGEDLEEL

Lambert_DAM2 SSSSTKDVVERYKAHTNSVEKSDELSVELQLEIENHIRLTKELEAKSRQLRMKGEDLEEL

Napoleon_DAM2 SSSSTKDVVERYKAHTNSVEKSDELSVELQLEIENHIRLTKELEAKSRQLRMKGEDLEEL

Rainier_DAM2 SSSSTKDVVERYKAHTNSVEKSDELSVELQLEIENHIRLTKELEAKSRQLRMKGEDLEEL

Regina_DAM2 SSSSTKDVVERYKAHTNSVEKSDELSVELQLEIENHIRLTKELEAKSRQLRMKGEDLEEL

Sam_DAM2 SSSSTKDVVERYKAHTNSVEKSDELSVELQLEIENHIRLTKELEAKSRQLRMKGEDLEEL

Satonishiki_DAM2 SSSSTKDVVERYKAHTNSVEKSDELSVELQLEIENHIRLTKELEAKSRQLRMKGEDLEEL

Sue_DAM2 SSSSTKDVVERYKAHTNSVEKSDELSVELQLEIENHIRLTKELEAKSRQLRMKGEDLEEL

Summit_DAM2 SSSSTKDVVERYKAHTNSVEKSDELSVELQLEIENHIRLTKELEAKSRQLRMKGEDLEEL

Vic_DAM2 SSSSTKDVVERYKAHTNSVEKSDELSVELQLEIENHIRLTKELEAKSRQLRMKGEDLEEL

------------------------------------------------------------

Ambrunes_DAM2 NFDELHKLEQLVDASLGRAIETEEELNMSEIMALERKEAELVEANNQLRQRMLSRGNIGP

Brooks_DAM2 NFDELHKLEQLVDASLGRAIETEEELNMSEIMALERKEAELVEANNQLRQRMLSRGNIGP

Cristobalina_DAM2 NFDELQKLEQLVDASLGRAIETEEELNMSEIMALERKEAELVEANNQLRQRMLSRGNIGP

Ferrovia_DAM2 NFDELHKLEQLVDASLGRAIETEEELNMSEIMALERKEAELVEANNQLRQRMLSRGNIGP

Helderfinger_DAM2 NFDELHKLEQLVDASLGRAIETEEELNMSEIMALERKEAELVEANNQLRQRMLSRGNIGP

Lambert_DAM2 NFDELHKLEQLVDASLGRAIETEEELNMSEIMALERKEAELVEANNQLRQRMLSRGNIGP

Napoleon_DAM2 NFDELHKLEQLVDASLGRAIETEEELNMSEIMALERKEAELVEANNQLRQRMLSRGNIGP

Rainier_DAM2 NFDELHKLEQLVDASLGRAIETEEELNMSEIMALERKEAELVEANNQLRQRMLSRGNIGP

Regina_DAM2 NFDELHKLEQLVDASLGRAIETEEELNMSEIMALERKEAELVEANNQLRQRMLSRGNIGP

Sam_DAM2 NFDELHKLEQLVDASLGRAIETEEELNMSEIMALERKEAELVEANNQLRQRMLSRGNIGP

Satonishiki_DAM2 NFDELHKLEQLVDASLGRAIETEEELNMSEIMALERKEAELVEANNQLRQRMLSRGNIGP

Sue_DAM2 NFDELHKLEQLVDASLGRAIETEEELNMSEIMALERKEAELVEANNQLRQRMLSRGNIGP

Summit_DAM2 NFDELHKLEQLVDASLGRAIETEEELNMSEIMALERKEAELVEANNQLRQRMLSRGNIGP

Vic_DAM2 NFDELHKLEQLVDASLGRAIETEEELNMSEIMALERKEAELVEANNQLRQRMLSRGNIGP

-----*------------------------------------------------------

Ambrunes_DAM2 ALMEPERLINNIGGGGEEEGMSSESATNATISSCSSGLSLSLEDDCSDVTLALKLGLP

Brooks_DAM2 ALMEPERLINNIGGGGEEEGMSSESATNATISSCSSGLSLSLEDDCSDVTLALKLGLP

Cristobalina_DAM2 ALMEPERLINNIGGGGEEEGMSSESATNATISSCSSGLSLSLEDDCSDVTLALKLGLP

Ferrovia_DAM2 ALMEPERLINNIGGGGEEEGMSSESATNATISSCSSGLSLSLEDDCSDVTLALKLGLP

Helderfinger_DAM2 ALMEPERLINNIGGGGEEEGMSSESATNATISSCSSGLSLSLEDDCSDVTLALKLGLP

Lambert_DAM2 ALMEPERLINNIGGGGEEEGMSSESATNATISSCSSGLSLSLEDDCSDVTLALKLGLP

Napoleon_DAM2 ALMEPERLINNIGGGGEEEGMSSESATNATISSCSSGLSLSLEDDCSDVTLALKLGLP

Rainier_DAM2 ALMEPERLINNIGGGGEEEGMSSESATNATISSCSSGLSLSLEDDCSDVTLALKLGLP

Regina_DAM2 ALMEPERLINNIGGGGEEEGMSSESATNATISSCSSGLSLSLEDDCSDVTLALKLGLP

Sam_DAM2 ALMEPERLINNIGGGGEEEGMSSESATNATISSCSSGLSLSLEDDCSDVTLALKLGLP

Satonishiki_DAM2 ALMEPERLINNIGGGGEEEGMSSESATNATISSCSSGLSLSLEDDCSDVTLALKLGLP

Sue_DAM2 ALMEPERLINNIGGGGEEEGMSSESATNATISSCSSGLSLSLEDDCSDVTLALKLGLP

Summit_DAM2 ALMEPERLINNIGGGGEEEGMSSESATNATISSCSSGLSLSLEDDCSDVTLALKLGLP

Vic_DAM2 ALMEPERLINNIGGGGEEEGMSSESATNATISSCSSGLSLSLEDDCSDVTLALKLGLP

----------------------------------------------------------

**Supplementary Figure 1** Continued.

1 10 20 30 40 50 60

| | | | | | |

Ambrunes_DAM3 MVKMMRKKIKIKKIDCLPARQVTFSKRRRGIFKKAAELSVLCESKVAVVIFSATGKLFDY

Brooks_DAM3 MVKMMRKKIKIKKIDCLPARQVTFSKRRRGIFKKAAELSVLCESKVAVVIFSATGKLFDY

Cristobalina_DAM3 MVKMMRKKIKIKKIDCLPARQVTFSKRRRGIFKKAAELSVLCESKVAVVIFSATGKLFDY

Ferrovia_DAM3 MVKMMRKKIKIKKIDCLPARQVTFSKRRRGIFKKAAELSVLCESKVAVVIFSATGKLFDY

Helderfinger_DAM3 MVKMMRKKIKIKKIDCLPARQVTFSKRRRGIFKKAAELSVLCESKVAVVIFSATGKLFDY

Napoleon_DAM3 MVKMMRKKIKIKKIDCLPARQVTFSKRRRGIFKKAAELSVLCESKVAVVIFSATGKLFDY

Lambert_DAM3 MVKMMRKKIKIKKIDCLPARQVTFSKRRRGIFKKAAELSVLCESKVAVVIFSATGKLFDY

Rainier_DAM3 MVKMMRKKIKIKKIDCLPARQVTFSKRRRGIFKKAAELSVLCESKVAVVIFSATGKLFDY

Regina_DAM3 MVKMMRKKIKIKKIDCLPARQVTFSKRRRGIFKKAAELSVLCESKVAVVIFSATGKLFDY

Sam_DAM3 MVKMMRKKIKIKKIDCLPARQVTFSKRRRGIFKKAAELSVLCESKVAVVIFSATGKLFDY

Satonishiki_DAM3 MVKMMRKKIKIKKIDCLPARQVTFSKRRRGIFKKAAELSVLCESKVAVVIFSATGKLFDY

Sue_DAM3 MVKMMRKKIKIKKIDCLPARQVTFSKRRRGIFKKAAELSVLCESKVAVVIFSATGKLFDY

Summit_DAM3 MVKMMRKKIKIKKIDCLPARQVTFSKRRRGIFKKAAELSVLCESKVAVVIFSATGKLFDY

Vic_DAM3 MVKMMRKKIKIKKIDCLPARQVTFSKRRRGIFKKAAELSVLCESKVAVVIFSATGKLFDY

------------------------------------------------------------

Ambrunes_DAM3 SSSSTKDVIERYKAHTNGVEKSDEPSVELQLENENHIGLSKELEEKSHQLRQMKAEDLEE

Brooks_DAM3 SSSSTKDVIERYKAHTNGVEKSDEPSVELQLENENHIGLSKELEEKSHQLRQMKAEDLEE

Cristobalina_DAM3 SSSSTKDVIERYKAHTNGVKKSDEPSVELQLENENHIGLSKELEEKSHELRQMKAEDLEE

Ferrovia_DAM3 SSSSTKDVIERYKAHTNGVEKSDEPSVELQLENENHIGLSKELEEKSHQLRQMKAEDLEE

Helderfinger_DAM3 SSSSTKDVIERYKAHTNGVEKSDEPSVELQLENENHIGLSKELEEKSHQLRQMKAEDLEE

Napoleon_DAM3 SSSSTKDVIERYKAHTNGVEKSDEPSVELQLENENHIGLSKELEEKSHQLRQMKAEDLEE

Lambert_DAM3 SSSSTKDVIERYKAHTNGVEKSDEPSVELQLENENHIGLSKELEEKSHQLRQMKAEDLEE

Rainier_DAM3 SSSSTKDVIERYKAHTNGVEKSDEPSVELQLENENHIGLSKELEEKSHQLRQMKAEDLEE

Regina_DAM3 SSSSTKDVIERYKAHTNGVEKSDEPSVELQLENENHIGLSKELEEKSHQLRQMKAEDLEE

Sam_DAM3 SSSSTKDVIERYKAHTNGVEKSDEPSVELQLENENHIGLSKELEEKSHQLRQMKAEDLEE

Satonishiki_DAM3 SSSSTKDVIERYKAHTNGVEKSDEPSVELQLENENHIGLSKELEEKSHQLRQMKAEDLEE

Sue_DAM3 SSSSTKDVIERYKAHTNGVEKSDEPSVELQLENENHIGLSKELEEKSHQLRQMKAEDLEE

Summit_DAM3 SSSSTKDVIERYKAHTNGVEKSDEPSVELQLENENHIGLSKELEEKSHQLRQMKAEDLEE

Vic_DAM3 SSSSTKDVIERYKAHTNGVEKSDEPSVELQLENENHIGLSKELEEKSHQLRQMKAEDLEE

-------------------*----------------------------*-----------

Ambrunes_DAM3 LNFDELQKLEQLVDASLGRVIETKEELRMSEIMALERKGAELVEANNQLRQTMVMLSGGN

Brooks_DAM3 LNFDELQKLEQLVDASLGRVIETKEELRMSEIMALERKGAELVEANNQLRQTMVMLSGGN

Cristobalina_DAM3 LNFDELQKLEQLVDASLGRVIETKEELRMSEIMALERKGAELVEANNQLRQTMVMLSGGN

Ferrovia_DAM3 LNFDELQKLEQLVDASLGRVIETKEELRMSEIMALERKGAELVEANNQLRQTMVMLSGGN

Helderfinger_DAM3 LNFDELQKLEQLVDASLGRVIETKEELRMSEIMALERKGAELVEANNQLRQTMVMLSGGN

Napoleon_DAM3 LNFDELQKLEQLVDASLGRVIETKEELRMSEIMALERKGAELVEANNQLRQTMVMLSGGN

Lambert_DAM3 LNFDELQKLEQLVDASLGRVIETKEELRMSEIMALERKGAELVEANNQLRQTMVMLSGGN

Rainier_DAM3 LNFDELQKLEQLVDASLGRVIETKEELRMSEIMALERKGAELVEANNQLRQTMVMLSGGN

Regina_DAM3 LNFDELQKLEQLVDASLGRVIETKEELRMSEIMALERKGAELVEANNQLRQTMVMLSGGN

Sam_DAM3 LNFDELQKLEQLVDASLGRVIETKEELRMSEIMALERKGAELVEANNQLRQTMVMLSGGN

Satonishiki_DAM3 LNFDELQKLEQLVDASLGRVIETKEELRMSEIMALERKGAELVEANNQLRQTMVMLSGGN

Sue_DAM3 LNFDELQKLEQLVDASLGRVIETKEELRMSEIMALERKGAELVEANNQLRQTMVMLSGGN

Summit_DAM3 LNFDELQKLEQLVDASLGRVIETKEELRMSEIMALERKGAELVEANNQLRQTMVMLSGGN

Vic_DAM3 LNFDELQKLEQLVDASLGRVIETKEELRMSEIMALERKGAELVEANNQLRQTMVMLSGGN

------------------------------------------------------------

Ambrunes_DAM3 TGPELMEPERLNNNTGGGGEEEGMSTESAISTTCNSAHSLGDDSDNVTLSLKLGLP

Brooks_DAM3 TGPELMEPERLNNNTGGGGEEEGMSTESAISTTCNSAHSLGDDSDNVTLSLKLGLP

Cristobalina_DAM3 TGPELMEPERLNNNTGGGGEEEGMSTESAISTTCNSAHSLGDDSDNVTLSLKLGLP

Ferrovia_DAM3 TGPELMEPERLNNNTGGGGEEEGMSTESAISTTCNSAHSLGDDSDNVTLSLKLGLP

Helderfinger_DAM3 TGPELMEPERLNNNTGGGGEEEGMSTESAISTTCNSAHSLGDDSDNVTLSLKLGLP

Napoleon_DAM3 TGPELMEPERLNNNTGGGGEEEGMSTESAISTTCNSAHSLGDDSDNVTLSLKLGLP

Lambert_DAM3 TGPELMEPERLNNNTGGGGEEEGMSTESAISTTCNSAHSLGDDSDNVTLSLKLGLP

Rainier_DAM3 TGPELMEPERLNNNTGGGGEEEGMSTESAISTTCNSAHSLGDDSDNVTLSLKLGLP

Regina_DAM3 TGPELMEPERLNNNTGGGGEEEGMSTESAISTTCNSAHSLGDDSDNVTLSLKLGLP

Sam_DAM3 TGPELMEPERLNNNTGGGGEEEGMSTESAISTTCNSAHSLGDDSDNVTLSLKLGLP

Satonishiki_DAM3 TGPELMEPERLNNNTGGGGEEEGMSTESAISTTCNSAHSLGDDSDNVTLSLKLGLP

Sue_DAM3 TGPELMEPERLNNNTGGGGEEEGMSTESAISTTCNSAHSLGDDSDNVTLSLKLGLP

Summit_DAM3 TGPELMEPERLNNNTGGGGEEEGMSTESAISTTCNSAHSLGDDSDNVTLSLKLGLP

Vic_DAM3 TGPELMEPERLNNNTGGGGEEEGMSTESAISTTCNSAHSLGDDSDNVTLSLKLGLP

--------------------------------------------------------

**Supplementary Figure 1** Continued.

1 10 20 30 40 50 60

| | | | | | |

Ambrunes_DAM4 MVKMMREKIKIKKIDYLPARQVTFSKRRRGIFKKAAELSVLCESEVAVVIFSATGKLFDY

Brooks_DAM4 MVKMMREKIKIKKIDYLPARQVTFSKRRRGIFKKAAELSVLCESEVAVVIFSATGKLFYY

Cristobalina_DAM4 MVKMMREKIKIKKIDYLPARQVTFSKRRRGIFKKAAELSVLCESEVAVVIFSATGKLFDY

Ferrovia_DAM4 MVKMMREKIKIKKIDYLPARQVTFSKRRRGIFKKAAELSVLCESEVAVVIFSATGKLFDY

Helderfinger_DAM4 MVKMMREKIKIKKIDYLPARQVTFSKRRRGIFKKAAELSVLCESEVAVVIFSATGKLFYY

Lambert_DAM4 MVKMMREKIKIKKIDYLPARQVTFSKRRRGIFKKAAELSVLCESEVAVVIFSATGKLFYY

Napoleon_DAM4 MVKMMREKIKIKKIDYLPARQVTFSKRRRGIFKKAAELSVLCESEVAVVIFSATGKLFDY

Rainier_DAM4 MVKMMREKIKIKKIDYLPARQVTFSKRRRGIFKKAAELSVLCESEVAVVIFSATGKLFYY

Regina_DAM4 MVKMMREKIKIKKIDYLPARQVTFSKRRRGIFKKAAELSVLCESEVAVVIFSATGKLFDY

Sam_DAM4 MVKMMREKIKIKKIDYLPARQVTFSKRRRGIFKKAAELSVLCESEVAVVIFSATGKLFDY

Satonishiki_DAM4 MVKMMREKIKIKKIDYLPARQVTFSKRRRGIFKKAAELSVLCESEVAVVIFSATGKLFYY

Sue_DAM4 MVKMMREKIKIKKIDYLPARQVTFSKRRRGIFKKAAELSVLCESEVAVVIFSATGKLFDY

Summit_DAM4 MVKMMREKIKIKKIDYLPARQVTFSKRRRGIFKKAAELSVLCESEVAVVIFSATGKLFDY

Vic_DAM4 MVKMMREKIKIKKIDYLPARQVTFSKRRRGIFKKAAELSVLCESEVAVVIFSATGKLFYY

----------------------------------------------------------*-

Ambrunes_DAM4 SSSSVKDVIERYKARTNGVEKSDKSLELQLENENRIKLSKELEEKNRQLRKMKGEDLEEL

Brooks_DAM4 SSSSVKDVIERYKARTNGVEKSDKSLELQLENENRIKLSKELEEKNRQLRKMKGEDLEEL

Cristobalina_DAM4 SSSSVKDIIERYKARTNGVEKSDESLELQLENENRIKLSKELEEKNRQLRKMKGEDLEEL

Ferrovia_DAM4 SSSSVKDVIERYKARTNGVEKSDKSLELQLENENRIKLSKELEEKNRQLRKMKGEDLEEL

Helderfinger_DAM4 SSSSVKDVIESYKARTNGVEKSDKSLELQLENENRIKLSKELEEKNRQLRKMKGEDLEEL

Lambert_DAM4 SSSSVKDVIERYKARTNGVEKSDKSLELQLENENRIKLSKELEEKNRQLRKMKGEDLEEL

Napoleon_DAM4 SSSSVKDVIERYKARTNGVEKSDKSLELQLENENRIKLSKELEEKNRQLRKMKGEDLEEL

Rainier_DAM4 SSSSVKDVIERYKARTNGVEKSDKSLELQLENENRIKLSKELEEKNRQLRKMKGEDLEEL

Regina_DAM4 SSSSVKDVIERYKARTNGVEKSDKSLELQLENENRIKLSKELEEKNRQLRKMKGEDLEEL

Sam_DAM4 SSSSVKDVIERYKARTNGVEKSDKSLELQLENENRIKLSKELEEKNRQLRKMKGEDLEEL

Satonishiki_DAM4 SSSSVKDVIERYKARTNGVEKSDKSLELQLENENRIKLSKELEEKNRQLRKMKGEDLEEL

Sue_DAM4 SSSSVKDVIERYKARTNGVEKSDKSLELQLENENRIKLSKELEEKNRQLRKMKGEDLEEL

Summit_DAM4 SSSSVKDVIERYKARTNGVEKSDKSLELQLENENRIKLSKELEEKNRQLRKMKGEDLEEL

Vic_DAM4 SSSSVKDVIERYKARTNGVEKSDKSLELQLENENRIKLSKELEEKNRQLRKMKGEDLEEL

-------*--*------------*------------------------------------

Ambrunes_DAM4 DLDELLKLEQLVEATLVRVMETKEELIMSDIMVLEKKGTELVEANNQMVMLKERMVMLSK

Brooks_DAM4 DLDELLKLEQLVEATLVRVMETKEELIMSDIMVLEKKGTELVEANNQMVMLKERMVMLSK

Cristobalina_DAM4 DLDELLKLEQLVEATLVRVMETKEELIMSDIMVLEKKGTELVEANNQMVMLKERMVMLSK

Ferrovia_DAM4 DLDELLKLEQLVEATLVRVMETKEELIMSDIMVLEKKGTELVEANNQMVMLKERMVMLSK

Helderfinger_DAM4 DLDELLKLEQLVEATLVRVMETKEELIMSDIMVLEKKGTELVEANNQMVMLKERMVMLSK

Lambert_DAM4 DLDELLKLEQLVEATLVRVMETKEELIMSDIMVLEKKGTELVEANNQMVMLKERMVMLSK

Napoleon_DAM4 DLDELLKLEQLVEATLVRVMETKEELIMSDIMVLEKKGTELVEANNQMVMLKERMVMLSK

Rainier_DAM4 DLDELLKLEQLVEATLVRVMETKEELIMSDIMVLEKKGTELVEANNQMVMLKERMVMLSK

Regina_DAM4 DLDELLKLEQLVEATLVRVMETKEELIMSDIMVLEKKGTELVEANNQMVMLKERMVMLSK

Sam_DAM4 DLDELLKLEQLVEATLVRVMETKEELIMSDIMVLEKKGTELVEANNQMVMLKERMVMLSK

Satonishiki_DAM4 DLDELLKLEQLVEATLVRVMETKEELIMSDIMVLEKKGTELVEANNQMVMLKERMVMLSK

Sue_DAM4 DLDELLKLEQLVEATLVRVMETKEELIMSDIMVLEKKGTELVEANNQMVMLKERMVMLSK

Summit_DAM4 DLDELLKLEQLVEATLVRVMETKEELIMSDIMVLEKKGTELVEANNQMVMLKERMVMLSK

Vic_DAM4 DLDELLKLEQLVEATLVRVMETKEELIMSDIMVLEKKGTELVEANNQMVMLKERMVMLSK

------------------------------------------------------------

Ambrunes_DAM4 RNTEPAHMEPSESATSTSCNSALSLSGEDDCSDDVILSLKLGRP

Brooks_DAM4 RNTEPAHMEPSESATSTSCNSALSLSGEDDCSDDVILSLKLGRP

Cristobalina_DAM4 RNTGPAHMEPSESATSTSCNSALSLSLEDDCSDDVILSLKLGRP

Ferrovia_DAM4 RNTEPAHMEPSESATSTSCNSALSLSGEDDCSDDVILSLKLGRP

Helderfinger_DAM4 RNTEPAHMEPSESATSTSCNSALSLSGEDDCSDDVILSLKLGRP

Lambert_DAM4 RNTEPAHMEPSESATSTSCNSALSLSGEDDCSDDVILSLKLGRP

Napoleon_DAM4 RNTEPAHMEPSESATSTSCNSALSLSGEDDCSDDVILSLKLGRP

Rainier_DAM4 RNTEPAHMEPSESATSTSCNSALSLSGEDDCSDDVILSLKLGRP

Regina_DAM4 RNTEPAHMEPSESATSTSCNSALSLSGEDDCSDDVILSLKLGRP

Sam_DAM4 RNTEPAHMEPSESATSTSCNSALSLSGEDDCSDDVILSLKLGRP

Satonishiki_DAM4 RNTEPAHMEPSESATSTSCNSALSLSGEDDCSDDVILSLKLGRP

Sue_DAM4 RNTEPAHMEPSESATSTSCNSALSLSGEDDCSDDVILSLKLGRP

Summit_DAM4 RNTEPAHMEPSESATSTSCNSALSLSGEDDCSDDVILSLKLGRP

Vic_DAM4 RNTEPAHMEPSESATSTSCNSALSLSGEDDCSDDVILSLKLGRP

---*----------------------*-----------------

**Supplementary Figure 1** Continued.

1 10 20 30 40 50 60

| | | | | | |

Ambrunes_DAM5 MRNKIKIKKIDYLPARQVTFSKRRRGLFKKAAELSVLCESEVAVVIFSATGKLFDYSSSS

Brooks_DAM5 MRNKIKIKKIDYLPARQVTFSKRRRGLFKKAAELSVLCESEVAVVIFSATGKLFDYSSSS

Cristobalina_DAM5 MRNKIKIKKIDYLPARQVTFSKRRRGLFKKAAELSVLCESEVAVVIFSATGKLFEYSSSS

Ferrovia_DAM5 MRNKIKIKKIDYLPARQVTFSKRRRGLFKKAAELSVLCESEVAVVIFSATGKLFDYSSSS

Helderfinger_DAM5 MRNKIKIKKIDYLPARQVTFSKRRRGLFKKAAELSVLCESEVAVVIFSATGKLFDYSSSS

Lambert_DAM5 MRNKIKIKKIDYLPARQVTFSKRRRGLFKKAAELSVLCESEVAVVIFSATGKLFDYSSSS

Napoleon_DAM5 MRNKIKIKKIDYLPARQVTFSKRRRGLFKKAAELSVLCESEVAVVIFSATGKLFDYSSSS

Rainier_DAM5 MRNKIKIKKIDYLPARQVTFSKRRRGLFKKAAELSVLCESEVAVVIFSATGKLFDYSSSS

Regina_DAM5 MRNKIKIKKIDYLPARQVTFSKRRRGLFKKAAELSVLCESEVAVVIFSATGKLFDYSSSS

Sam_DAM5 MRNKIKIKKIDYLPARQVTFSKRRRGLFKKAAELSVLCESEVAVVIFSATGKLFDYSSSS

Satonishiki_DAM5 MRNKIKIKKIDYLPARQVTFSKRRRGLFKKAAELSVLCESEVAVVIFSATGKLFDYSSSS

Sue_DAM5 MRNKIKIKKIDYLPARQVTFSKRRRGLFKKAAELSVLCESEVAVVIFSATGKLFDYSSSS

Summit_DAM5 MRNKIKIKKIDYLPARQVTFSKRRRGLFKKAAELSVLCESEVAVVIFSATGKLFDYSSSS

Vic_DAM5 MRNKIKIKKIDYLPARQVTFSKRRRGLFKKAAELSVLCESEVAVVIFSATGKLFDYSSSS

------------------------------------------------------*-----

Ambrunes_DAM5 TKDVIEKYNVHMNGVEKLNDQEIELQLEHENHIKLSKELEEKSRQLRQMKGDDLEGLNLD

Brooks_DAM5 TKDVIEKYNVHMNGVEKLNDQEIELQLEHENHIKLSKELEEKSRQLRQMKGDDLEGLNLD

Cristobalina_DAM5 TKDVIEKYNVRMNGVEKLNDQEIELQLEHENHIKLSKELEEKSRQLRQMKGDDLEGLNLD

Ferrovia_DAM5 TKDVIEKYNVHMNGVEKLNDQEIELQLEHENHIKLSKELEEKSRQLRQMKGDDLEGLNLD

Helderfinger_DAM5 TKDVIEKYNVHMNGVEKLNDQEIELQLEHENHIKLSKELEEKSRQLRQMKGDDLEGLNLD

Lambert_DAM5 TKDVIEKYNVHMNGVEKLNDQEIELQLEHENHIKLSKELEEKSRQLRQMKGDDLEGLNLD

Napoleon_DAM5 TKDVIEKYNVHMNGVEKLNDQEIELQLEHENHIKLSKELEEKSRQLRQMKGDDLEGLNLD

Rainier_DAM5 TKDVIEKYNVHMNGVEKLNDQEIELQLEHENHIKLSKELEEKSRQLRQMKGDDLEGLNLD

Regina_DAM5 TKDVIEKYNVHMNGVEKLNDQEIELQLEHENHIKLSKELEEKSRQLRQMKGDDLEGLNLD

Sam_DAM5 TKDVIEKYNVHMNGVEKLNDQEIELQLEHENHIKLSKELEEKSRQLRQMKGDDLEGLNLD

Satonishiki_DAM5 TKDVIEKYNVHMNGVEKLNDQEIELQLEHENHIKLSKELEEKSRQLRQMKGDDLEGLNLD

Sue_DAM5 TKDVIEKYNVHMNGVEKLNDQEIELQLEHENHIKLSKELEEKSRQLRQMKGDDLEGLNLD

Summit_DAM5 TKDVIEKYNVHMNGVEKLNDQEIELQLEHENHIKLSKELEEKSRQLRQMKGDDLEGLNLD

Vic_DAM5 TKDVIEKYNVHMNGVEKLNDQEIELQLEHENHIKLSKELEEKSRQLRQMKGDDLEGLNLD

----------*-------------------------------------------------

Ambrunes_DAM5 ELLKLEQLVEASLGRVMETKEELIKSEIMALERKGAELVEANNQLRQTMVMLSAGNTGPA

Brooks_DAM5 ELLKLEQLVEASLGRVMETKEELIKSEIMALERKGAELVEANNQLRQTMVMLSAGNTGPA

Cristobalina_DAM5 ELLKLEQLVEASLGRVMETKEELIKSEIMALERKGAELVEANNQLRQTMVMLSAGNTGPA

Ferrovia_DAM5 ELLKLEQLVEASLGRVMETKEELIKSEIMALERKGAELVEANNQLRQTMVMLSAGNTGPA

Helderfinger_DAM5 ELLKLEQLVEASLGRVMETKEELIKSEIMALERKGAELVEANNQLRQTMVMLSAGNTGPA

Lambert_DAM5 ELLKLEQLVEASLGRVMETKEELIKSEIMALERKGAELVEANNQLRQTMVMLSAGNTGPA

Napoleon_DAM5 ELLKLEQLVEASLGRVMETKEELIKSEIMALERKGAELVEANNQLRQTMVMLSAGNTGPA

Rainier_DAM5 ELLKLEQLVEASLGRVMETKEELIKSEIMALERKGAELVEANNQLRQTMVMLSAGNTGPA

Regina_DAM5 ELLKLEQLVEASLGRVMETKEELIKSEIMALERKGAELVEANNQLRQTMVMLSAGNTGPA

Sam_DAM5 ELLKLEQLVEASLGRVMETKEELIKSEIMALERKGAELVEANNQLRQTMVMLSAGNTGPA

Satonishiki_DAM5 ELLKLEQLVEASLGRVMETKEELIKSEIMALERKGAELVEANNQLRQTMVMLSAGNTGPA

Sue_DAM5 ELLKLEQLVEASLGRVMETKEELIKSEIMALERKGAELVEANNQLRQTMVMLSAGNTGPA

Summit_DAM5 ELLKLEQLVEASLGRVMETKEELIKSEIMALERKGAELVEANNQLRQTMVMLSAGNTGPA

Vic_DAM5 ELLKLEQLVEASLGRVMETKEELIKSEIMALERKGAELVEANNQLRQTMVMLSAGNTGPA

------------------------------------------------------------

Ambrunes_DAM5 LMDPERLNNNIEGGGEEEGMSAESAISTTCNSAVSLSLENDSSDEVTLSLKLGRLQLRNP

Brooks_DAM5 LMDPERLNNNIEGGGEEEGMSAESAISTTCNSAVSLSLEDDSSDEVTLSLKLGRLQLRNP

Cristobalina_DAM5 HMDPERLNNNIEGGGEEEGMSAESAISTTCNSAVSLSLEDDSSDEVTLSLKLGRLQLRNP

Ferrovia_DAM5 LMDPERLNNNIEGGGEEEGMSAESAISTTCNSAVSLSLEDDSSDEVTLSLKLGRLQLRNP

Helderfinger_DAM5 LMDPERLNNNIEGGGEEEGMSAESAISTTCNSAVSLSLEDDSSDEVTLSLKLGRLQLRNP

Lambert_DAM5 LMDPERLNNNIEGGGEEEGMSAESAISTTCNSAVSLSLEDDSSDEVTLSLKLGRLQLRNP

Napoleon_DAM5 LMDPERLNNNIEGGGEEEGMSAESAISTTCNSAVSLSLENDSSDEVTLSLKLGRLQLRNP

Rainier_DAM5 LMDPERLNNNIEGGGEEEGMSAESAISTTCNSAVSLSLEDDSSDEVTLSLKLGRLQLRNP

Regina_DAM5 LMDPERLNNNIEGGGEEEGMSAESAISTTCNSAVSLSLEDDSSDEVTLSLKLGRLQLRNP

Sam_DAM5 LMDPERLNNNIEGGGEEEGMSAESAISTTCNSAVSLSLEDDSSDEVTLSLKLGRLQLRNP

Satonishiki_DAM5 LMDPERLNNNIEGGGEEEGMSAESAISTTCNSAVSLSLEDDSSDEVTLSLKLGRLQLRDP

Sue_DAM5 LMDPERLNNNIEGGGEEEGMSAESAISTTCNSAVSLSLEDDSSDEVTLSLKLGRLQLRNP

Summit_DAM5 LMDPERLNNNIEGGGEEEGMSAESAISTTCNSAVSLSLENDSSDEVTLSLKLGRLQLRNP

Vic_DAM5 LMDPERLNNNIEGGGEEEGMSAESAISTTCNSAVSLSLEDDSSDEVTLSLKLGRLQLRNP

*--------------------------------------*------------------*-

**Supplementary Figure 1** Continued.

Ambrunes_DAM5 DIERG

Brooks_DAM5 DIERG

Cristobalina_DAM5 DIERG

Ferrovia_DAM5 DIERG

Helderfinger_DAM5 DIERG

Lambert_DAM5 DIERG

Napoleon_DAM5 DIERG

Rainier_DAM5 DIERG

Regina_DAM5 DIERG

Sam_DAM5 DIERG

Satonishiki_DAM5 DIERG

Sue_DAM5 DIERG

Summit_DAM5 DIERG

Vic_DAM5 DIERG

-----

1 10 20 30 40 50 60

| | | | | | |

Ambrunes_DAM6 MVKMMREKIKIKKIDYLPARQVTFSKRRRGLFKKAAELSVLCESEVAVVIFSATGKLFDY

Brooks_DAM6 MVKMMREKIKIKKIDYLPARQVTFSKRRRGLFKKAAELSVLCESEVAVVIFSATGKLFDY

Cristobalina_DAM6 MVKMMREKIKIKKIDYLPARQVTFSKRRRGLFKKAAELSVLCESEVAVVIFSATGKLFDY

Ferrovia_DAM6 MVKMMREKIKIKKIDYLPARQVTFSKRRRGLFKKAAELSVLCESEVAVVIFSATGKLFDY

Helderfinger_DAM6 MVKMMREKIKIKKIDYLPARQVTFSKRRRGLFKKAAELSVLCESEVAVVIFSATGKLFDY

Lambert_DAM6 MVKMMREKIKIKKIDYLPARQVTFSKRRRGLFKKAAELSVLCESEVAVVIFSATGKLFDY

Napoleon_DAM6 MVKMMREKIKIKKIDYLPARQVTFSKRRRGLFKKAAELSVLCESEVAVVIFSATGKLFDY

Rainier_DAM6 MVKMMREKIKIKKIDYLPARQVTFSKRRRGLFKKAAELSVLCESEVAVVIFSATGKLFDY

Regina_DAM6 MVKMMREKIKIKKIDYLPARQVTFSKRRRGLFKKAAELSVLCESEVAVVIFSATGKLFDY

Sam_DAM6 MVKMMREKIKIKKIDYLPARQVTFSKRRRGLFKKAAELSVLCESEVAVVIFSATGKLFDY

Satonishiki_DAM6 MVKMMREKIKIKKIDYLPARQVTFSKRRRGLFKKAAELSVLCESEVAVVIFSATGKLFDY

Sue_DAM6 MVKMMREKIKIKKIDYLPARQVTFSKRRRGLFKKAAELSVLCESEVAVVIFSATGKLFDY

Summit_DAM6 MVKMMREKIKIKKIDYLPARQVTFSKRRRGLFKKAAELSVLCESEVAVVIFSATGKLFDY

Vic_DAM6 MVKMMREKIKIKKIDYLPARQVTFSKRRRGLFKKAAELSVLCESEVAVVIFSATGKLFDY

------------------------------------------------------------

Ambrunes_DAM6 SSSSIEDVIERYKAHTNGVEKSNKQFLELQLENEKHIKLSKELEEKSRQLRQMKGEDLEG

Brooks_DAM6 SSSSIEDVLERYKAHTNGVEKSNKQFLELQLENEKHIKLSKELEEKSRQLRQMKGEDLEG

Cristobalina_DAM6 SSSSIEDVIERYKAHTNGVEKSNKQFLELQLENEKHIKLSKELEEKSRQLRQMKGEDLEG

Ferrovia_DAM6 SSSSIEDVIERYKAHTNGVEKSNKQFLELQLENEKHIKLSKELEEKSRQLRQMKGEDLEG

Helderfinger_DAM6 SSSSIEDVIERYKAHTNGVEKSNKQFLELQLENEKHIKLSKELEEKSRQLRQMKGEDLEG

Lambert_DAM6 SSSSIEDVIERYKAHTNGVEKSNKQFLELQLENEKHIKLSKELEEKSRQLRQMKGEDLEG

Napoleon_DAM6 SSSSIEDVIERYKAHTNGVEKSNKQFLELQLENEKHIKLSKELEEKSRQLRQMKGEDLEG

Rainier_DAM6 SSSSIEDVLERYKAHTNGVEKSNKQFLELQLENEKHIKLSKELEEKSRQLRQMKGEDLEG

Regina_DAM6 SSSSIEDVIERYKAHTNGVEKSNKQFLELQLENEKHIKLSKELEEKSRQLRQMKGEDLEG

Sam_DAM6 SSSSIEDVIERYKAHTNGVEKSNKQFLELQLENEKHIKLSKELEEKSRQLRQMKGEDLEG

Satonishiki_DAM6 SSSSIEDVIERYKAHTNGVEKSNKQFLELQLENEKHIKLSKELEEKSRQLRQMKGEDLEG

Sue_DAM6 SSSSIEDVIERYKAHTNGVEKSNKQFLELQLENEKHIKLSKELEEKSRQLRQMKGEDLEG

Summit_DAM6 SSSSIEDVIERYKAHTNGVEKSNKQFLELQLENEKHIKLSKELEEKSRQLRQMKGEDLEG

Vic_DAM6 SSSSIEDVLERYKAHTNGVEKSNKQFLELQLENEKHIKLSKELEEKSRQLRQMKGEDLEG

--------*---------------------------------------------------

Ambrunes_DAM6 LNLDELLKLEQLVEGSLGRVIETKEELIMSEIMSLEKKGAELVETNNQLRQRMAMLSGGN

Brooks_DAM6 LNLDELLKLEQLVEGSLGRVIETKEELIMSEIMSLEKKGAELVETNNQLRQRMAMLSGGN

Cristobalina_DAM6 LNLDELLKLEQLVEGSLGRVIETKEELIMSEIMSLEKKGAELVETNNQLRQRMAMLSGGN

Ferrovia_DAM6 LNLDELLKLEQLVEGSLGRVIETKEELIMSEIMSLEKKGAELVETNNQLRQRMAMLSGGN

Helderfinger_DAM6 LNLDELLKLEQLVEGSLGRVIETKEELIMSEIMSLEKKGAELVETNNQLRQRMAMLSGGN

Lambert_DAM6 LNLDELLKLEQLVEGSLGRVIETKEELIMSEIMSLEKKGAELVETNNQLRQRMAMLSGGN

Napoleon_DAM6 LNLDELLKLEQLVEGSLGRVIETKEELIMSEIMSLEKKGAELVETNNQLRQRMAMLSGGN

Rainier_DAM6 LNLDELLKLEQLVEGSLGRVIETKEELIMSEIMSLEKKGAELVETNNQLRQRMAMLSGGN

Regina_DAM6 LNLDELLKLEQLVEGSLGRVIETKEELIMSEIMSLEKKGAELVETNNQLRQRMAMLSGGN

Sam_DAM6 LNLDELLKLEQLVEGSLGRVIETKEELIMSEIMSLEKKGAELVETNNQLRQRMAMLSGGN

Satonishiki_DAM6 LNLDELLKLEQLVEGSLGRVIETKEELIMSEIMSLEKKGAELVETNNQLRQRMAMLSGGN

Sue_DAM6 LNLDELLKLEQLVEGSLGRVIETKEELIMSEIMSLEKKGAELVETNNQLRQRMAMLSGGN

Summit_DAM6 LNLDELLKLEQLVEGSLGRVIETKEELIMSEIMSLEKKGAELVETNNQLRQRMAMLSGGN

Vic_DAM6 LNLDELLKLEQLVEGSLGRVIETKEELIMSEIMSLEKKGAELVETNNQLRQRMAMLSGGN

------------------------------------------------------------

**Supplementary Figure 1** Continued.

Ambrunes_DAM6 TGPALVEPETLNTNIGGGGEDGMSSESATMATSTSCNSALSLSLEDDCSDVTLSLKLGLP

Brooks_DAM6 TGPALVEPETLNTNIGGGGEDGMSSESATMATSTSCNSALSLSLEDDCSDVTLSLKLGLP

Cristobalina_DAM6 TGPALVEPETLNTNIGGGGEDGMSSESATMATSTSCNSALSLSLEDDCSDVTLSLKLGLP

Ferrovia_DAM6 TGPALVEPETLNTNIGGGGEDGMSSESATMATSTSCNSALSLSLEDDCTDVTLSLKLGLP

Helderfinger_DAM6 TGPALVEPETLNTNIGGGGEDGMSSESATMATSTSCNSALSLSLEDDCSDVTLSLKLGLP

Lambert_DAM6 TGPALVEPETLNTNIGGGGEDGMSSESATMATSTSCNSALSLSLEDDCSDVTLSLKLGLP

Napoleon_DAM6 TGPALVEPETLNTNIGGGGEDGMSSESATMATSTSCNSALSLSLEDDCSDVTLSLKLGLP

Rainier_DAM6 TGPALVEPGTLNTNIGGGGEDGMSSESATMATSTSCNSALSLSLEDDCSDVTLSLKLGLP

Regina_DAM6 TGPALVEPETLNTNIGGGGEDGMSSESATMATSTSCNSALSLSLEDDCSDVTLSLKLGLP

Sam_DAM6 TGPALVEPETLNTNIGGGGEDGMSSESATMATSTSCNSALSLSLEDDCSDVTLSLKLGLP

Satonishiki_DAM6 TGPALVEPETLNTNIGGGGEDGMSSESATMATSTSCNSALSLSLEDDCSDVTLSLKLGLP

Sue_DAM6 TGPALVEPETLNTNIGGGGEDGMSSESATMATSTSCNSALSLSLEDDCSDVTLSLKLGLP

Summit_DAM6 TGPALVEPETLNTNIGGGGEDGMSSESATMATSTSCNSALSLSLEDDCSDVTLSLKLGLP

Vic_DAM6 TGPALVEPETLNTNIGGGGEDGMSSESATMATSTSCNSALSLSLEDDCSDVTLSLKLGLP

--------*---------------------------------------*-----------


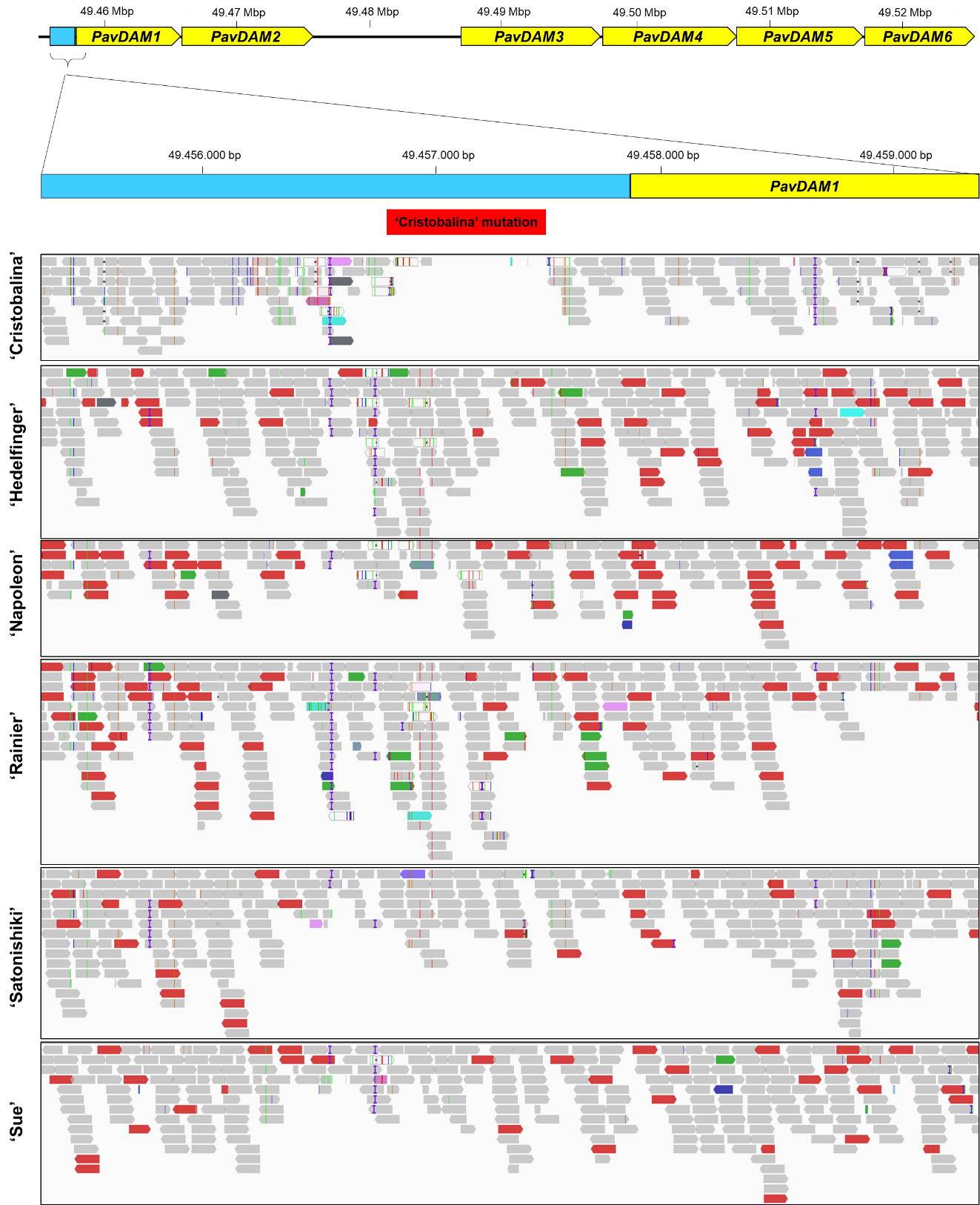


**Supplementary Figure 2** Alignment of gDNA sequences of ‘Cristobalina’, ‘Hedelfinger’, ‘Napoleon’, ‘Rainier’, ‘Satonishiki’ and ‘Sue’ to ‘Regina’ sweet cherry genome sequence (Le Dantec et al., 2020) upstream of *PavDAM1* gene (49,455,295 to 49,459,399 bp).


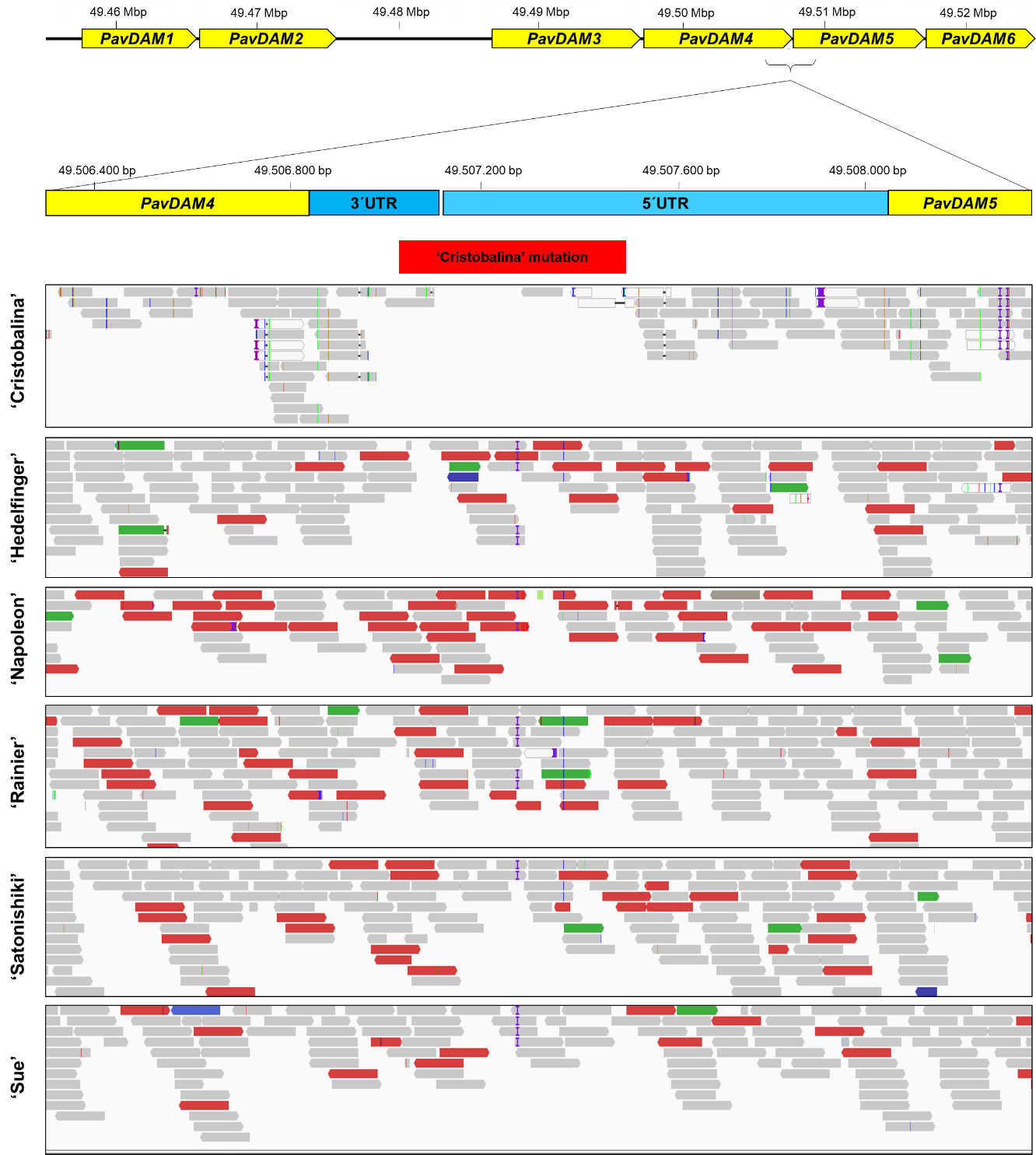


**Supplementary Figure 3** Alignment of gDNA sequences of ‘Cristobalina’, ‘Hedelfinger’, ‘Napoleon’, ‘Rainier’, ‘Satonishiki’ and ‘Sue’ to ‘Regina’ sweet cherry genome sequence (Le Dantec et al., 2020) in the region between of *PavDAM4* and *-5* gene (49,506,293 to 49,508,244 bp).

**Supplementary Figure 4** Alignment of ‘Cristobalina’ and ‘Regina’ consensus sequences upstream of *PavDAM1,* generated from Sanger sequencing of PCR products (primers D1Sf/D1Sr). Numbers indicate position upstream of *PavDAM1* start codon. Deletions are marked by gaps and differences are marked with asterisks. Motifs (NewPLACE database, see Sup Table 7) potentially related to *PavDAM* expression found in ‘Regina’ and absent in ‘Crsitobalina’ deletion are shown in yellow.

Cristobalina    832 TACTCTTTCTTTCTCTTGGTTGA-CCAAAAAAAAAACCTCTTTGATGAATTGTTTGTTCC 773
Regina         1600 TACTCTTTCTTTCTCTTGGTTGACCCAAAAAAAAAACCTCTTTGATGAATTGTTTGTTCC 1540

·······················*····································

Cristobalina    772 TCARAGAATATCACAACAACAATAGCAAAACAATGC------------------------ 736
Regina         1539 TCAAAGAATATCACAACAACAATAGCAAAACAATCCATATGGGTCATATATCCATGGCTT 1479

···*································************************

Cristobalina    736 ------------------------------------------------------------ 736 
Regina         1478 TTTCTCGCCCGAGTCTCATGTTTGTACAAATCACATGTCTCCCATCCCATACATCAAAAA 1357 
 ************************************************************

Cristobalina    736 ------------------------------------------------------------ 736 
Regina         1356 GCTCTTTTGCACTGAATAGATTGAAAAGAGATGTTATAATCATGCAACCAAGCCAACAAA 1296 
 ************************************************************

Cristobalina    736 ------------------------------------------------------------ 736 
Regina         1295 ACTGGATGAATACAAAACAACAAATAAATGGCAAAAGAATACGAAAGGCACCGATTGAAG 1235 
 ************************************************************

Cristobalina    736 ------------------------------------------------------------ 736 
Regina         1234 TGCCCTCAGTTTTCTCTCATGTCAAAAAGTCAGAAGCTTCATAACGTAATCAAAGAAAAA 1174 
 ************************************************************

Cristobalina    736 ------------------------------------------------------------ 736 
Regina         1173 GAGACGAACCCATCACAGCCCCACCAACAAATTGAAGAGCAGGATCAGAACGACAACGAA 1113 
 ************************************************************

Cristobalina    736 ------------------------------------------------------------ 736 
Regina         1112 ATAGAAGAAGTCCAGTGCCAAATAGCAGTCTCCAACTTCCCTGGCGGCTCCGAGACCTTC 1052 
 ************************************************************

Cristobalina    736 ------------------------------------------------------------ 736
Regina         1051 GAATAGCAGCCAAGTTCATCAAGATCTAGGTGGTGGAGTTTACTGGGTTGGGGAGATTGG 991 
 ************************************************************

Cristobalina    736 ------------------------------------------------------------ 736 
Regina          990 GGTGTGTGTGGTGGTTGGCGGGTGGGCTGGTGAGGGTGGGTTTGGCTGATGGCTTCTCTT 930 
 ************************************************************

Cristobalina    736 ------------------------------------------------------------ 736
Regina          929 CCCCTTATATTTTTCTTCTTTTTAGAAAAGAAAAAATCTTTCTTGTTCTTAGATTTAAAT 869 
 ************************************************************

Cristobalina    736 -----------------------------------------------------------  736  
Regina          868 GCCCCTCAACAAGTAACCTCAGTTTAATCTAATTTGATCTAATTTGACAGAAATTGGAGA 808 
 ************************************************************

Cristobalina    736 ------------------------------------------------------------ 736  
Regina          807 TTGAACTTGAATTGCTTAAATTGAAAATCACAAAGGTAAAAATAATTAAATTGAAAACAC 747 
 ************************************************************

Cristobalina    736 ----------ATGGTAAAAGTGATAAACTACATAGACCAAAAATGATAAAATAGGCACGT 686
Regina          746 AGAAATTTTAATGGTAAAAGTGATAAACCACATAGACCAAAAATGATAAAATAGGCACGT 686 
 **********··················································

Cristobalina    685 GAGAATTGAACCCGGCGTGGACTGTGGGAGCAACAGAAGAAGTGTCGCATGGTGAGTTCA 625 Regina          685 GAGAATTGAACCCGACGTAGACTGTGGGAGCAACCGAAGAAGTGTCGCATGGTGAGTTCA 625

····························································

Cristobalina    624 ATCAAGTGAGACGACAGCGCATCACCAGTATCAAGGACCATCCTCCTCCACATAAAAGCA 564

Regina          624 ATCAAGTGAGACGACAGCGCATCACCAGTATCAAGGACCATCCTCCTCCACATAAAAGCA 564

····························································

Cristobalina    563 CAAATTTTATTTATTTATTTTTTATAAAGATATTAAACTTAGATATGGACAAAACCATTC 403

Regina          563 CAAATTTTATTTATTTATTTTTTATAAAGATATTAAACTTAGATATGGACAAAACCATTC 403

····························································

Cristobalina    502 ACAAAAGTAAGATGCCACATTTTCTACTGTCACGTTACAAAATCAACGGTGGAGATCAAA 442
Regina          502 ACAAAAGTAAGATGCCACATTTTCTACTGTCACGTTACAAAATCAACGGTGGAGATCAAA 442 
 ····························································

Cristobalina    441 TTAATCGAAAAATAGATTTTTTTTTCTTCCATAAAATGGAAAATTGCCTTTGAAATCTTC 381
Regina          441 TTAATCGAAAAATAGATTTTTTTTCTTTCCATAAAATGGAAAATTGCCTTTGAAATCTTC 381 
 ····························································

Cristobalina    380 CCTTTCTCGAACAGCTAGCCAGCAGCAGCAGCAGCCAACTCTCCCTCTCTCTCATTCTCT 320 
Regina          380 CCTTTCTCGAACAGCTAGCCAGCAGCAGCAGCAGCCAACTCTCCCTCTCTCTCATTCTCT 320 
 ····························································

Cristobalina    319 TAGGCTTCARACCCTGAAACCCGACAAAGGTAAACATTAAACAAAGAGAGGAAACCCARA 259 
Regina          319 TAGGCTTCAGACCCTGAAACCCGACAAAGGTAAACATTAAACAAAGAGAGGAAACCCAGA 259 
 ·········*················································*·

Cristobalina    258 ATTTAATTAGTTGATTAATTAATGGTTTTCCCTCTTTCTCTTCTTCTTCTTTGTGCTCTG 198 
Regina          258 ATTTAATTAGTTGATTAATTAATGGTTTTCCCTCTTTCTCTTCTTCTTCTTTGTGCTCTG 198 
 ····························································

Cristobalina    197 GTACTCTCTAGGCATGTTGTTGTGAACTTGTGACCTATTTTGGTTGGTGGGTTTTTCTGG 137 
Regina          197 GTACTCTCTAGGCATGTTGTTGTGAACTTGTTACCTATTTTGGTTGGTGGGTTTTTCTGG 137 
 ····························································

Cristobalina    136 GTTTTATTCACTTAGATCTGGGGGCCATTAAATCTTTAAAATTTACAAGAAACCCAGAAA 76 
Regina          136 GTTTTATTCACTTAGATCTGGGGGCCATTAAATCTTTAAAATTTACAAGAAACCCAGAAA 76 
 ····························································

Cristobalina     75 ATCATTTGTAGTTTTTGAGTGTATGAACATAATATATGTGAAAAGTGGTTGGTTTGAATT 15
Regina          75 ATCATTTGTAGTTTTTGAGTGTATGAACATAATATATGTGAAAAGTGGTTGGTTTGAATT 15

····························································

Cristobalina     14 TTTTTGAAGGGGAC 1 
Regina          14 TTTTTGAAGGGGAC 1

··············

**Supplementary Figure 5** Alignment of ‘Cristobalina’ and ‘Regina’ PAvDAM 4 and -5 contiguous UTR consensus sequences generated from Sanger sequencing of PCR products (primers PavD4/5Mf/PavD4/5Mr). Numbers indicate location upstream of *PavDAM5* start codon. Deletions are marked by gaps and differences are marked with asterisks. *PavDAM4* 3’ UTR and *PavDAM5* 5’ UTR regions are marked in blue and grey, respectively.

Cristobalina -TCCTTGGTTAAGAAGTGGAGAAAGATGAAAGTTTACTCTCCTTATAGCTAAATAAAGAA

Regina 1220 ATCCTTGGTTAAGAAGTGAAGAAAGATGAAAGTTTACTTTCCTTATAGCTAAATAAAGAA 1160

*·················*···················*·····················

Cristobalina AGTTGTGGTATTGTGAGATATCG-GTGTTTTAACTTGATACGAGATTGAAAAATATTGAA

Regina 1159 AGTTGTGGTATTGTGAGATATCGTGTGTTTTAACTTGATAAGGGATTGAAAAATATTCAA 1099

·······················*················*·*··············*··

Cristobalina GGGAGAGTTAACTTTGTGTGTTGTATCAAAACATGTGTATTACATGTAACCTGACATATT

Regina 1098 GGGAGAGTTAACTTTGTGT-TTGTATCAAAACATG------------------------- 1063

···················*···············*************************

Cristobalina GCTCTTTGCACTTTGTATTGTATTTAGAACTCTGTATCAATGCCAGCCTGCATCGACAGA

Regina 1062 -----TTGCACTTTGTATTGTATTTAGAACTYTGTATCAATGC-AGCCTGCATCGACAGA 1008

*****······································*················

Cristobalina CTTGTTAATTTTGAATTGTTTGGGT-TATCAATGTCACAGCACAGGCTAACGTTACGAAA

Regina 1007 CTTGTTAATTTTGTATTGTTTGGGTTTATCAATG---CAGC------------------- 969

·············*···········*········***····*******************

Cristobalina GGTCTCGTTGGGTACGCGGATCGTAGACTTATATGTCTTTCTTCATTGTTTTCACGTTAC

Regina 968 ---------------------------CTTCATTGTCTTTCTTCATTGTTTTCACGTTAC 935

***************************···***···························

Cristobalina AAAGCTCATTTCAAGATCTTGTGAAGTTTAGTCCCACGAATCACGTTATAGGTAAAATTG

Regina 934 AAAGCTCATT---------------------TCCCACGATTGACGTTATAGGTAAAATTG 895

··········*********************········*·*··················

Cristobalina TTTATTTATGCTATTACAACTCATGTACGCCAAAACTTGAACCTGAAACAAATAGAACTA

Regina 894 TTTATTTATACTATTACAACTCATGTACACCTAAACTTAAACC--AGACAACTACAAGTA 835

·········*··················*··*······*····**·*····*··*··*··

Cristobalina AAATAAATTTTAAATTCATTTAAGATGTAT-TTTTTTCTTTCTTTAA--TTGCTCATTTC

Regina 834 AAATAAATTTTAAATTCATTTCAGATGTTTCTTTTTTTTTTCTTTAATTTTGCTCATTTC 774

·····················*······*·*······*·········**···········

Cristobalina CTAAAGATTACATGTAGGACACCAGAATGTTTAAGAAGAAACTCATATCGCGAGACTCGT

Regina 773 CTAAAGATTACATGTAGGACACCAGAATGTTTAAGAAGAAACTCATATCGCGAGACTCGT 713

····························································

Cristobalina ATTGTCTGCGTGGATTAAATGAATTGAAGTGGACTGGGGGAAGTAACCTGAGCAAGATAA

Regina 712 ATTGTCTGTGTGGATTAAATGAATTGAAGTGG----------------------AGATAA 674

········*·······················**********************······

Cristobalina AGACAAAGCCACGTGTCATGGTGGGCTCAATCAAGTGGCATGACAGCGTATGGCCTGTGT

Regina 673 AGACAAAGCCACGTGTCATGGTGGGCTCAATCAAGTGGCATGACAGCGTATGGCCTGTGT 613

····························································

Cristobalina CAAAGTGCCATCCTCCACACC------------------TTCTTTCTTTTGTAGCCTGAC

Regina 612 CAAAGTGCCATCCTCCACACCTAAAAAAAATTATTATTCTTCTTTCTTTTGTAGCCTGAC 552

·····················******************·····················

Cristobalina ACGTAATGAGAATTTTACCGACGAAAAAAACAAAACTTAGATATTTATGACAAACTCATT

Regina 551 ACGTAATCARAATTTTACCGACGAAAAAAACAAAACTTARATATTTATGACAAACTCATT 489

·······*····················································

Cristobalina AA---CAAGCAAGATGCCACATTTTCTCACGTCAGATATCAACGGTGGAGATTGAATTCA

Regina 490 AACAACAAGCAARATGCCACANTTTCTCACGTCAAATATCAACGGTGGARATTGAATTCA 430

··***·····························*·························

Cristobalina TCAACGTCA

Regina 429 TCAACGTCA 421

**Supplementary Figure 6** Comparison of *PavDAMs* expression profiling during dormancy in ‘Regina’ and ‘Cristobalina’. Data from Vimont et al. (2019) (bwenden.shinyapps.io/DorPatterns).


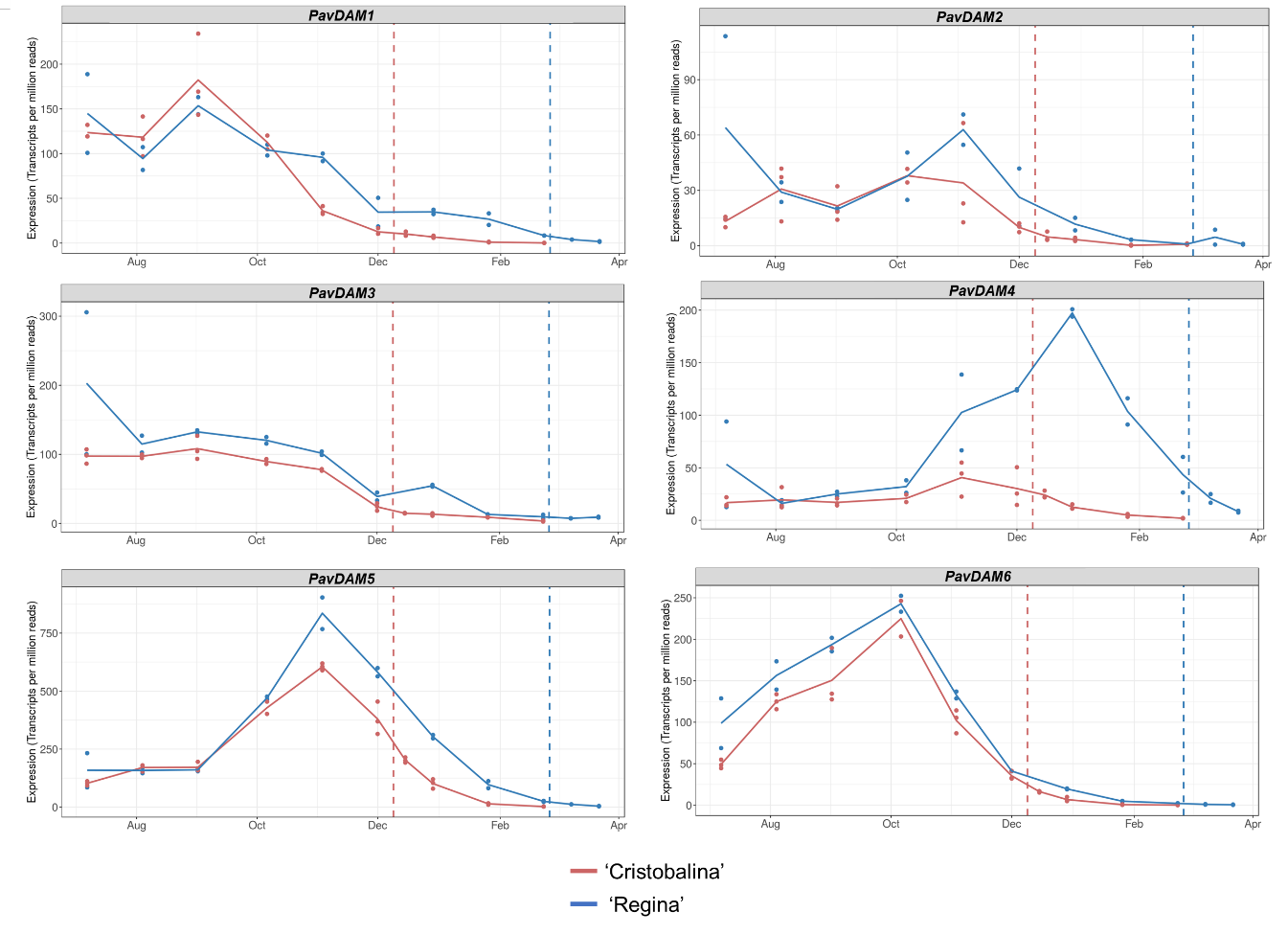

Supplement: Supplementary file 1 [file Data_Sheet_1.docx]
